# *APOE2* attenuates *APOE4*-associated amyloid pathology and related toxicity in a mouse model of Alzheimer’s disease

**DOI:** 10.1038/s41467-026-78407-x

**Published:** 2026-10-08

**Authors:** Kai Chen, Yixing Chen, Yingxue Ren, Zhen Wang, Ya Huang, Meixia Pan, Zonghua Li, Monica Castanedes Casey, Wenhui Qiao, Ji Shi, Dehui Kong, Gisela Xhafkollari, Hannah Santhakumar, Allison M. Wetmore, E. Aubrey Thompson, Xusheng Wang, Dennis W. Dickson, David M. Holtzman, Guojun Bu, Xianlin Han, Junmin Peng, Na Zhao

**Affiliations:** 1 Department of Neuroscience, Mayo Clinic, Jacksonville, FL USA; 2 Department of Quantitative Health Sciences, Mayo Clinic, Jacksonville, FL USA; 3 Departments of Structural Biology and Developmental Neurobiology, St. Jude Children’s Research Hospital, Memphis, TN USA; 4 Barshop Institute for Longevity and Aging Studies, University of Texas Health Science Center at San Antonio, San Antonio, TX USA; 5 Department of Cancer Biology, Mayo Clinic, Jacksonville, FL USA; 6 Department of Neurology, University of Tennessee Health Science Center, Memphis, TN USA; 7 Department of Genetics, Genomics and Informatics, University of Tennessee Health Science Center, Memphis, TN USA; 8 Department of Neurology, Hope Center for Neurological Disorders, Knight Alzheimer’s Disease Research Center, Washington University in St. Louis, St. Louis, MO USA; 9 Present Address: Division of Life Science, The Hong Kong University of Science and Technology, Hong Kong, China

**Keywords:** Alzheimer's disease, Alzheimer's disease

## Abstract

The apolipoprotein E (*APOE*) gene is the strongest genetic risk factor for Alzheimer’s disease (AD), influencing amyloid-β (Aβ) aggregation, clearance, and glial function. While *APOE4* promotes Aβ pathology and *APOE2* is protective, how these opposing alleles interact in the heterozygous *APOE2/4* state remains unknown. Here, using littermate-controlled heterozygous *APOE* mice (*APOE2/3*, *APOE3/4*, and *APOE2/4*) alongside *APOE3/3* controls crossed with 5xFAD mice, we show that *APOE3/4* mice exhibit increased Aβ deposition, maladaptive microglial activation, and reduced microglial interactions with plaques. These changes were attenuated in *APOE2/4* mice, accompanied by a brain proteomic profile more similar to those of *APOE3/3* and *APOE2/3* mice. Increased myelin basic protein (MBP) abundance was observed in *APOE2/3* but not *APOE2/4* mice. Plasma lipidomics revealed shared alterations in *APOE3/4* and *APOE2/4* mice, with distinct signatures in *APOE2/3* mice. These findings define allele interaction-dependent effects of *APOE* on brain and systemic phenotypes and provide a framework for understanding the biological consequences of the *APOE2/4* genotype.

## Abstract (web summary)

Chen and colleagues use a heterozygous mouse model carrying human APOE2 and APOE4 alleles to show that APOE2 attenuates APOE4- associated effects on brain amyloid pathology and related cellular and molecular changes.

## Introduction

The human apolipoprotein E (*APOE*) gene encodes three major alleles, *APOE2*, *APOE3*, and *APOE4*, that combine into six genotypes with distinct and powerful effects on Alzheimer’s disease (AD) risk. *APOE4* is the strongest genetic determinant of late-onset AD, increasing risk in a gene dose-dependent manner, whereas *APOE2* confers substantial protection^1–5^. Individuals carrying both alleles (*APOE2/4*), harboring one protective and one risk variant, represent roughly 2–3% of the population but have been largely excluded from genetic, clinical, and mechanistic studies. Limited neuropathological evidence suggests that *APOE2/4* carriers exhibit an intermediate phenotype, showing a 2.7-fold higher AD risk than *APOE3/3* individuals but much lower than *APOE3/4* (6.1-fold) or *APOE4/4* (31-fold) carriers^6^. These data suggest that *APOE2* confers partial protection even in the presence of *APOE4*, although *APOE4*’s deleterious effects tend to dominate. The molecular basis of these opposing alleles, however, remains unknown. Defining which protective functions of *APOE2* persist and which are overridden by *APOE4* is essential for the rational design of APOE-targeted therapies, particularly as emerging gene-editing strategies, including *APOE4*-to-*APOE2* conversion^7^ or *APOE2* over-expression in the *APOE4* background^8^, will inevitably generate *APOE* genotype similar to *APOE2/4*.

Despite its clinical importance, *APOE* heterozygosity as a whole remains poorly understood. Experimental studies have so far relied almost exclusively on homozygous humanized *APOE* mice (*APOE2/2*, *APOE3/3*, *APOE4/4*), which capture isoform-specific mechanisms but not the genetic diversity of humans, where *APOE3/3* ( ~ 60-65%) is most common and *APOE2/3* ( ~ 10%) and *APOE3/4* ( ~ 20%) are far more prevalent than either homozygous *APOE2/2* ( ~ 0.2%) or *APOE4/4* ( ~ 2%) individuals^9^. These mixed genotypes likely produce unique allele interactions not represented by single-allele models.

To address these gaps, we generated *APOE2/3*, *APOE3/4*, and *APOE2/4* mice, along with *APOE3/3* controls, by crossing homozygous human *APOE* knock-in lines^10^ with or without 5xFAD amyloid background, producing littermates that collectively represent ~97% of human *APOE* genotypes. Through integrated biochemical, histological, and multi-omics analyses, we found that *APOE4* results in greater amyloid pathology, glial activation, and synaptic damage in the brain of *APOE3/4* mice relative to *APOE3/3* controls, whereas *APOE2/4* and *APOE2/3* mice exhibited brain phenotypes similar to *APOE3/3* mice. Additionally, *APOE2* increased myelin basic protein (MBP) abundance in *APOE2/3* mice, whereas this effect was diminished by *APOE4* in *APOE2/4* mice. Moreover, systemic lipid alterations in *APOE2/4* mice partially overlapped with those observed in *APOE3/4* animals, while *APOE2/3* showed distinct lipid signatures, compared to *APOE3/3* mice. Together, these findings establish an in vivo framework for understanding how different *APOE* alleles interact to influence amyloid pathogenesis and systemic lipid metabolism, bridging a key translational gap toward precision APOE-targeted therapies.

## Results

### Reduced brain APOE protein levels in *APOE3/4*, but not in *APOE2/4* mice

**Fig. 1:**
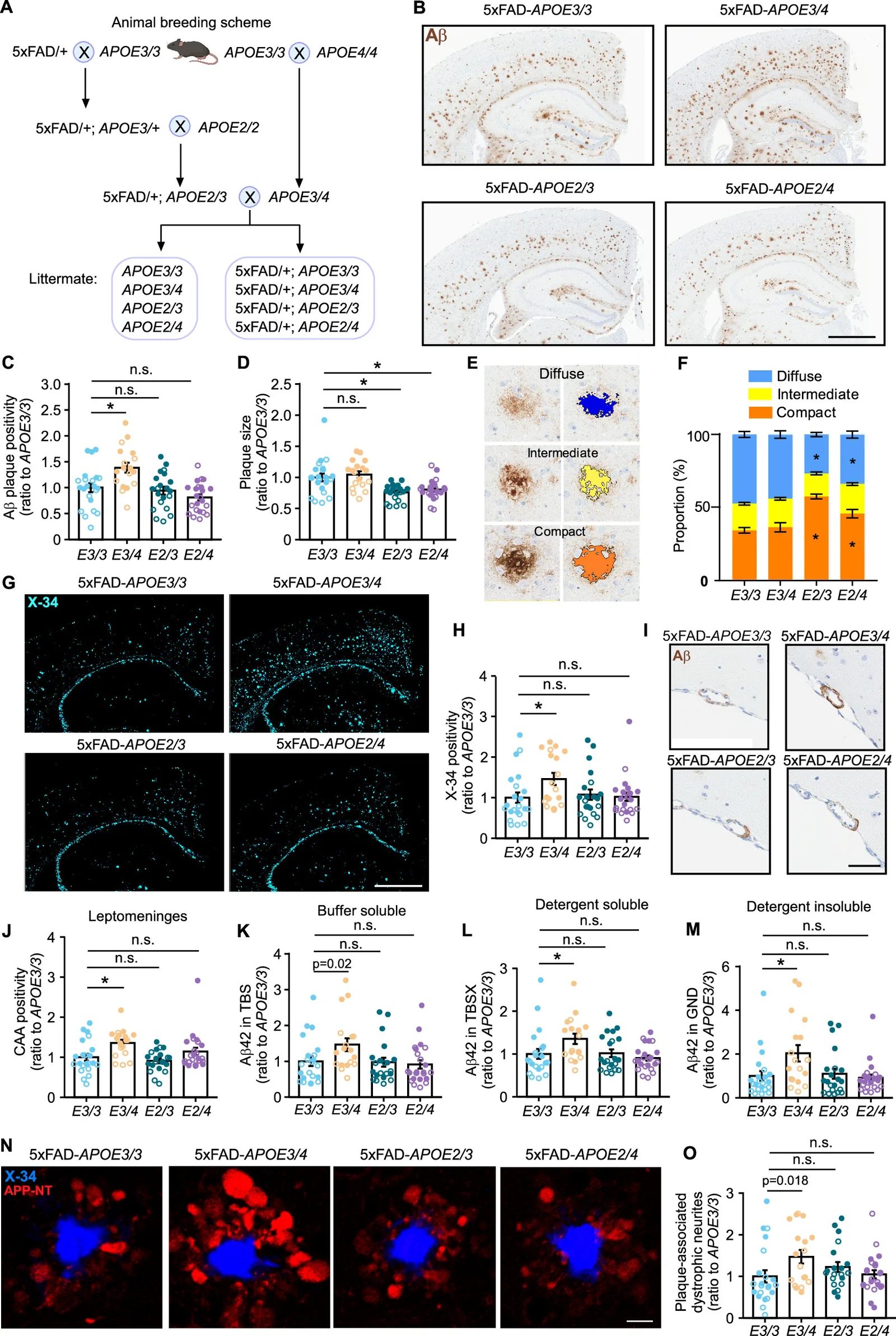
*APOE2* attenuates *APOE4*-driven amyloid deposition and neuritic dystrophy in 5xFAD Mice. **A** Breeding scheme used to generate littermate-controlled mice expressing human *APOE3/3*, *APOE3/4*, *APOE2/3*, or *APOE2/4* genotypes with or without 5xFAD background. The schematic contains elements created with BioRender. **B** Representative brain immunostaining for Aβ in 10-month-old mice. Scale bar, 1 mm. **C**, **D** Quantification of cortical Aβ plaque load (**C**) and average plaque size (**D**) normalized to *APOE3/3* controls. **E**, **F** Representative images (**E**) and quantification (**F**) of plaque morphology classified as diffuse, intermediate, or compact. **G**, **H** Representative X-34 staining of fibrillar amyloid (**G**) and quantification (**H**). Scale bar, 1 mm. **I**, **J** Representative images of leptomeningeal CAA (**I**) and quantification(**J**). Scale bar, 50 µm. **K**–**M** Quantification of Aβ42 levels in the TBS-soluble (**K**), detergent-soluble (TBSX) (**L**), and insoluble (GND) (**M**) fractions by ELISA. **N**, **O** Co-immunofluorescence staining of fibrillar plaques (X-34, blue) and dystrophic neurites (APP-NT, red) (**N**), with quantification of peri-plaque APP-positive area (**O**). Scale bar, 10 µm. Data are presented as mean ± SEM. Sample sizes (number of mice): *E3/3*, *n* = 22; *E3/4*, *n* = 18; *E2/3*, n = 21; *E2/4*, *n* = 23. The data from female and male mice are labeled as solid and open circles, respectively. Statistical significance was determined using a linear model with sex included as a covariate, followed by Bonferroni adjustment for three pairwise comparisons versus *E3/3* (significance threshold, *p* < 0.0167). All pairwise comparisons were two-sided. *p < 0.0167; n.s. not significant. Source data are provided as a Source Data file.

We generated heterozygous human *APOE* knock-in mice (*APOE2/3*, *APOE3/4*, and *APOE2/4*) along with *APOE3/3* controls by stepwise crossing of homozygous *APOE* lines^10^, with or without the 5xFAD transgene, to obtain littermate cohorts (Fig. 1A). At 10 months of age, we first characterized the non-5xFAD littermates for APOE expression. In the brain, cortical *APOE* mRNA levels were comparable across all three heterozygous genotypes relative to *APOE3/3* controls (Supplementary Fig. 1A). At the protein level, Western blot analysis revealed that cortical APOE abundance was reduced by ~25% in *APOE3/4* mice compared to *APOE3/3*, whereas *APOE2/3* and *APOE2/4* mice exhibited similar levels to *APOE3/3* (Supplementary Fig. 1B, C). The lower cortical APOE abundance in *APOE3/4* mice likely reflects the reduced stability of the APOE4 protein, as previously reported^11^. In plasma, APOE protein and cholesterol levels remained comparable across all heterozygous genotypes and their *APOE3/3* littermates (Supplementary Fig. 1D–F).

Together, these findings extend previous observations of reduced brain APOE level in *APOE4/4* mice^12^ by demonstrating that a similar protein reduction is also evident in *APOE3/4* but not in *APOE2/4* mice. This highlights isoform-specific effects of *APOE* heterozygosity on brain APOE abundance, despite comparable plasma APOE and cholesterol levels across genotypes.

### *APOE2* attenuates *APOE4*-driven amyloid deposition and plaque-associated neurotoxicity

We next examined amyloid pathology in 10-month-old 5xFAD littermates carrying different *APOE* genotypes. Quantitative immunostaining of cortical Aβ plaques revealed that *APOE3/4* mice exhibited a significant increase in plaque burden compared with *APOE3/3* controls (Fig. 1B, C). In contrast, *APOE2/4* mice displayed plaque loads comparable to *APOE3/3*, indicating that *APOE2* effectively attenuates the amyloid-promoting effects of *APOE4*. Likewise, *APOE2/3* mice showed plaque levels similar to *APOE3/3*, suggesting that *APOE2* does not further reduce amyloid deposition in the presence of *APOE3* in our model. Morphometric analyses further showed that plaques in *APOE3/4* brains were similar in size to those in *APOE3/3* mice, whereas *APOE2/3* and *APOE2/4* mice exhibited a greater relative proportion of smaller, higher-intensity, and less diffuse Aβ-immunoreactive plaques (Fig. 1D–F), suggesting that *APOE2* may influence plaque maturation and dynamics. Consistent with these observations, X-34 staining, which measures overall fibrillar Aβ burden, was significantly increased in *APOE3/4* mice, whereas *APOE2/3* and *APOE2/4* mice remained comparable to *APOE3/3* controls (Fig. 1G, H).

In addition to parenchymal plaques, we assessed cerebrovascular amyloid pathology by examining leptomeningeal cerebral amyloid angiopathy (CAA). *APOE3/4* mice exhibited significantly greater CAA deposition than *APOE3/3*, while *APOE2/3* and *APOE2/4* mice showed no significant changes (Fig. 1I, J), consistent with the pattern observed for parenchymal Aβ load.

To further assess Aβ solubility, cortical tissues were sequentially fractionated into TBS buffer soluble, TBSX detergent soluble, and detergent insoluble guanidine (GND) fractions. ELISA quantification revealed a significant increase in Aβ42 levels within the TBSX and GND fractions of *APOE3/4* mice, with a numerical increase in the TBS fraction that did not meet the adjusted significance threshold of *p* < 0.0167 (*p* = 0.02), whereas *APOE2/3* and *APOE2/4* mice showed levels comparable to *APOE3/3* controls (Fig. 1K–M). Aβ40 levels were also numerically higher in *APOE3/4* mice across all fractions, although the differences did not meet the adjusted significance threshold of *p* < 0.0167 (TBS, *p* = 0.05; TBSX, *p* = 0.05; GND, *p* = 0.02). Aβ40 levels in *APOE2/3* and *APOE2/4* mice again resembled *APOE3/3* controls (Supplementary Fig. 2A–C). These findings indicate that Aβ42 accumulation is elevated in *APOE3/4* mice, whereas Aβ40 shows a similar but nonsignificant numerical trend. Neither pattern was observed in *APOE2/4* mice, suggesting that *APOE2* modifies *APOE4*-associated molecular changes in the heterozygous state.

Since neuritic dystrophy represents a key indicator of plaque-associated neurotoxicity, we next co-stained brain sections with X-34 (to label fibrillar plaques) and an APP N-terminal antibody (APP-NT) to visualize dystrophic neurites. *APOE3/4* mice showed numerically increased peri-plaque dystrophic neurites in the cortex, although the difference did not meet the adjusted significance threshold of p < 0.0167 (*p* = 0.018). *APOE2/3* and *APOE2/4* mice were comparable to *APOE3/3* controls (Fig. 1N, O).

As *APOE* has been implicated in cerebrovascular dysfunction and blood-brain barrier (BBB) integrity^13–15^, we examined whether genotype-dependent differences in amyloid pathology were accompanied by overt vascular abnormalities. Immunostaining for CD13, GLUT1, and ZO-1 revealed no significant differences among *APOE* genotypes, indicating preserved pericyte-associated coverage, endothelial structure, and tight-junction integrity (Supplementary Fig. 3A–D). We also performed albumin staining to assess potential BBB leakage and did not detect obvious extravascular albumin deposition in the brain parenchyma, suggesting an absence of overt BBB disruption under the conditions examined (Supplementary Fig. 3E).

Because both *APOE* genotype and sex have been reported to influence amyloid pathology^16,17^, and female 5xFAD mice typically develop greater amyloid burden than males^18,19^, all primary analyses were performed using linear models with sex included as a covariate to maximize statistical power while accounting for sex-dependent effects. To determine whether the observed *APOE* genotype-dependent phenotypes differed between sexes, we also performed sex-stratified analyses of the major amyloid-related outcomes. Overall, males and females exhibited similar genotype-dependent patterns across the measured phenotypes (Supplementary Fig. 4A–P). Although several comparisons no longer reached statistical significance after stratification, the direction and magnitude of the effects were largely preserved, consistent with the reduced statistical power resulting from the smaller sample size within each sex. These findings indicate that the *APOE* genotype-associated amyloid phenotypes are broadly consistent in both males and females.

Collectively, these findings demonstrate that *APOE3/4* mice exhibit increased amyloid accumulation and dystrophic neurites compared with *APOE3/3* controls, indicating that the *APOE4* allele remains detrimental in the presence of *APOE3*. *APOE2/4* mice, despite carrying one copy of *APOE4*, showed amyloid burden, fibrillar deposition, and neuritic dystrophy levels comparable to *APOE3/3* controls, in clear contrast to the exacerbated pathology observed in *APOE3/4* mice. Together, these results suggest that *APOE2* may attenuate amyloid-promoting and neurotoxic effects of *APOE4* in vivo, revealing a protective influence of *APOE2* within the heterozygous context.

### *APOE2* mitigates microglial dysfunction but loses its ability to enhance myelin maintenance in the presence of *APOE4*

Given the differences in amyloid burden and neuritic pathology across genotypes, we next asked whether distinct glial activation states might be associated with these different phenotypic outcomes. It is known that *APOE* is predominantly expressed in glial cells, particularly astrocytes and activated microglia, which play central roles in Aβ accumulation, clearance, and neuroinflammation^3,4^. To elucidate how *APOE* genotype modulates these glial responses, we performed NanoString nCounter transcriptomic profiling on cortical tissue to capture genotype-specific changes across major glial populations, using a panel encompassing 770 genes expressed by microglia, astrocytes, and oligodendrocytes (Supplementary Data 1).

**Fig. 2:**
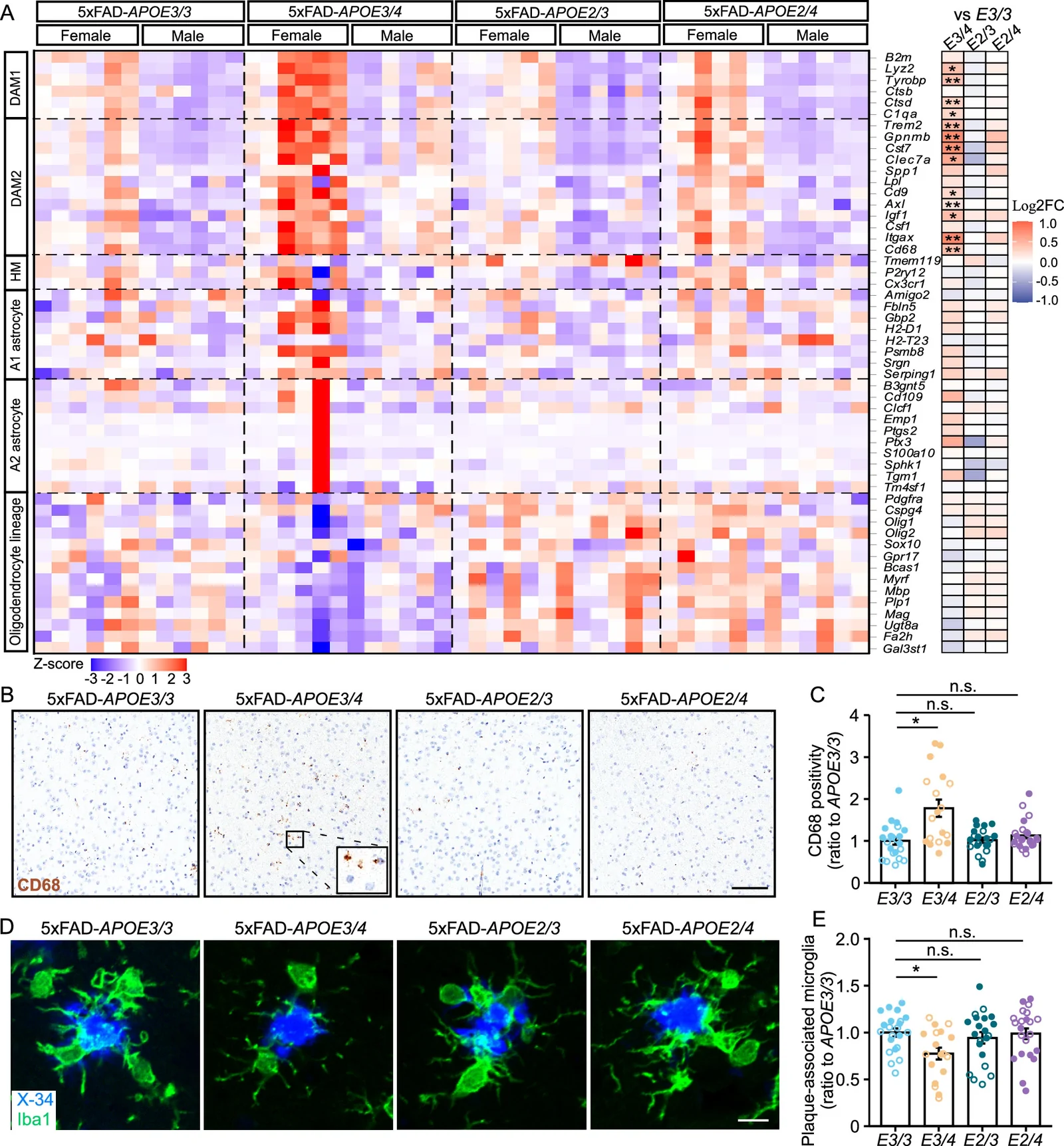
*APOE2* mitigates *APOE4*-associated microglial hyperactivation and preserves microglial responses to amyloid plaques in *APOE2/4* mice. **A** Heatmap from NanoString nCounter transcriptomic profiling showing normalized Z-scores of representative microglial and astrocytic activation markers, as well as oligodendrocyte lineage markers. Right: log2 fold changes (log2FC) of the DEGs relative to *APOE3/3*. Asterisks indicate significant changes after FDR correction. *FDR < 0.05, **FDR < 0.01. *n* = 12 mice/group. Sex was adjusted in the analysis. **B**, **C** Representative CD68 immunostaining in cortex (**B**) and quantification of CD68-positive area (**C**). Scale bar, 100 µm. *E3/3*, *n* = 22 mice; *E3/4*, *n* = 18 mice; *E2/3*, *n* = 21 mice; *E2/4*, *n* = 23 mice. **D**, **E** Co-immunofluorescence staining for Iba1 (green) and fibrillar plaques labeled by X-34 (blue) (**D**), with quantification of plaque-associated microglia (**E**). Scale bar, 10 µm. *E3/3*, *n* = 22 mice; *E3/4*, *n* = 18 mice; *E2/3*, *n* = 21 mice; *E2/4*, *n* = 22 mice. Data in (**C**) and (**E**) are presented as mean ± SEM. Data points from female and male mice are shown as solid and open circles, respectively. Quantifications are normalized to *APOE3/3* controls. Statistical significance was determined using a linear model with sex included as a covariate, followed by Bonferroni adjustment for three pairwise comparisons versus *E3/3* (significance threshold, *p* < 0.0167). All pairwise comparisons were two-sided. *p < 0.0167; n.s. not significant. Source data are provided as a Source Data file.

Microglial activation is a hallmark of AD pathology^20^. Our analysis revealed robust upregulation of disease-associated microglia (DAM) genes, including *Tyrobp*, *Ctsd*, *Trem2*, *Cst7*, *Axl*, and *Itgax*, in *APOE3/4* mice relative to *APOE3/3* controls (Fig. 2A). In contrast, *APOE2/3* and *APOE2/4* mice exhibited DAM gene expression levels comparable to *APOE3/3*. *APOE* mRNA was also higher in *APOE3/4* mice (Supplementary Fig. 5A), and across animals, *APOE* expression was positively correlated with multiple DAM-associated genes (Supplementary Fig. 5B–N). Both *APOE* and DAM gene expression were positively associated with amyloid burden (Supplementary Fig. 5O–BB). Thus, animals with stronger DAM transcriptional signatures generally exhibited greater amyloid pathology, supporting a coordinated association among *APOE* gene expression, DAM activation, and amyloid burden.

Astrocytes also play important roles in neuroinflammation and synaptic regulation^21,22^. Two major astrocyte activation states have been described in neurodegenerative diseases: A1 neurotoxic astrocytes and A2 neuroprotective astrocytes^23^. However, we did not observe significant differences in the expression of A1 or A2 reactive astrocytic markers across *APOE* genotypes (Fig. 2A), despite the clear variations in amyloid burden and microglial activation. Similarly, although oligodendrocyte dysfunction and myelin integrity have been shown to influence Aβ accumulation^24^, gene expression patterns of oligodendrocyte-enriched transcripts did not differ significantly among genotypes (Fig. 2A). However, the direction of the shifts varied, with *APOE3/4* mice showing a trend toward decreased expression, whereas *APOE2/3* and *APOE2/4* mice trended toward increased expression of genes such as *Olig2* and *Mbp* compared to *APOE3/3* (Fig. 2A). Altogether, these findings suggest that, within this context, microglia represent the predominant glial responders to amyloid accumulation.

To validate these transcriptional findings, we performed immunohistochemical staining to examine the three major glial cell types. Consistent with the NanoString results, CD68, a lysosomal marker of activated microglia, was markedly upregulated in *APOE3/4* cortices compared with *APOE3/3* controls (Fig. 2B, C), confirming enhanced microglial activation. In contrast, *APOE2/3* and *APOE2/4* brains showed CD68 immunoreactivity comparable to *APOE3/3*. Quantification of Iba1-positive microglia revealed similar overall densities across genotypes (Supplementary Fig. 6A, B), indicating that *APOE* genotype primarily affects microglial activation state rather than cell number. Because efficient microglial clustering around plaques is essential for Aβ clearance and restricting local Aβ toxicity^25,26^, we next assessed plaque-microglia interactions by co-staining Iba1 with X-34. *APOE3/4* mice exhibited significantly reduced microglial clustering with plaque relative to *APOE3/3*, whereas *APOE2/3* and *APOE2/4* mice maintained plaque-associated microglial engagement comparable to *APOE3/3* (Fig. 2D, E).

Cortical GFAP-positive astrocyte coverage was numerically higher in *APOE3/4* mice, although the difference did not meet the adjusted significance threshold of *p* < 0.0167 (*p* = 0.03), whereas *APOE2/3* and *APOE2/4* mice displayed levels comparable to *APOE3/3* controls (Supplementary Fig. 6C, D). This indicates that, although A1 and A2 astrocyte subtype gene signatures were not significantly altered, a modest degree of amyloid-associated astrogliosis may be present.

Sex-stratified analyses demonstrated that the *APOE* genotype-dependent increase in CD68 immunoreactivity was generally preserved in both females and males (Supplementary Fig. 7A, B). Although the comparison between *APOE3/4* and *APOE3/3* mice no longer reached statistical significance in females after stratification, the direction and magnitude of the effect remained similar, consistent with reduced statistical power resulting from the smaller sample size within each sex. For plaque-associated microglia, females showed a pattern similar to that observed in the combined analysis, whereas the male cohort exhibited greater variability and fewer statistically significant genotype differences (Supplementary Fig. 7C, D). Given the reduced sample size following sex stratification, these subgroup analyses should be interpreted with caution and do not provide conclusive evidence for sex-dependent effects of *APOE* genotype.

**Fig. 3:**
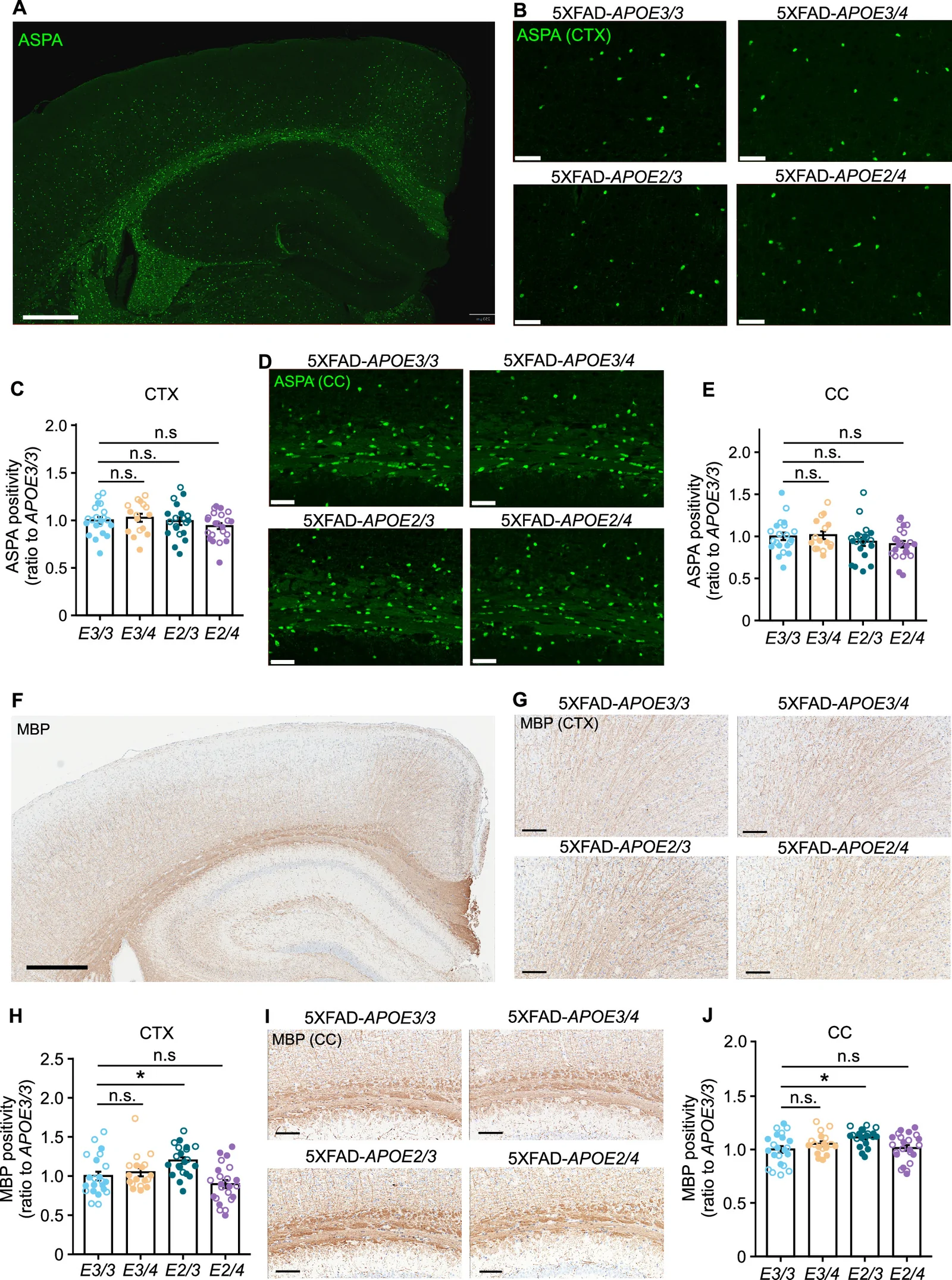
*APOE* genotype differentially affects MBP immunoreactivity. **A** Low-magnification image showing ASPA immunofluorescence across a coronal brain section. Scale bars, 500 µm. **B**, **C** Representative ASPA staining in the cortex (CTX) (**B**) and quantification (**C**) normalized to *APOE3/3* controls. Scale bars, 50 µm. *E3/3*, *n* = 21 mice; *E3/4*, *n* = 16 mice; *E2/3*, *n* = 20 mice; *E2/4*, *n* = 22 mice. **D**, **E** Representative ASPA staining in the corpus callosum (CC) (**D**) and quantification (**E**) normalized to *APOE3/3* controls. Scale bars, 50 µm. *E3/3*, *n* = 21 mice; *E3/4*, *n* = 16 mice; *E2/3*, *n* = 20 mice; *E2/4*, *n* = 22 mice. **F** Low-magnification image showing MBP immunofluorescence across a coronal brain section. Scale bars, 500 µm. **G**, **H** Representative MBP staining in the CTX region (**G**) and quantification (**H**) normalized to *APOE3/3* controls. Scale bars, 100 µm. *E3/3*, *n* = 22 mice; *E3/4*, *n* = 18 mice; *E2/3*, *n* = 21 mice; *E2/4*, *n* = 23 mice. (**I**, **J**) Representative MBP staining in the CC region (**I**) and quantification (**J**) normalized to *APOE3/3* controls. Scale bars, 100 µm. *E3/3*, *n* = 22 mice; *E3/4*, *n* = 18 mice; *E2/3*, *n* = 21 mice; *E2/4*, *n* = 23 mice. Data are presented as mean ± SEM. The data from female and male mice are labeled as solid and open circles, respectively. Statistical significance was determined using a linear model with sex included as a covariate, followed by Bonferroni adjustment for three pairwise comparisons versus *E3/3* (significance threshold, *p* < 0.0167). All pairwise comparisons were two-sided. *p < 0.0167; n.s. not significant. Source data are provided as a Source Data file.

To determine whether *APOE* genotype affected the oligodendrocyte lineage, we first quantified ASPA-positive mature oligodendrocytes. No significant genotype-dependent differences were observed in either the cortex (CTX) or corpus callosum (CC) (Fig. 3A–E). Likewise, the overall density of OLIG2-positive oligodendrocyte-lineage cells and BCAS1-positive newly formed or actively myelinating oligodendrocytes was similar across *APOE* genotypes in both brain regions (Supplementary Fig. 8A–H), indicating that *APOE* genotype does not substantially alter oligodendrocyte abundance or differentiation. We next examined myelin by assessing MBP immunoreactivity (Fig. 3F). *APOE3/4* mice exhibited MBP levels comparable to *APOE3/3* controls, whereas *APOE2/3* mice displayed significantly increased MBP staining in both the cortex (Fig. 3G, H) and corpus callosum (Fig. 3I, J). This increase was not observed in *APOE2/4* mice, whose MBP levels remained comparable to *APOE3/3* controls. Because oligodendrocyte lineage cell numbers were unchanged across genotypes, these isoform-dependent differences in MBP are more likely to reflect changes in myelin homeostasis or myelin organization rather than differences in oligodendrocyte abundance or maturation.

Sex-stratified analyses of MBP generally showed patterns similar to those observed in the combined cohort. While some genotype-dependent differences were more apparent in one sex than the other, the reduced sample size following stratification limited statistical power, and these analyses should not be interpreted as evidence for sex-dependent genotype effects (Supplementary Fig. 9A-D).

Together, these findings demonstrate that *APOE4* is associated with increased DAM gene expression, reduced plaque-associated microglial interactions, and greater Aβ accumulation. In *APOE2/4* mice, these phenotypes were less pronounced and more closely resembled those observed in *APOE3/3* and *APOE2/3* mice. *APOE2* also increased MBP immunoreactivity in *APOE2/3* mice, whereas this effect was not observed in *APOE2/4* mice, suggesting differential effects of *APOE* allele interactions on microglial activation and myelin biology.

### *APOE2* attenuates *APOE4*-induced proteomic remodeling linked to immune activation and synaptic dysfunction

To extend our findings beyond glial activation and gain a systems-level view of how *APOE* heterozygosity modifies brain biology, we next performed mass spectrometry-based proteomic profiling of cortical tissues to identify molecular pathways associated with the observed pathological differences.

**Fig. 4:**
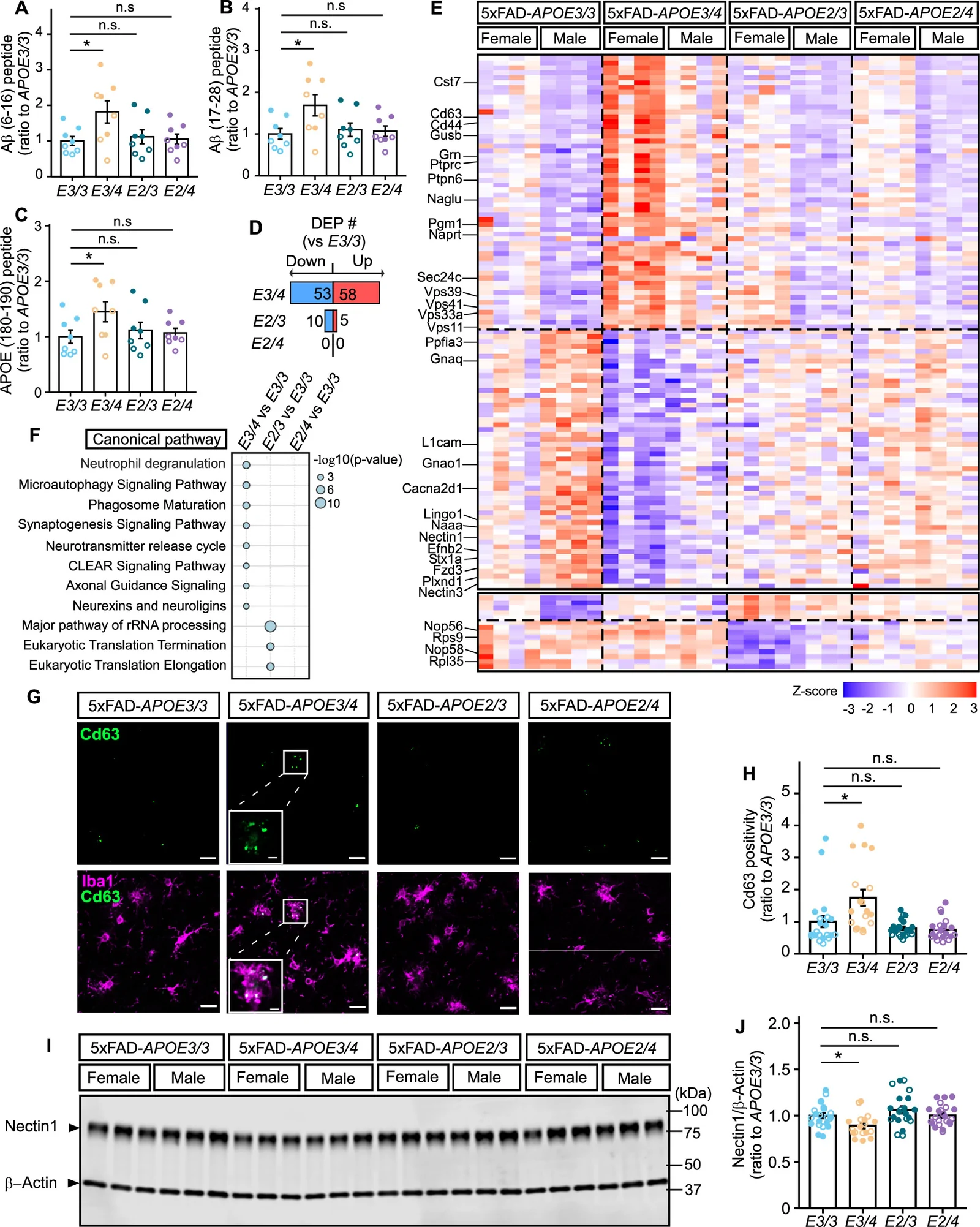
*APOE2* attenuates *APOE4*-induced proteomic remodeling linked to immune activation and synaptic dysfunction. **A**–**C** Quantification of Aβ6-16 peptide (**A**), Aβ17-28 peptide (**B**), and APOE peptide (**C**) abundance. *n* = 8 mice per group. **D** Numbers of differentially expressed proteins (DEPs) in cortical proteomes of 10-month-old 5xFAD-*APOE3/4*, *APOE2/3*, and *APOE2/4* mice compared with 5xFAD-*APOE3/3* controls. *n* = 8 mice per group. **E** Heatmap displaying normalized Z-scores of DEPs. **F** Canonical pathway enrichment of DEPs. **G**, **H** Representative Cd63 and Iba1 co-staining in the cortex (**G**) and quantification of Cd63 positivity (**H**) normalized to *APOE3/3* controls. Scale bars, 20 μm for the main image and 5 μm for the inset. *E3/3*, *n* = 22 mice; *E3/4*, *n* = 18 mice; *E2/3*, *n* = 21 mice; *E2/4*, *n* = 23 mice. **I**, **J** Western blotting showing Nectin1 protein levels in the cortical tissue lysates. *n* = 18–23 mice/group. *E3/3*, *n* = 22 mice; *E3/4*, *n* = 18 mice; *E2/3*, *n* = 21 mice; *E2/4*, *n* = 23 mice. Data in (**A**–**C**), (**H**) and (**J**) are presented as mean ± SEM. The data from female and male mice are labeled as solid and open circles, respectively. Statistical significance was determined using a linear model with sex included as a covariate, followed by Bonferroni adjustment for three pairwise comparisons versus *E3/3* (significance threshold, *p* < 0.0167). All pairwise comparisons were two-sided. *p < 0.0167; n.s. not significant. Source data are provided as a Source Data file.

Before analyzing the global proteomics dataset, we first examined the quantification of Aβ peptides to validate our histological and biochemical findings on amyloid burden. Consistent with prior results, levels of Aβ peptides, including Aβ6-16 and Aβ17-28, were significantly elevated in *APOE3/4* mice compared with *APOE3/3* controls, whereas *APOE2/3* and *APOE2/4* mice showed no significant changes (Fig. 4A, B). To determine whether these genotype-dependent differences in Aβ accumulation were accompanied by changes in APOE protein abundance, we next specifically quantified total human APOE using a peptide sequence common to all three isoforms (aa180-aa190). APOE protein levels followed a similar pattern: they were elevated in *APOE3/4* mice but remained comparable in *APOE2/3* and *APOE2/4* mice, relative to *APOE3/3* (Fig. 4C). This protein increase was consistent with the elevated *APOE* mRNA levels observed in *APOE3/4* mice (Supplementary Fig. 5A), suggesting that disease-associated induction of APOE expression may compensate for the lower intrinsic stability of APOE4 protein (Supplementary Fig. 1C). Together, these findings demonstrate that increased APOE levels in 5xFAD-*APOE3/4* mice parallels elevated Aβ accumulation.

Building on these analyses of Aβ and APOE, we next examined global protein expression changes to capture broader molecular pathways impacted by *APOE* heterozygosity. Among the 10,102 proteins detected (Supplementary Data 2), differentially expressed proteins (DEPs) were determined by comparing each heterozygous group to *APOE3/3* controls (Fig. 4D). Among all genotypes, *APOE3/4* mice exhibited the most extensive proteomic alterations, with 58 proteins upregulated and 53 downregulated relative to *APOE3/3*. In contrast, *APOE2/3* mice showed only minor changes (10 upregulated, 5 downregulated), and strikingly, no significant DEPs were detected in *APOE2/4* mice (Fig. 4D), indicating that the overall cortical proteomic profile of *APOE2/4* closely resembles that of *APOE3/3*. A heatmap visualization of DEPs revealed distinct proteomic signatures across *APOE* genotypes (Fig. 4E). In *APOE3/4* mice, upregulated proteins were enriched for markers of microglial activation and lysosomal processing (e.g., Cst7, Cd63, Cd44), as well as components of phagosomal and vesicular trafficking (e.g., Sec24c, Vps39, Vps41) (Fig. 4E). These alterations parallel the DAM signature identified by NanoString transcriptomic profiling and confirmed by immunohistochemistry (Fig. 2A, B). In contrast, several synaptic and axonal proteins, including L1cam, Nectin1, and Nectin3, were downregulated (Fig. 4E), consistent with increased neuritic dystrophy observed in *APOE3/4* mice (Fig. 1N, O). The DEP pathway enrichment analysis further supported these observations, revealing strong overrepresentation of biological processes related to neutrophil degranulation, microautophagy, phagosome maturation, synaptogenesis, neurotransmitter release, and axonal guidance in *APOE3/4* mice relative to *APOE3/3* controls (Fig. 4F). In contrast, neither *APOE2/3* nor *APOE2/4* mice exhibited significant alterations in these pathways. DEPs in *APOE2/3* relative to *APOE3/3* (e.g., Nop56, Rps9) were modest and enriched for rRNA processing and eukaryotic translation termination and elongation (Fig. 4D–F), reflecting a more stable, homeostatic proteomic landscape maintained by *APOE2*. To validate the major proteomic findings, we examined Cd63 by immunofluorescence staining. Cd63 immunoreactivity was enriched in microglia and increased in *APOE3/4* mice relative to *APOE3/3* controls, consistent with enhanced lysosomal activation in this genotype (Fig. 4G, H). We also assessed Nectin1 protein levels by Western blotting as a representative synaptic and neuronal protein. Consistent with the proteomic data, Nectin1 levels were reduced in *APOE3/4* mice compared with *APOE3/3* controls (Fig. 4I, J). Both Cd63 immunoreactivity and Nectin1 protein levels in *APOE2/3* and *APOE2/4* mice remained comparable to those in *APOE3/3* controls (Fig. 4G–J). Sex-stratified analyses revealed similar genotype-dependent patterns for both CD63 immunoreactivity and Nectin1 protein levels in females and males (Supplementary Fig. 10A-D), with reduced statistical significance in some comparisons following stratification because of the smaller sample size within each sex.

**Fig. 5:**
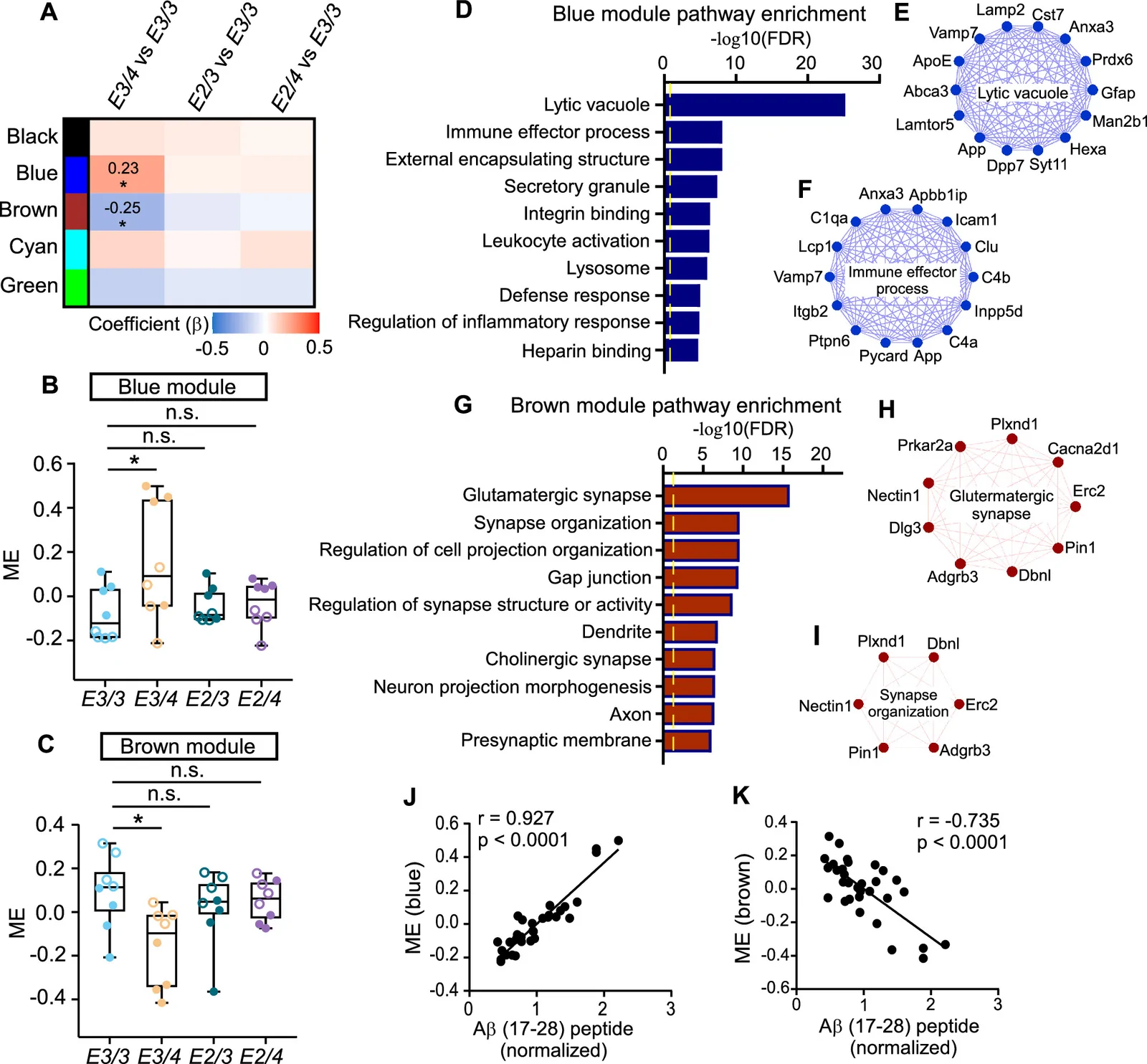
*APOE2* is associated with attenuated *APOE4*-related proteomic changes. **A** Module-trait relationships from WGCNA showing regression coefficients (β) for five selected modules in *APOE3/4*, *APOE2/3*, and *APOE2/4* mice relative to *APOE3/3* controls. The heatmap values represent the estimated β coefficients of module eigengenes (MEs) in each comparison. Warm colors (orange/red) indicate positive coefficients (higher ME expression relative *to APOE3/3*), whereas cool colors (blue) indicate negative coefficients (lower ME expression relative to *APOE3/3*). Asterisks denote statistically significant differences (*p < 0.0167). **B**, **C** Boxplots showing eigengene (ME) values for the blue (**B**) and brown (**C**) co-expression modules. The center line represents the median, the box bounds represent the 25th and 75th percentiles, and the whiskers extend to the minimum and maximum values; individual points represent individual mice. *n* = 8 mice/genotype. The data from female and male mice are labeled as solid and open circles, respectively. Statistical significance was determined using a linear model with sex included as a covariate, followed by Bonferroni adjustment for three pairwise comparisons versus *E3/3* (significance threshold, *p* < 0.0167). All pairwise comparisons were two-sided. *p < 0.0167; n.s., not significant. **D** Enriched GO terms in the blue module. **E**, **F** Network plots of the genes with the highest intramodular connectivity (hub genes) in the top pathways from the blue module. **G** Enriched GO terms in the brown module. **H**, **I** Network plots of the genes with the highest intramodular connectivity (hub genes) in the top pathways from the brown module. **J**, **K** Correlations between blue (**J**) or brown (**K**) module eigengene (ME) values and Aβ (17–28) peptide abundance (normalized to the cohort mean). Each dot represents one mouse (*n* = 32 mice in total; *n* = 8 per genotype). Pearson correlation coefficients (r) and corresponding two-sided *p* values are shown in each panel. Source data are provided as a Source Data file.

To identify co-regulated protein networks, we performed weighted gene co-expression network analysis (WGCNA) across all samples, revealing 19 modules (Supplementary Data 2). Consistent with the DEP analysis, the blue module, enriched for immune response proteins, was significantly upregulated in *APOE3/4* mice relative to *APOE3/3* controls (Fig. 5A, B). Functional enrichment of this module highlighted lysosomal and phagocytic processes, leukocyte activation, and immune effector pathways (Fig. 5D–F). Conversely, the brown module was downregulated (Fig. 5A, C) and was enriched for synaptic function-related pathways, including glutamatergic synapse function, synapse organization, and neuronal projection organization (Fig. 5G–I). Both modules remained largely unchanged in *APOE2/3* and *APOE2/4* mice (Fig. 5A–C). Principal component analysis (PCA) of individual module eigengenes revealed substantial overlap among *APOE* genotypes and sexes across most modules. A modest trend toward separation of *APOE3/4* female samples was observed in the blue and brown modules (Supplementary Fig. 11), the two modules most strongly associated with *APOE* genotype, although no distinct clustering was evident. Importantly, correlation analyses revealed that the blue immune module positively correlated with cortical Aβ peptide levels, whereas the brown synaptic module correlated negatively (Fig. 5J, K), linking these coordinated proteomic signatures to amyloid burden.

Together, these findings reveal that the presence of *APOE4* results in coordinated proteomic remodeling characterized by activation of immune and lysosomal programs and suppression of synaptic maintenance pathways, tightly coupled to amyloid pathology. In contrast, *APOE2*, either alone or in combination with *APOE4*, maintains molecular homeostasis, effectively attenuating *APOE4*-driven inflammatory and synaptic disruption.

### Plasma lipid alterations linked to *APOE* genotype and brain Aβ load

We next asked whether these *APOE*-genotype dependent effects extend beyond the brain to peripheral lipid metabolism, a core physiological function of APOE^27^. To address this, we performed both untargeted and targeted plasma lipidomic profiling in the same cohort to determine how *APOE* heterozygosity influences systemic lipid homeostasis in relation to amyloid pathology in the brain.

**Fig. 6:**
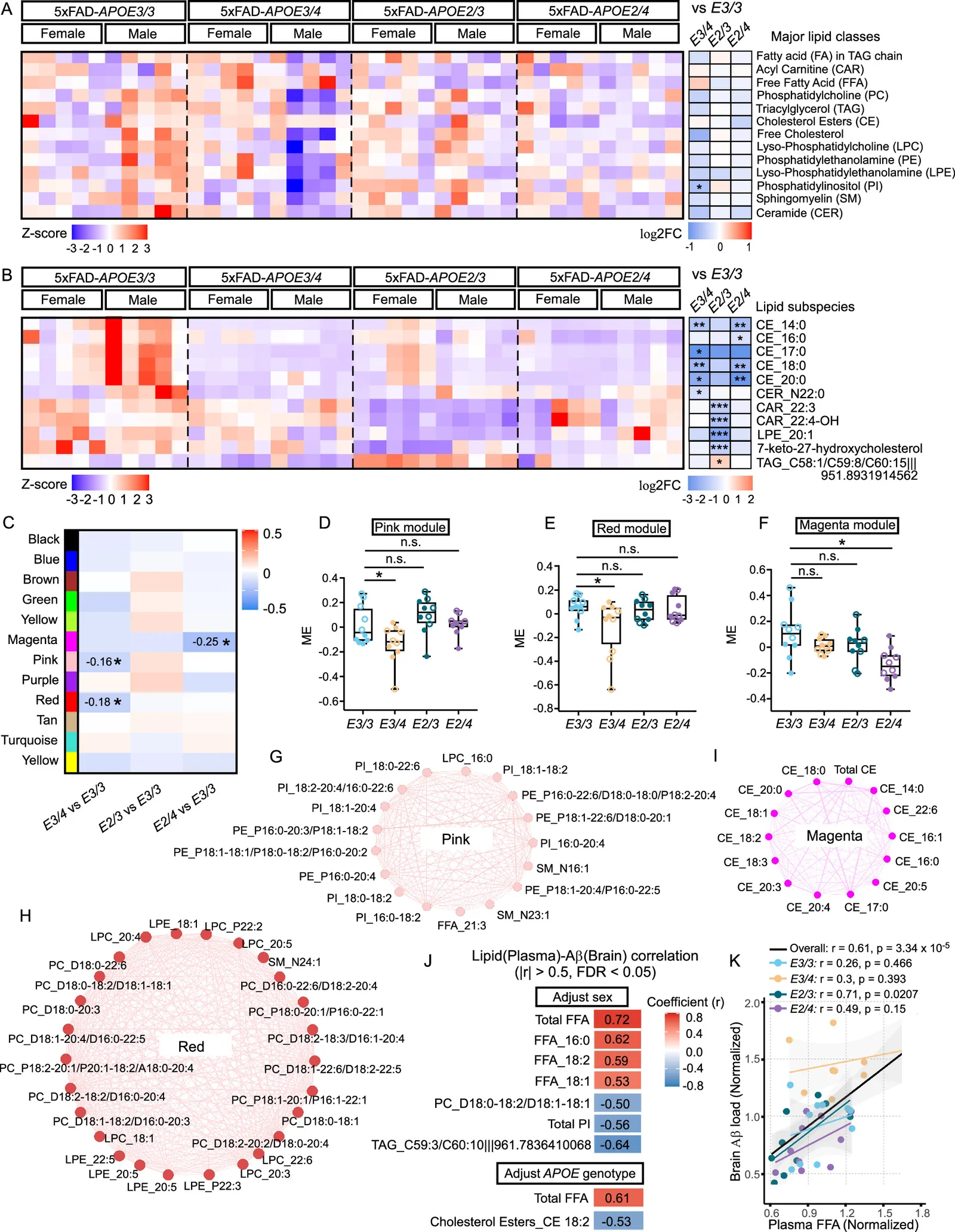
Plasma lipid alterations influenced by *APOE* genotype and their association with brain Aβ burden. **A** Heatmap showing normalized Z-scores of plasma major lipid classes across genotypes. right: log2 fold changes (log2FC) of the DE lipids relative to *APOE3/3*. Asterisks indicate significant changes after FDR correction. *FDR < 0.05. *n* = 10 mice/group. Sex was adjusted in the analysis. **B** Heatmap showing normalized Z-scores of plasma lipid subspecies across genotypes. right: log2FC of the DE lipids relative to *APOE3/3*. Asterisks indicate significant changes after FDR correction. *FDR < 0.05, **FDR < 0.01, ***FDR < 0.001. *n* = 10 mice/group. Sex was adjusted in the analysis. (**C**) Module-trait relationships from WGCNA showing regression coefficients (β) for each module in *APOE3/4*, *APOE2/3*, and *APOE2/4* mice relative to *APOE3/3* controls. The heatmap values represent the estimated β coefficients of module eigengenes (MEs) in each comparison. Warm colors (orange/red) indicate positive coefficients (higher ME expression relative *to APOE3/3*), whereas cool colors (blue) indicate negative coefficients (lower ME expression relative to *APOE3/3*). Asterisks denote statistically significant differences (*p < 0.0167). **D**–**F** Boxplots of module eigengene (ME) values for the pink, red, and magenta modules. The center line represents the median, the box bounds represent the 25th and 75th percentiles, and the whiskers extend to the minimum and maximum values; individual points represent individual mice. *n* = 10 mice/group. The data from female and male mice are labeled as solid and open circles, respectively. *p < 0.0167; n.s., not significant. **G**–**I** Network plots showing lipid members of the pink, red, and magenta modules, respectively. **J** Heatmap of correlation coefficients (r) between plasma lipid species and cortical Aβ burden after adjustment for either sex (top) or *APOE* genotype (bottom). Only lipid-Aβ associations with |r | > 0.5 and FDR < 0.05 are displayed. Red color indicates positive lipid-Aβ correlations, whereas blue indicates negative correlations. **K** Scatterplot showing a positive association between plasma total FFA levels measured by lipidomics and cortical Aβ load quantified by immunostaining; both variables are normalized to the cohort mean. Solid lines indicate linear regression fits, and shaded bands represent 95% confidence intervals. Each dot represents one mouse. Pearson’s correlation coefficients (r) and corresponding *p* values are shown for the overall cohort and for each *APOE* genotype group. Statistical significance in (**C**), **D**–**F** was determined using a linear model with sex included as a covariate, followed by Bonferroni adjustment for three pairwise comparisons versus *E3/3* (significance threshold, *p* < 0.0167). All pairwise comparisons were two-sided. *p < 0.0167; n.s. not significant. Source data are provided as a Source Data file.

Untargeted lipidomics identified 303 lipid subspecies spanning major lipid categories, including acyl carnitine (CAR), triacylglycerol (TAG), fatty acid (FA) in TAG chain, free fatty acid (FFA), cholesterol esters (CE), free cholesterol, phosphatidylcholines (PC), phosphatidylethanolamines (PE), lysophosphatidylcholines (LPC), lysophosphatidylethanolamines (LPE), phosphatidylinositols (PI), sphingomyelin (SM), and ceramide (CER) (Supplementary Data 3). We first examined whether *APOE* genotype affected lipid abundance at the major class level. Differentially expressed lipids (DELs) were determined by comparing each heterozygous group to *APOE3/3* controls (Fig. 6A). Overall lipid abundance was largely unchanged across genotypes; however, total PI was significantly decreased in *APOE3/4* mice compared with *APOE3/3* controls. No significant changes were detected in *APOE2/3* or *APOE2/4* mice.

To gain finer resolution, we next examined lipid subspecies in each class and additionally performed targeted measurement and quantification of eicosanoids and docosanoids, 4-hydroxynonenal (4-HNE) and related peroxidation products, as well as oxysterol (OS)-related lipids (Supplementary Data 3), which have been previously implicated in neurodegenerative disease^28–32^. Differential expression analysis at the subspecies level revealed that *APOE3/4* and *APOE2/4* mice exhibited a similar pattern of reduced cholesterol ester (CE) species compared with *APOE3/3*, suggesting that *APOE4* is associated with disruptions in plasma cholesterol ester metabolism and that *APOE2* cannot attenuate this effect in the heterozygous *APOE2/4* state (Fig. 6B). In contrast, *APOE2/3* mice displayed selective decreases in long-chain acyl-carnitines (CAR 22:3, CAR 22:4-OH), lysophosphatidylethanolamine (LPE 20:1), and the oxysterol 7-keto-27-hydroxycholesterol, potentially indicating that *APOE2* enhances β-oxidation and mitigates lipid peroxidation in the absence of *APOE4* (Fig. 6B). In addition, long-chain triacylglycerols (TAG C58:1/C59:8/C60:15III951.8931914562) were significantly elevated only in *APOE2/3* mice (Fig. 6B), potentially indicating an *APOE2*-associated shift in TAG metabolism or neutral lipid storage.

To further uncover coordinated lipid changes, we applied WGCNA to all these lipid subspecies and identified 12 co-regulated lipid modules (Fig. 6C). Compared with *APOE3/3* controls, the pink and red modules were significantly decreased in *APOE3/4* mice, whereas the magenta module was selectively reduced in *APOE2/4* mice (Fig. 6C–F). The pink module was enriched for PI and PE species (Fig. 6G), the red module comprised mainly PC, LPC, and LPE (Fig. 6H), and the magenta module consisted almost exclusively of cholesterol esters (CE) (Fig. 6I).

To determine whether these circulating lipid alterations relate to brain amyloid pathology, we next correlated plasma lipid levels with cortical Aβ burden quantified by immunostaining from the same animals. After adjusting for sex, free fatty acids (FFAs) showed a strong positive association with Aβ load, whereas total PI, along with two specific TAG and PC subspecies, correlated negatively (Fig. 6J). Notably, after adjustment for *APOE* genotype, FFAs remained significantly and positively correlated with Aβ deposition, whereas CE 18:2 showed a negative correlation (Fig. 6J, K). Together, these findings reveal *APOE* genotype-dependent perturbations in plasma lipid metabolism and identify FFAs as a genotype-independent peripheral correlate of brain Aβ accumulation.

Building on the plasma lipidomic findings, we next examined whether these peripheral lipid changes were accompanied by corresponding alterations in the brain. We therefore performed targeted lipidomic analysis of CEs and FFAs (Supplementary Data 4), the two lipid classes most prominently highlighted by the plasma dataset. Total brain CE and FFA levels did not differ significantly across *APOE* genotypes (Supplementary Fig. 12A, B). Analysis of individual lipid species revealed limited genotype-associated differences, including increased CE 20:4 in *APOE3/4* mice and increased FFA 18:0 and FFA 22:6 in *APOE2/4* mice relative to *APOE3/3* controls (Supplementary Fig. 12C). Notably, differing from the plasma findings, total brain CE levels were positively correlated with cortical Aβ burden, whereas total brain FFA levels showed no significant association (Supplementary Fig. 12D, E). Together, these findings suggest that brain and plasma lipid alterations may reflect distinct biological compartments. In particular, local CE accumulation appears to be more closely associated with cerebral amyloid pathology, whereas the plasma FFA association may represent a peripheral metabolic signature.

## Discussion

In this study, we generated littermate-controlled heterozygous *APOE* mice (*APOE2/3*, *APOE3/4*, and *APOE2/4*) together with *APOE3/3* controls and systematically characterized Aβ pathology, glial responses, brain proteomic profiles, and plasma lipid composition on the 5xFAD amyloid background. Our findings highlight three key insights: (1) the *APOE4* allele in *APOE3/4* mice significantly exacerbates amyloid deposition, promotes neuritic dystrophy, and impairs microglial engagement with plaques compared with *APOE3/3* mice. This confirms its pathogenic effects even in the heterozygous state with *APOE3*; (2) the *APOE2* allele in *APOE2/4* mice largely attenuates these *APOE4*-driven effects, maintaining amyloid burden, neurotoxicity, and microglial function at levels comparable to *APOE3/3* controls, while the increased MBP abundance observed in *APOE2/3* mice is absent in *APOE2/4* mice; and (3) plasma lipidomic analysis revealed genotype-dependent alterations, with *APOE3/4* and *APOE2/4* mice sharing partially overlapping lipid signatures relative to *APOE3/3*, and total FFAs emerging as a strong predictor of brain Aβ burden independent of *APOE* genotype.

Previous studies using homozygous *APOE* knock-in mice have been essential for defining isoform-specific effects of *APOE2*, *APOE3*, and *APOE4*, while viral *APOE2* delivery, *APOE2* gene therapy, and inducible *APOE4* to *APOE2* switching have demonstrated that increasing APOE2 expression or replacing *APOE4* with *APOE2* can ameliorate AD-related phenotypes^7,8,33,34^. However, these approaches do not directly address how two different endogenous *APOE* alleles interact when naturally co-expressed throughout life. Our heterozygous model addresses this complementary question. We found that endogenous *APOE2* significantly attenuated *APOE4*-associated amyloid pathology and related cellular and molecular phenotypes in *APOE2/4* mice, supporting the ability of *APOE2* to mitigate major *APOE4*-associated effects even when both alleles are present. Importantly, this influence was not uniform across all phenotypes: *APOE2/4* mice did not show the increased MBP abundance observed in *APOE2/3* mice and retained several peripheral lipid alterations shared with *APOE3/4* mice. Thus, our heterozygous model provides a physiologically relevant framework for defining how different *APOE* alleles interact in vivo and reveals allele-specific effects that cannot be readily captured by homozygous or single-isoform manipulation models.

The association of the APOE4 isoform with amyloid deposition has been well established in human studies, where *APOE4* carriers exhibit higher Aβ plaque burden and earlier onset of amyloid positivity compared with non-carriers^35–37^. Using homozygous human *APOE* knock-in mice, previous work demonstrated that *APOE4* promotes Aβ aggregation and impairs its clearance relative to *APOE3*^34,38,39^. Here, we extend these studies by examining heterozygous *APOE3/4* mice in a littermate-controlled design that eliminates background variability and allows direct assessment of single-allele effects. Our results demonstrate that even one copy of *APOE4*, in the presence of *APOE3*, is sufficient to exacerbate amyloid pathology compared with *APOE3/3* mice in the 5xFAD model, highlighting the potent pathogenic influence of *APOE4* in the heterozygous state.

Microglia form a critical interface between *APOE* genotype and amyloid pathology, mediating Aβ clearance and restricting local toxicity^25,40^. In our study, *APOE3/4* mice displayed upregulation of DAM genes at the bulk level, consistent with previous observations in *APOE4/4* mice^41^. However, despite this elevated activation signature, microglial clustering around plaques was markedly reduced in *APOE3/4* mice compared with *APOE3/3*, indicating a state of functional impairment. Consistent with these findings, our recent human study also revealed diminished plaque-associated microglial engagement in *APOE3/4* and *APOE4/4* cases^42^. Such paradoxical enhancement of activation but reduced functional plaque interaction may represent a transition toward an exhausted-like microglial state, recently described as terminally stressed inflammatory microglia (TIM) enriched in *APOE4* and aging contexts, characterized by heightened stress and pro-inflammatory responses accompanied by impaired phagocytic activity^43^. Together, these results suggest that *APOE4* promotes a maladaptive microglial phenotype that is highly activated yet functionally ineffective in constraining amyloid pathology.

In humans, the *APOE2/4* genotype confers an intermediate risk, higher than that of *APOE3/3* but lower than *APOE3/4* and *APOE4/4*^5,6^. However, its specific influence on amyloid pathology remains uncertain. Amyloid PET imaging results remain inconsistent, with some studies reporting attenuated or delayed amyloid deposition in *APOE2/4* individuals relative to *APOE3/4*^44,45^, whereas others have reported no significant difference between these genotypes^46^. These discrepancies are likely attributable to the limited size of *APOE2/4* cohorts, heterogeneity in disease stage at enrollment, and the influence of coexisting pathologies such as tau-related changes. Our study is the first to directly assess whether *APOE2* retains its protective effects in the presence of *APOE4* within an amyloid-driven context. Interestingly, *APOE2/4* mice exhibited amyloid plaque burden, fibrillar Aβ deposition, and leptomeningeal CAA levels comparable to *APOE3/3* littermates, indicating that *APOE2* effectively attenuates *APOE4*’s pathogenic influence. Plaques in *APOE2/4* mice were smaller and more compact, resembling those in *APOE2/3* animals, suggesting that *APOE2* promotes plaque compaction and limits diffusion of toxic Aβ species^47^. Correspondingly, microglia in *APOE2/4* mice maintained balanced activation and robust plaque clustering, similar to *APOE3/3* controls. Proteomic profiling revealed that *APOE2/4* cortices were nearly indistinguishable from *APOE3/3* at the molecular level, indicating preserved homeostasis and a lack of inflammatory remodeling. Notably, a recent inducible *APOE4* to *APOE2* allelic switching model showed that converting *APOE4* to *APOE2* in adult mice, particularly in astrocytes, reduces amyloid pathology and gliosis and improves cognitive outcomes on the 5xFAD background^7^. Together with our findings in *APOE2/4* mice, these results support the idea that APOE2 can attenuate APOE4-mediated amyloid pathology and highlight APOE2 isoform conversion as a promising Aβ-targeted therapeutic strategy. In contrast, peripheral lipidomics revealed that *APOE2/4* mice exhibited a partial overlap with *APOE3/4* animals, including reductions in several cholesterol ester subspecies, compared to *APOE3/3*. This similarity suggests that this effect might be related to the *APOE4* allele, although the contribution of circulating cholesterol levels to AD risk remains unclear.

In our study, *APOE2/3* mice did not show additional reductions in amyloid burden relative to *APOE3/3* animals. Even in homozygous *APOE2/2* mice, prior reports have been inconsistent regarding further reductions in amyloid pathology compared with *APOE3/3*, reflecting ongoing debate^34,48,49^. For instance, in the 5xFAD model, *APOE2/2* mice displayed amyloid loads similar to *APOE3/3* at six months of age^48^, whereas modest reductions were observed in 18-month-old *APOE2/2* PDAPP mice compared with *APOE3/3*^49^, however, no significant differences were detected in another study using the same models^34^. Future investigations using more physiological models, such as human APP knock-in mice assessed across different time points, may better clarify these genotype effects during disease progression. Moreover, as our current work focused primarily on amyloid pathology, it remains possible that *APOE2/3* exerts stronger protection in the context of tauopathy or combined amyloid-tau pathology. Notably, we observed enhanced myelin maintenance in *APOE2/3* mice compared with *APOE3/3*, suggesting that *APOE2*’s protective effects may act in part through preservation of myelin. This finding aligns with our recent observations in human brain studies, where *APOE2/3* cases displayed preserved myelin even at advanced AD stages relative to *APOE3/3*^42^. The absence of this enhancement in *APOE2/4* mice raises the possibility that *APOE4* limits *APOE2*-mediated myelin support, aligning with the recent evidence that *APOE4* exacerbates white matter impairment^50^.

The relationship between plaque morphology and toxicity is complex. *APOE4* has been associated with altered plaque morphology, greater fibrillar amyloid deposition, and enhanced plaque-associated microglial reactivity, supporting the view that increased fibrillar plaque pathology can accompany a more severe disease phenotype^51^. In contrast, impaired microglial barrier formation has been shown to reduce plaque compaction and increase the exposure of surrounding neurites to fibrillar Aβ, resulting in greater neuritic dystrophy^47^. Pathological consequences likely depend on total amyloid burden, plaque organization, microglial containment, and the extent of peri-plaque neuritic injury. In the present study, *APOE2*-containing genotypes showed a greater relative proportion of compact plaques but lower overall fibrillar amyloid burden than *APOE3/4* mice. These findings indicate that *APOE* genotype influences both plaque architecture and amyloid load, while the functional significance of the more compact plaque morphology observed in *APOE2*-containing genotypes remains to be explored.

In this study, increased leptomeningeal CAA was observed in *APOE3/4* mice relative to *APOE3/3* controls, whereas a corresponding increase was not detected in *APOE2/4* mice under the conditions examined. However, human studies have linked *APOE2* to greater CAA severity in some cohorts^52^, and to CAA-related vasculopathic changes and hemorrhagic complications^53,54^. This apparent discrepancy may reflect species-specific differences, limitations of the rapidly amyloid-depositing 5xFAD model, assessment at a single age, our focus on leptomeningeal CAA rather than vascular amyloid across different vessel types and brain regions, and the distinction between vascular amyloid burden and vessel-wall injury. Future studies using complementary models and broader assessments of CAA-associated vascular pathology will be needed to clarify how *APOE2* influences cerebrovascular amyloid and vessel-wall injury.

The enrichment of translational and ribosomal pathways in *APOE2/3* mice raises the possibility that *APOE2* influences protein synthesis or proteostatic regulation in the brain. Such changes could affect cellular stress responses, synaptic maintenance, or glial adaptation and thereby contribute to the comparatively mild phenotype observed in *APOE2/3* mice compared to *APOE3/3*. However, because these findings were derived from bulk cortical proteomics and showed modest effect sizes, their cellular origin and functional significance remain uncertain. Future studies using cell type-specific transcriptomic or proteomic approaches, together with direct measurements of translational activity, will be needed to determine whether altered protein synthesis represents a meaningful *APOE2*-associated protective mechanism.

At the systemic level, *APOE2/3* mice exhibited reduced long-chain acylcarnitines and oxidized 7-keto-27-hydroxycholesterol, lipid species known to accumulate with aging and AD^32,55,56^, suggesting a potential peripheral signature of *APOE2*-associated protection. Additionally, our plasma lipidomic analyses revealed that specific peripheral lipid alterations correlate with brain Aβ burden. Among these, FFAs showed a strong positive association with cortical Aβ load, independent of *APOE* genotype. Previous in vitro studies have demonstrated that FFAs can stimulate the assembly of Aβ filaments^57^. Therefore, elevated FFAs may represent a general metabolic signature associated with amyloid pathology, although the precise mechanisms linking it to brain Aβ accumulation remain to be elucidated. In contrast, PI levels were significantly reduced in *APOE3/4* mice relative to *APOE3/3* controls and negatively correlated with brain Aβ burden, suggesting that PI depletion could serve as a sensitive indicator of *APOE4*-associated lipid dysregulation and brain Aβ accumulation. While the mechanistic basis for this association is unclear, these findings collectively highlight the interplay between peripheral lipid metabolism and brain amyloid deposition. The divergent relationships observed in plasma and brain lipid profiles highlight the compartment-specific nature of lipid dysregulation in this model. Although plasma FFAs were positively associated with brain Aβ burden, brain FFAs showed no corresponding relationship. Conversely, brain CE levels were positively associated with amyloid burden despite the absence of significant genotype-dependent differences in total brain CE. These findings suggest that circulating lipid signatures may reflect systemic processes, such as hepatic metabolism or lipoprotein remodeling, whereas brain CE accumulation may be more closely linked to local amyloid pathology. Future studies incorporating parallel brain and liver lipidomics, lipoprotein fractionation, and metabolic flux analyses will be needed to define the biological sources, functional significance, and mechanistic relationships underlying these compartment-specific lipid alterations.

Several questions remain to be addressed. First, the relative contributions of cell-autonomous and non-cell-autonomous APOE signaling remain unresolved. Although our data demonstrate robust *APOE* genotype-dependent alterations across multiple molecular and pathological phenotypes, the respective roles of microglial, astrocytic, and neuronal *APOE* require further investigation. Combining cell type-specific APOE manipulation with emerging single-cell and spatial proteomic approaches will help define the cellular mechanisms underlying these allele-specific effects. Second, although *APOE2/4* mice exhibited reduced amyloid pathology relative to *APOE3/4* mice, our study does not distinguish whether this reflects protective functions conferred by *APOE2*, reduced *APOE4*-associated activity, or both. Future studies incorporating *APOE*-null alleles^58,59^ together with cell type-specific *APOE* manipulation will help resolve these possibilities and define how *APOE* allele interactions influence AD pathogenesis. Third, the present study was not specifically powered to detect genotype-by-sex interactions. While several *APOE4*-associated phenotypes appeared more pronounced in females, sex-stratified analyses should be interpreted cautiously because of the reduced sample size within each subgroup. Larger sex-balanced cohorts will be important for determining how sex hormones, immune responses, and lipid metabolism interact with *APOE* genotype during disease progression. In addition, although littermate-controlled breeding minimized genetic background effects, the parent-of-origin inheritance of the *APOE* alleles and the 5xFAD transgene was not specifically controlled in this study. Future studies using reciprocal breeding strategies will help determine whether parent-of-origin effects contribute to variability in *APOE*-associated phenotypes^60,61^. Finally, our study focused on molecular and pathological changes in the 5xFAD amyloid model. Extending these findings to behavioral and cerebrovascular outcomes, validating the association between circulating free fatty acids and brain amyloid burden in independent and longitudinal cohorts, and examining additional models incorporating tau pathology will further establish the broader biological and translational significance of *APOE* allele interactions.

In summary, our study provides in vivo evidence that the protective *APOE2* allele can attenuate the deleterious effects of *APOE4* across multiple biological levels, including amyloid deposition, microglial activation and responses, and molecular proteomic profiles in the *APOE2/4* genotype. These findings advance the understanding of *APOE* allele interplay and suggest that harnessing *APOE2*-like mechanisms may offer a viable therapeutic strategy to restrict amyloid development and modulate AD risk and progression even in the presence of *APOE4*.

## Methods

### Animals

All animal procedures were approved by the Mayo Clinic Institutional Animal Care and Use Committee (IACUC) under protocol number A00003820-18-R24, and performed in accordance with the National Institutes of Health Guidelines for the Care and Use of Laboratory Animals. Animals were housed under controlled conditions of temperature and lighting and given free access to food and water. 5xFAD amyloid model mice were from The Jackson Laboratory (stock # 34848). The homozygous *APOE* knock-in mice (*APOE2/2*, *APOE3/3*, *APOE4/4*) were obtained from Dr. David M. Holtzman’s lab^10^. Heterozygous *APOE* mice (*APOE2/3*, *APOE3/4*, and *APOE2/4)* and *APOE3/3* control mice were generated through a stepwise cross of homozygous human *APOE* knock-in mice as described in Fig. 1A.

### Tissue preparation

At 10 months of age, heterozygous *APOE* mice (*APOE2/3*, *APOE3/4*, and *APOE2/4*) and *APOE3/3* controls were fasted for 14 h prior to tissue collection. Mice were deeply anesthetized with isoflurane, and whole blood was collected via cardiac puncture into EDTA-coated tubes. Plasma was obtained by centrifugation at 1000 × *g* for 10 min at 4 °C and stored at −80 °C until lipidomic analysis. Following blood collection, mice were transcardially perfused with PBS. Brains were removed and bisected along the midline. One hemisphere was drop-fixed in 10% neutral-buffered formalin (Fisher Scientific, Waltham, MA) overnight at 4 °C for histological analyses. The contralateral hemisphere was dissected to isolate the cortical regions, which were snap-frozen in liquid nitrogen and stored at −80 °C until use.

For biochemical analyses, frozen cortices were pulverized under liquid nitrogen. Approximately 20 mg of cortical powder per sample was subjected to sequential three-step protein extraction. First, tissue was homogenized and lysed in Tris-buffered saline (TBS) containing protease and phosphatase inhibitors, followed by ultracentrifugation at 100,000 × *g* for 60 min at 4 °C; the resulting supernatant was collected as the TBS-soluble fraction. The pellet was then resuspended in TBS containing 1% Triton X-100 (TBSX) with inhibitors and ultracentrifuged again under identical conditions; the supernatant was collected as the TBSX-soluble fraction. The remaining pellet was resuspended and sonicated in 5 M guanidine hydrochloride (GND; Sigma-Aldrich), followed by centrifugation as above, and the supernatant was collected as the GND-soluble fraction. All fractions were stored at −80 °C until analysis by Western blotting or ELISA. The remaining pulverized cortical powders were aliquoted and stored at −80 °C for subsequent RNA extraction and proteomic analyses.

### Immunohistochemistry

Formalin-fixed hemispheres were embedded in paraffin, sectioned coronally at 5 µm, and mounted on glass slides. Sections were deparaffinized in xylene, rehydrated through graded alcohols, and subjected to antigen retrieval by steaming in deionized water for 30 min. Endogenous peroxidase activity was quenched with 0.03% hydrogen peroxide. Immunostaining was performed on the Autostainer 480S System (Thermo Scientific). Slides were then dehydrated, coverslipped, and scanned with an Aperio Slide Scanner (Aperio, Vista, CA) as previously described^62^. The following primary antibodies were used: anti-Aβ (33.1.1, in-house, 1:5000), anti-GFAP (Cat# Pu020-UP, BioGenex, 1:2500), anti-Iba1 (Cat# 019-19741, Wako, 1:3000), anti-CD68 (Cat# ab125212, Abcam, 1:400), and anti-MBP (Cat# MAB42282, R&D Systems, 1:10,000). Image quantification was performed in Aperio ImageScope v12.4.6.5003 (Leica Biosystems, Germany). The frontal cortex, hippocampus and corpus callosum were manually delineated as the region of interest (ROI), and a unified threshold and analysis algorithm was consistently applied across all samples for each staining. All staining, imaging, and quantification were conducted by investigators blinded to sample identity and demographic information.

### Immunofluorescence

Paraffin-embedded brain sections were used for immunofluorescent staining. After deparaffinization and rehydration, antigen retrieval was performed by steaming slides in Tris-EDTA Buffer (#AB93684, Abcam) for 30 min. When needed, X-34 staining was performed after antigen retrieval by incubating the slides in 10 μM X-34 working solution (40% ethanol, 60% PBS, pH=10) for 20 min at room temperature. Subsequently, the slides were washed three times with wash solution (40% ethanol, 60% PBS) and three times with PBS and then blocked with serum-free protein block (Cat# X0909, Agilent Technologies) and incubated with primary antibodies at room temperature for 2 h. The following primary antibodies were used: anti-APP-NT (Cat# MAB348, Milipore, 1:300); anti-Iba1 (Cat# 17198S, Cell Signaling Technology, 1:300); and anti-ASPA (Cat# ABN1698, Milipore, 1:300); anti-CD63 (Cat# 32604-1-AP, Proteintech, 1:300); anti-ZO-1 (Cat# 33-9100, Invitrogen, 1:300); anti-Albumin (Cat# A90-134, Bethyl Laboratories, 1:300); anti-CD13 (Cat# AF2335, R&D Systems, 1:300); anti-Glut1 (Cat# ab15309, Abcam, 1:500); anti-Olig2 (Cat# ab109186, Abcam, 1:300); and anti-BCAS1 (Cat# 445 003, Synaptic Systems, 1:300). After primary antibody incubation, the slides were incubated with Alexa-dye conjugated secondary antibodies targeting the respective host species (1:500) for 2 h at room temperature.

To quantify ASPA, CD63, ZO-1, CD13, Glut1, Olig2 and BCAS1 coverage, slides were scanned by Phenoimager HT microscopes at 20x magnification with autofocus performed under identical settings. The frontal cortex and corpus callosum were manually traced and saved as regions of interest (ROIs) using QuPath or ImageJ. A unified intensity threshold was applied across all samples within each staining. Pixels exceeding this threshold were quantified to determine the percentage of positive immunoreactive area using the particle analysis plugin. For quantification of X-34 associated APP-NT or X-34 associated Iba1 signal, slides were imaged using a confocal microscope at 20X magnification (LSM800, Zeiss, Germany). Three cortical ROIs per section were acquired, and X-34 associated APP-NT or Iba1 signal was quantified within a 15-µm radius surrounding each X-34 positive plaque using customized Python scripts.

### RNA isolation and NanoString nCounter glial profiling

20–25 mg of the pulverized cortical tissue was taken for the total RNA extraction. Total RNA was isolated using the RNeasy Mini kit (74104, Qiagen, Germany), and a total of 200 ng RNA per sample was subjected to gene expression profiling using the nCounter^®^ Glial Profiling panel from NanoString (NanoString Technologies, USA). Data were analysed using the nSolver Analysis Software, version 4.0. The background of all the samples was subtracted using a threshold with a defined value of 20. Thereafter, gene expression levels in each sample were normalized against the geometric mean of positive controls and housekeeping genes provided by the panel.

### qPCR analysis

1 µg total RNA was reverse transcribed using the iScript™ Reverse Transcription Supermix (Cat# 1708891, Bio-Rad). The resulting cDNA was combined with gene-specific primers and SYBR Green Supermix (Cat# 172-5274, Bio-Rad) in a 10 µL reaction volume. All samples were run in technical duplicates on a QuantStudio 7 Flex Real-Time PCR System (Thermo Fisher Scientific). Relative transcript levels were calculated using the 2^−ΔΔCT^ method with actin as the normalization control. Primer sequences were as follows (5′−3′): human *APOE*: TGTCTGAGCAGGTGCAGGAG (forward) and TCCAGTTCCGATTTGTAGG (reverse); murine actin: AGTGTGACGTTGACATCCGTA (forward) and GCCAGAGCAGTAATCTCCTTC (reverse)^63^.

### Western blotting

Equal amounts of protein from the TBS or TBSX fractions of homogenized tissue lysates or isolated microglia were resolved by SDS-PAGE and transferred to polyvinylidene difluoride membranes. After blocking with 5% nonfat milk, the membrane was incubated with primary antibodies at 4 °C overnight and horseradish peroxidase (HRP)-conjugated secondary antibodies at room temperature for 2 h. Chemiluminescence was detected using ECL Substrate (Pierce). Immunoblots were visualized using the Bio-Rad ChemiDoc MP imaging system. The following primary antibodies were used: anti-human APOE (Cat# K74180B, Meridian Life Science, 1:5,000), anti-β-actin (Cat# A2228, Sigma-Aldrich, 1:3,000), anti-Albumin (Cat# 4929, Cell Signaling Technology, 1:1,000) and anti-Nectin1 (Cat# ab323704, Abcam, 1:1000).

### Aβ ELISA

The levels of Aβ40 and Aβ42 in TBS, TBSX, and GND fractions of brain lysates were determined by ELISA method as previously described^64^. End-specific monoclonal antibodies were used for capture (13.1.1 for Aβ40 and 2.1.3 for Aβ42; produced in-house at Mayo Clinic), and an HRP-conjugated monoclonal antibody (Ab5; produced in-house at Mayo Clinic) was used for detection.

### Mass spectrometry-based proteomics

Deep proteome profiling was achieved through tandem mass tag (TMT) labeling integrated with two-dimensional liquid chromatography and tandem mass spectrometry (TMT-LC/LC-MS/MS), following previously established protocols^65,66^.

For protein extraction and digestion, mouse brain tissue samples underwent homogenization in a lysis buffer comprised of 50 mM HEPES (pH 8.5), 8 M urea, and 0.5% sodium deoxycholate. Protein concentration was quantified via BCA assay and confirmed by Coomassie-stained gel electrophoresis^67^. 0.1 mg of protein per TMT channel was digested with Lys-C (1:100 enzyme-to-protein ratio, w/w) for 2 h at 21 °C, followed by overnight trypsin digestion (1:50 w/w) at 21 °C. Cysteine residues were reduced with DTT and alkylated with iodoacetamide, following the established protocol^66^. Protein digestion was terminated by adding trifluoroacetic acid to a final concentration of 1% (v/v). The digested peptides were desalted using Ultra MicroSpin Column (Harvard Apparatus)^68^.

The desalted peptides were labeled by individual TMTpro reagents (18-plex) with protein to TMT ratio of 1.5:1. For each batch, an equal amount of labeled samples was pooled and desalted with a Sep-Pak C18 Cartridge (Waters). The pooled peptides for each batch were separated using basic pH reverse-phase chromatography with an ACQUITY UPLC BEH column (3.0 mm × 15 cm, 1.7 μm particles; Waters). 40 concatenated fractions for each batch were collected and dried for LC-MS/MS analysis^68^.

Each fraction was analyzed by acidic pH LC-MS/MS on a 75 μm × 20 cm column containing 1.9 μm C18 particles, coupled with Orbitrap Exploris 480 or Q Exactive HF Orbitrap instruments. Each fraction was analyzed by 1 h gradient (buffer A: 0.2% formic acid, 3% DMSO; buffer B: buffer A plus 65% acetonitrile). Survey scans captured ions between *m/z* 460–1600 at 60,000 resolution, with AGC targets set to 3 × 10^6^ and 50 ms maximum injection time. The 20 data-dependent MS2 scans using the following configuration: 120 *m/z* fixed first mass, 60,000 resolution, 1 × 10^6^ AGC target, maximum injection time of 110 ms, HCD fragmentation at 32–35% normalized collision energy, 1.0 *m/z* isolation width with 0.2 *m/z* offset, and 10-second dynamic exclusion window^66^.

Raw data files were searched against protein sequence databases using JUMP software (version 1.13.4)^69^, which integrates pattern-matching and de novo sequencing algorithms to maximize identification sensitivity and accuracy. A target-decoy search strategy enabled FDR estimation^70^. The database merged Swiss-Prot, TrEMBL, and UCSC protein sequences, encompassing 59,423 mouse entries. Database searches incorporated the following parameters: mass measurement accuracy of ±10 ppm for both precursor and fragment ions, requirement for full tryptic specificity, fixed modifications including TMT labeling ( + 304.20715 Da on peptide N-termini and lysine) and carbamidomethylation ( + 57.02146 Da on cysteine), variable modification for oxidation ( + 15.99492 Da on methionine), two maximal missed cleavages, and a maximum of three modifications per peptide. The resulting peptide-spectrum matches (PSMs) were filtered based on match quality scores and mass measurement precision to achieve protein identification FDR under 1%. Protein quantification was performed by integrating TMT reporter ion intensities from all PSMs assigned to each protein using established methods^71^.

To specifically track APOE and Aβ peptides, the following peptides were extracted from the global database search results: APOE common peptide present in all isoforms (aa180-190, R.LGPLVEQGR.V), Aβ6-16 (R.HDSGYEVHHQK.L), and Aβ17-28 (K.LVFFAEDVGSNK.G). These peptides were identified during the standard proteome-wide database search based on their unique sequences. For the peptide quantification, TMT reporter ion intensities were extracted from all high-quality PSMs corresponding to these peptides, and peptide-level quantification was performed following the same protocol used for protein quantification^71^.

### Lipidomics

A total of 100 µL of plasma was used for lipidomic analysis of major lipid categories, including acyl carnitine (CAR), triacylglycerol (TAG), fatty acid (FA) in TAG chain, free fatty acid (FFA), cholesterol esters (CE), free cholesterol, phosphatidylcholines (PC), phosphatidylethanolamines (PE), lysophosphatidylcholines (LPC), lysophosphatidylethanolamines (LPE), phosphatidylinositols (PI), sphingomyelin (SM), and ceramide (CER), as well as eicosanoids and docosanoids, 4-hydroxynonenal (4-HNE) and related lipid peroxidation products, and oxysterol-related lipids. Plasma lipids were extracted from individual samples using a modified Bligh and Dyer protocol in the presence of internal standards, as previously described^72,73^. Briefly, a premixed internal standard solution encompassing multiple lipid classes was added to each plasma sample. Samples were then subjected to a modified Bligh-Dyer liquid-liquid extraction. The chloroform phase was collected, evaporated under a nitrogen stream, and the resulting lipid extract was reconstituted in chloroform/methanol (1:1, v/v) at a volume of 2 µL per µL of starting plasma. Reconstituted extracts were capped and stored at −20 °C until mass spectrometric analysis, as previously described^74^.

Lipid species were analyzed using multidimensional mass spectrometry-based shotgun lipidomic analysis^75^. A triple-quadrupole mass spectrometer (Thermo TSQ Quantiva, San Jose, CA, USA), equipped with an automated nanospray ion source (TriVersaNanoMate, Advion Bioscience Ltd., Ithaca, NY, USA) and Xcalibur system software, was used in the study^76^. Prior to direct infusion into the mass spectrometer through the NanoMate device, each extract was further diluted with chloroform/methanol/isopropanol (1:2:4, v/v/v) to achieve a final concentration of <50 pmol total lipid/µL.

Identification and quantification of lipid species were performed using an automated software program^74,77^. Data processing, including ion peak selection, baseline correction, data transfer, peak intensity comparison and quantitation, was performed as previously described^74^. Final lipid abundances were normalized to plasma volume and expressed as pmol lipid/mL plasma.

TAG-associated fatty acyl chains were identified by positive-ion electrospray ionization tandem mass spectrometry using neutral-loss scanning. Specific fatty acyl chains were assigned based on the neutral loss of the corresponding fatty acid from an intact TAG parent ion. Thus, fatty acids detected through this parent ion–neutral loss relationship represent esterified fatty acyl chains within TAG rather than free fatty acids^78^. Free fatty acids were quantified separately as an independent lipid class.

For brain targeted lipid analysis, 20 mg of pulverized cortical tissue from each sample was used for the measurement of CEs and FFAs. Lipid extraction and derivatization were performed using the same procedures as those used for the plasma lipid analyses, with adjustments to the internal standard concentrations and mass spectrometry conditions appropriate for brain tissue. Final lipid abundances were normalized to tissue weight.

### Proteomic data analysis

Raw peptide abundances from proteomics were quantile-normalized and log2-transformed. Samples Q4292 and Q4604 were removed from downstream analyses as outliers based on Principle Component Analysis (PCA). Differential peptides analyses were performed by Partek Genomics Suite (Partek Inc., St. Louis, MO) using quantile-normalized and log2-transformed peptide abundance values. Peptide abundances between each pair of *APOE* genotypes were compared using Analyses of Variance models (ANOVA) while adjusting for sex. The Benjamini-Hochberg (BH) procedure was applied to adjust for multiple testing. Differentially measured peptides (DMPs) were defined by thresholds of BH-adjusted *p* value < 0.05. DMPs belonging to the same proteins were merged for pathway analyses using Ingenuity Pathway Analysis (QIAGEN Inc., https://digitalinsights.qiagen.com/products/ingenuity-pathway-analysis)^79^.

Weighted correlation network analysis (WGCNA)^80^ was performed using normalized proteomics. Based on the relationship between power and scale independence, the power of 5 was chosen to build scale-free topology using signed hybrid network. The minimum modules size was set as 60, and merged modules whose correlation coefficients were greater than 0.6 (mergeCutHeight = 0.4). Each module was summarized by the first principal component of the scaled module expression profiles, termed module eigengene (ME). For each module, the module membership (MM) was defined as the correlation between peptide abundance values and ME. Intramodular hub peptides are peptides with the highest connectivity to other peptides within a given module, and were selected based on the *p* values of MM. For *APOE* genotype associations, module eigengenes were analyzed using pairwise linear regression models comparing each genotype group with *APOE3/*3 as the reference group, with sex included as a covariate. For each pairwise comparison, module eigengenes were evaluated using a binary genotype indicator representing the two genotype groups being compared. Modules significantly correlated with *APOE* genotype were visualized using VisANT^81^ and annotated using R package anRichment.

### NanoString data analysis

NanoString mouse glial-panel data from 48 samples were analyzed using the limma pipeline in R to identify *APOE* genotype-dependent transcriptional changes. Normalized gene expression matrices were imported, and metadata were curated to recode *APOE* genotypes as *E2/3*, *E2/4*, *E3/3*, and *E3/4*. A linear model was constructed using the formula ~0 + *APOE* Genotype + Sex, where genotype effects were estimated while adjusting for sex as a covariate. Bayes moderation was applied using eBayes. Pairwise contrasts were defined for all *APOE* genotype comparisons. Differentially expressed genes (DEGs) were extracted for each contrast using moderated *t*-statistics, and *p* values were adjusted by the Benjamini-Hochberg method to control the false discovery rate (FDR < 0.05). DEGs were ranked by log2FC and significance. To visualize expression patterns, z-score-normalized expression values of selected genes were plotted as box-and-dot plots using *ggplot2*, while corresponding log2FC values were displayed alongside to highlight the direction and magnitude of differential expression.

### Lipidomic data analysis

Plasma lipidomic profiles were analyzed using the limma framework in R to assess *APOE* genotype-dependent lipid changes and their relationship with Aβ pathology. Lipid subspecies and major class data were imported, with sample metadata defining genotypes (*E2/3*, *E2/4*, *E3/3*, *E3/4*) and sex. Lipid abundance was log₂-transformed for linear regression to fit ~0 + Genotype + Sex, and empirical Bayes moderation (eBayes) was applied. Pairwise contrasts among genotypes were tested, and significance was determined by the Benjamini-Hochberg FDR method (FDR < 0.05). To visualize expression patterns, z-score-normalized expression values of selected genes were plotted as box-and-dot plots using *ggplot2*, while corresponding log2FC values were displayed alongside to highlight the direction and magnitude of differential expression. Pearson correlation analyses were then performed between lipid levels and Aβ measures. Correlation coefficients and adjusted *p* values were summarized, and significant associations were visualized using scatter plots and heatmaps.

WGCNA^80^ was performed using normalized lipid values. Based on the relationship between power and scale independence, the power of 5 was chosen to build scale-free topology using signed hybrid network. We set the minimum module size as 10, and merged modules whose correlation coefficients were greater than 0.85 (mergeCutHeight = 0.15). Each module was summarized by the first principal component of the scaled module expression profiles, termed module eigengene (ME). For each module, the module membership (MM) was defined as the correlation between lipid abundance values and ME. Intramodular hub lipids are those with the highest connectivity to other lipids within a given module, and were selected based on the *p* values of MM. For *APOE* genotype associations, module eigengenes were analyzed using pairwise linear regression models comparing each genotype group with *APOE3/*3 as the reference group, with sex included as a covariate. For each pairwise comparison, module eigengenes were evaluated using a binary genotype indicator representing the two genotype groups being compared. Modules significantly correlated with *APOE* genotype were visualized using VisANT^81^ and annotated using LION enrichment analysis^82^.

### Statistics and reproducibility

No statistical method was used to predetermine sample size. Statistical analyses were performed in R 4.3.0 using linear models (lm function) to evaluate the effects of *APOE* genotype while adjusting for sex. For each variable, genotype and sex were included as independent factors (Value ~ Sex + APOE), and ANOVA was used to assess overall genotype effects. Post hoc pairwise comparisons among *APOE* genotypes were extracted from the fitted models. *P* values were adjusted using the Bonferroni correction, with significance set at < 0.0167 (0.05/3) for three pairwise comparisons. All pairwise statistical comparisons were two-sided. Data are presented as mean ± SEM. For selected endpoints showing significant genotype effects, additional sex-specific analyses were performed by stratifying the data by sex and analyzing females and males separately using one-way ANOVA followed by Tukey’s multiple-comparison test. The experiments were randomized, and investigators were blinded to allocation during experiments and outcome assessment.

### Reporting summary

Further information on research design is available in the Nature Portfolio Reporting Summary linked to this article.

## Supplementary information

Supplementary Information

Description of Additional Supplementary Files

Supplementary Data 1

Supplementary Data 2

Supplementary Data 3

Supplementary Data 4

Reporting Summary

Transparent Peer Review file

## Source data

Source Data

## Data Availability

Source Data are provided with this paper. All data supporting the findings of this study are available within the paper and its Supplementary Information/Data files. The mass spectrometry proteomics data generated in this study have been deposited to the ProteomeXchange Consortium via the PRIDE^83^ partner repository with the dataset identifier PXD070672. Source data are provided with this paper.

## Code availability

The codes used for the data analysis are openly available on Zenodo (https://zenodo.org/records/22101784).
