# Supplementary material for: APOE2 attenuates APOE4-associated amyloid pathology and related toxicity in a mouse model of Alzheimer’s disease
Source: Nat Commun. 2026 Oct 8;17:11438. doi: 10.1038/s41467-026-78407-x (PMC13737102; doi:10.1038/s41467-026-78407-x)

Supplementary Information

APOE2 attenuates *APOE4*-associated amyloid pathology and related toxicity in a mouse model of Alzheimer's disease

Kai Chen[1], Yixing Chen[1], Yingxue Ren[2], Zhen Wang[3], Ya Huang[3], Meixia Pan[4], Zonghua Li[1], Monica Castanedes Casey[1], Wenhui Qiao[1], Ji Shi[5], Dehui Kong[6,7], Gisela Xhafkollari[1], Hannah Santhakumar[1], Allison M. Wetmore[1], E. Aubrey Thompson[5], Xusheng Wang[6,7], Dennis W. Dickson[1], David M. Holtzman[8], Guojun Bu[1,9], Xianlin Han[4], Junmin Peng[3], Na Zhao[1,*]

[1]Department of Neuroscience, Mayo Clinic, Jacksonville, FL, 32224, USA
[2]Department of Quantitative Health Sciences, Mayo Clinic, Jacksonville, FL, USA
[3]Departments of Structural Biology and Developmental Neurobiology, St. Jude Children's Research Hospital, Memphis, TN, 38105, USA
[4]Barshop Institute for Longevity and Aging Studies, University of Texas Health Science Center at San Antonio, San Antonio, TX 78229, USA
[5]Department of Cancer Biology, Mayo Clinic, Jacksonville, FL, 32224, USA
[6]Department of Neurology, University of Tennessee Health Science Center, Memphis, TN, 38163, USA.
[7]Department of Genetics, Genomics and Informatics, University of Tennessee Health Science Center, Memphis, TN, 38163, USA.
[8]Department of Neurology, Hope Center for Neurological Disorders, Knight Alzheimer's Disease Research Center, Washington University in St. Louis, St. Louis, MO, 63110, USA
[9]Present address: Division of Life Science, The Hong Kong University of Science and Technology, Hong Kong, China

*Corresponding Author: Na Zhao
e-mail: zhao.na@mayo.edu

This PDF file includes:
Supplementary Figures 1 to 12

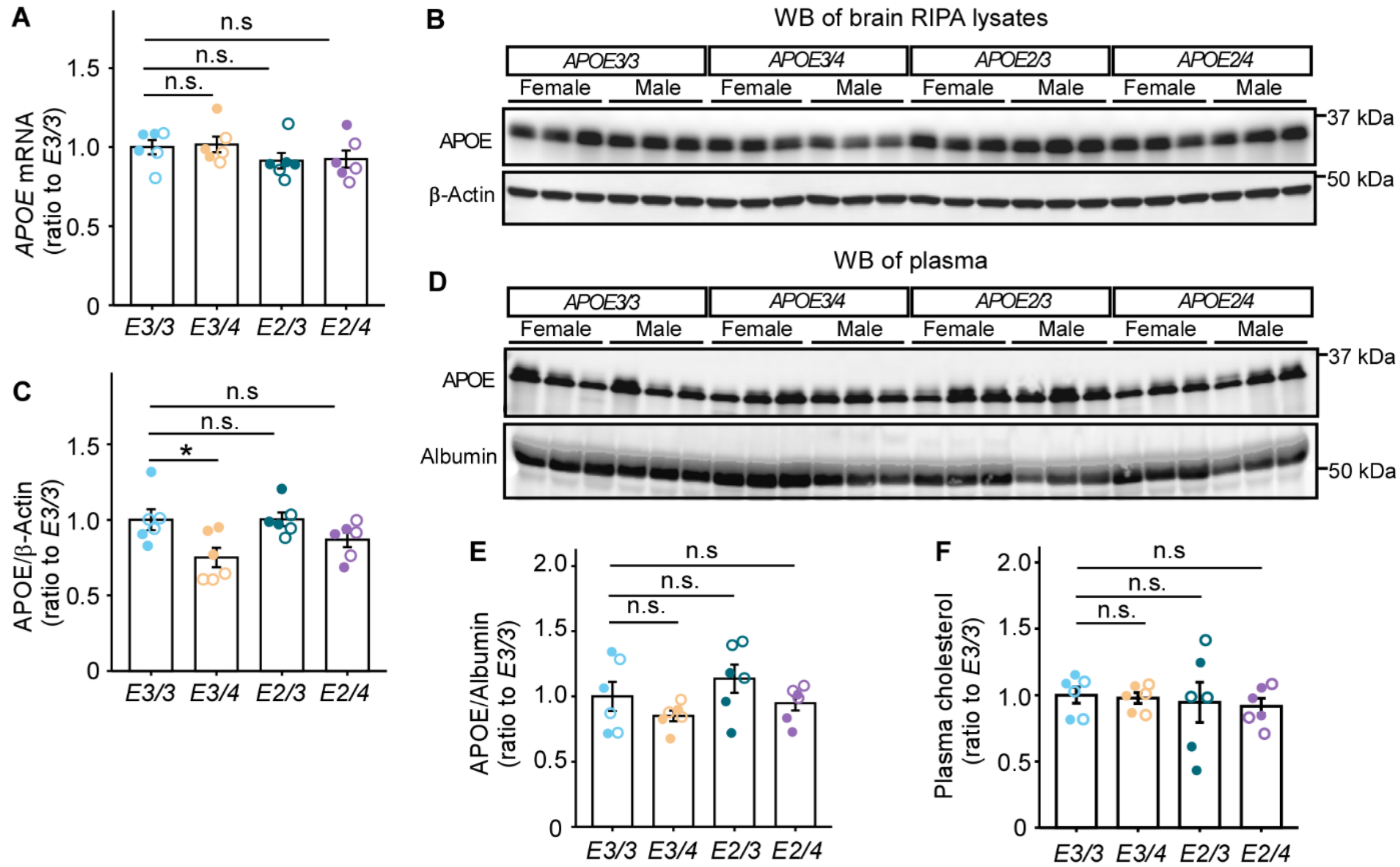

Supplementary Fig. 1: Assessment of APOE levels in the brain and plasma of heterozygous *APOE* mice.

(A) Realtime PCR showing *APOE* mRNA levels in the cortical tissue.

(B and C) Western blotting showing APOE protein levels in the RIPA lysates of cortical tissue.

(D and E) Western blotting showing APOE protein levels in plasma.

(F) Detection and quantification of cholesterol levels in plasma.

Data are presented as mean ± SEM. The data from female and male mice are labeled as solid and open circles, respectively. n = 6 mice/group. Statistical significance was determined by linear model analysis with sex included as a covariate, followed by Bonferroni multiple-comparison adjustment (significance threshold $p < 0.0167$). *$p < 0.0167$; n.s., not significant.

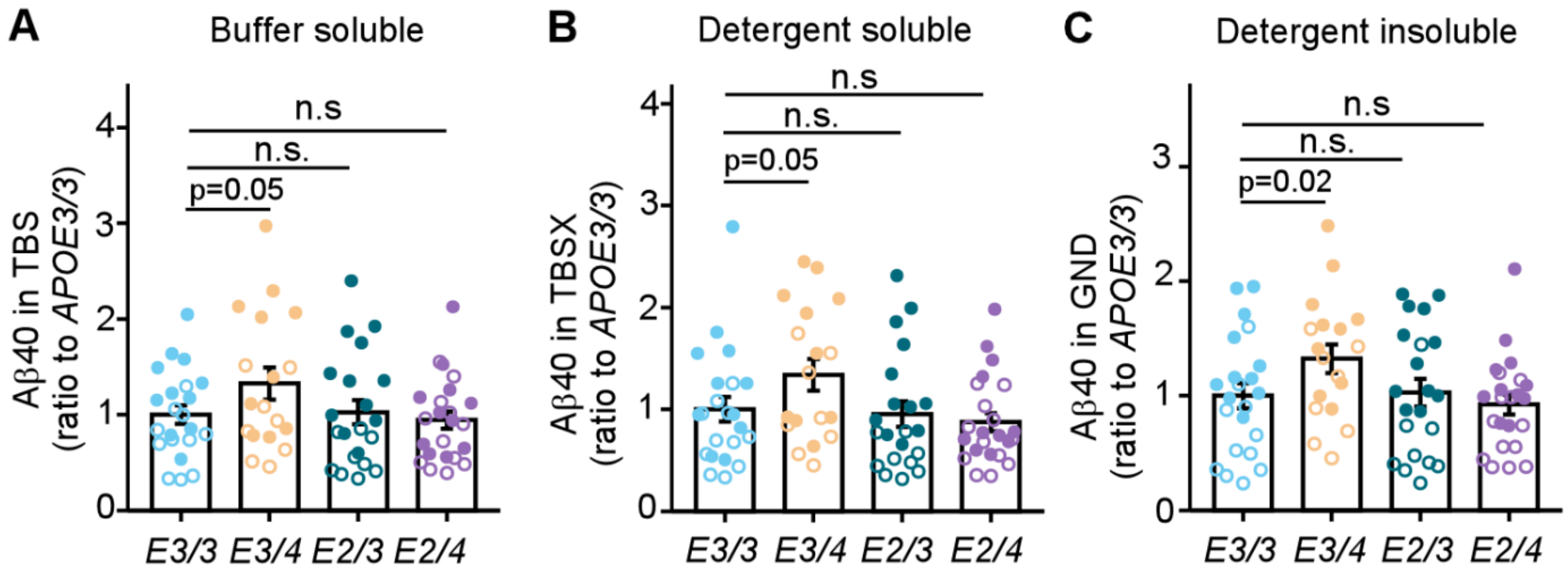

Supplementary Fig. 2: Aβ40 levels in the brain of 5xFAD-heterozygous *APOE* mice.

(A-C) Elisa detection of the Aβ40 levels in brain TBS (A), TBSX (B), and GND (C) fraction.

Data are presented as mean ± SEM. The data from female and male mice are labeled as solid and open circles, respectively. n = 18-23 mice/group. Statistical significance was determined by linear model analysis with sex included as a covariate, followed by Bonferroni multiple-comparison adjustment (significance threshold $p < 0.0167$). *$p < 0.0167$; n.s., not significant.

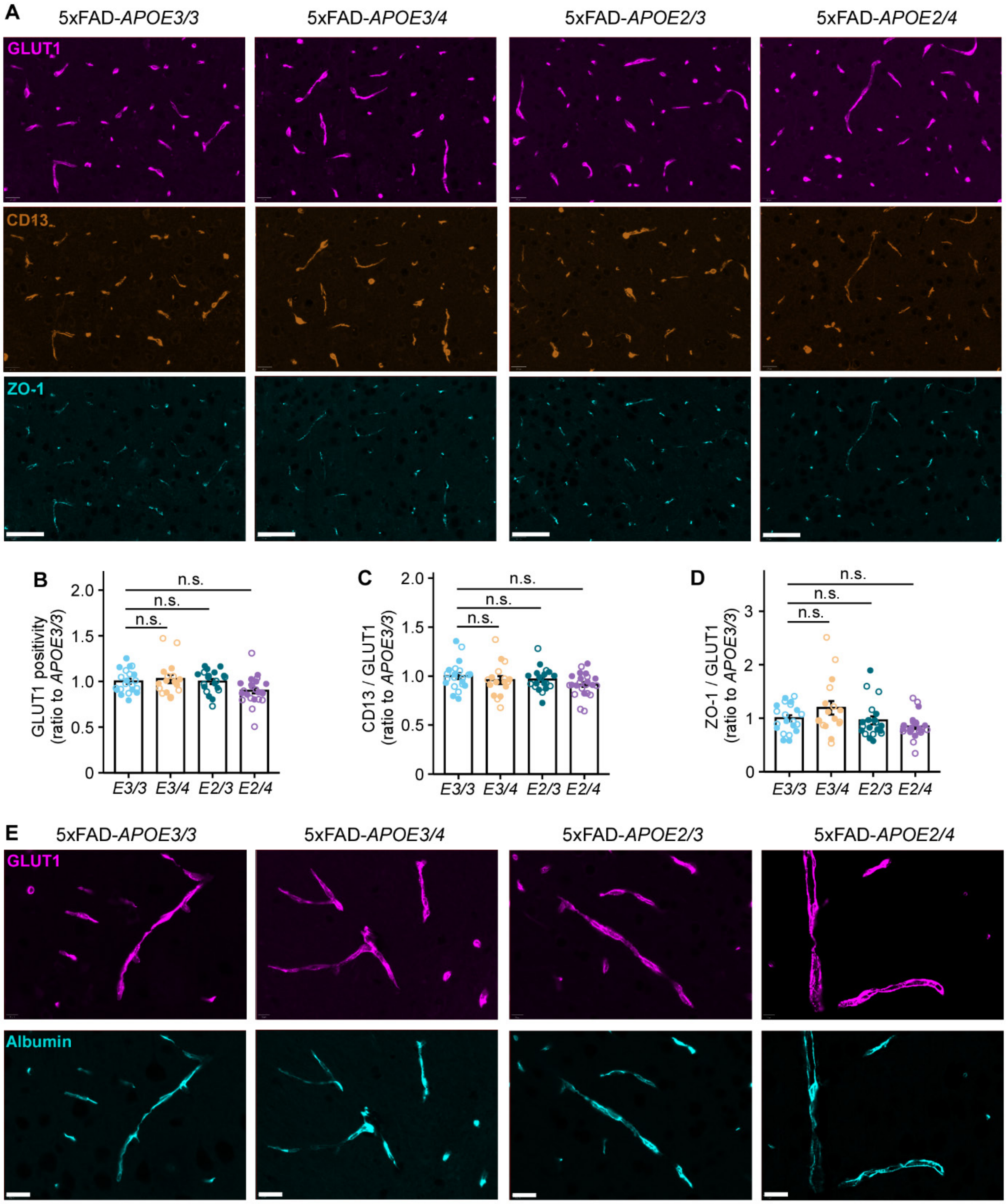

Supplementary Fig. 3: Analysis of cerebrovascular integrity in the brains of 5xFAD heterozygous *APOE* mice.

(A) Representative images of GLUT1, CD13, and ZO-1 co-immunostaining in the cortex. Scale bars, 50 μm.

(B-D) The total GLUT1 signals (B), the CD13 signals normalized against GLUT1 (C), and the ZO-1 signals normalized against GLUT1 (D) were quantified. Data are presented as mean ± SEM. The data from female and male mice are labeled as solid and open circles, respectively. n = 18-23 mice/group. Statistical significance was determined by linear model analysis with sex included as a covariate, followed by Bonferroni multiple-comparison adjustment (significance threshold $p < 0.0167$). *$p < 0.0167$; n.s., not significant.

(E) Representative images of GLUT1 and Albumin co-immunostaining in the cortex. Scale bars, 20 μm.

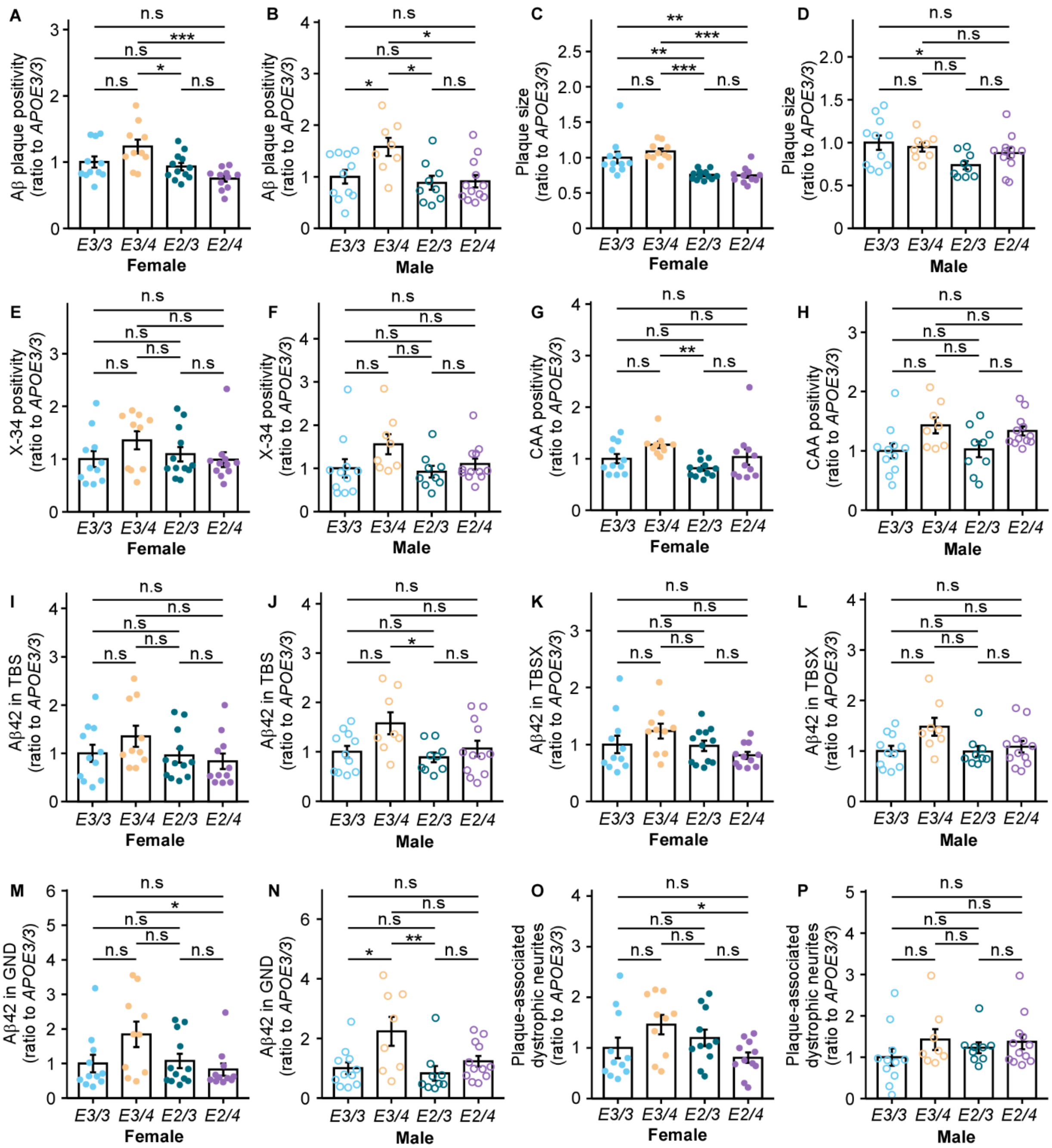

Supplementary Fig. 4: Sex-specific analysis of Aβ plaque load and neuritic dystrophy in 5xFAD-heterozygous *APOE* mice.

(A and B) Quantification of cortical Aβ plaque load in female and male mice.

(C and D) Quantification plaque size in female and male mice.

(E and F) Quantification X-34 staining of fibrillar amyloid in female and male mice.

(G and H) Quantification of leptomeningeal CAA in female and male mice.

(I and J) Quantification of Aβ42 levels by ELISA in the TBS-soluble fractions from female and male mice.

(K and L) Quantification of Aβ42 levels by ELISA in the TBSX-soluble fractions from female and male mice.

(M and N) Quantification of Aβ42 levels by ELISA in the GND-soluble fractions from female and male mice.

(O and P) Quantification of peri-plaque dystrophic neurites in female and male mice.

Data are presented as mean ± SEM. Statistical significance was determined by one-way ANOVA followed by Tukey's multiple-comparison test. $*p < 0.05$; $**p < 0.01$; $***p < 0.001$; n.s., not significant.

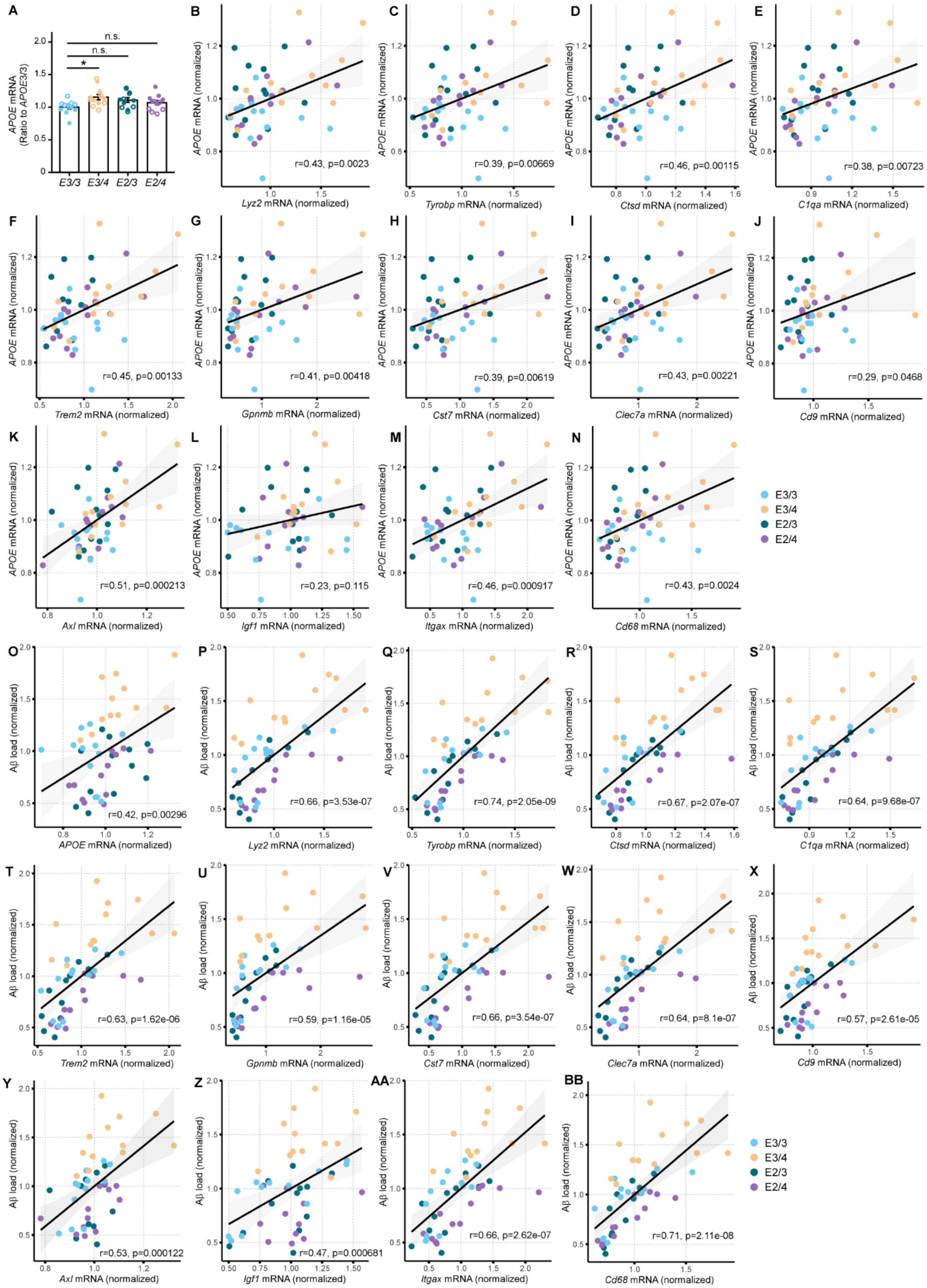
A
APOE mRNA (Ratio to APOE3/3)
n.s.
n.s.
*
E3/3
E3/4
E2/3
E2/4
B
APOE mRNA (normalized)
Lyz2 mRNA (normalized)
r=0.43, p=0.0023
C
Tyrobp mRNA (normalized)
r=0.39, p=0.00669
D
Ctsd mRNA (normalized)
r=0.46, p=0.00115
E
C1qa mRNA (normalized)
r=0.38, p=0.00723
F
Trem2 mRNA (normalized)
r=0.45, p=0.00133
G
Gpnmb mRNA (normalized)
r=0.41, p=0.00418
H
Cst7 mRNA (normalized)
r=0.39, p=0.00619
I
Clec7a mRNA (normalized)
r=0.43, p=0.00221
J
Cd9 mRNA (normalized)
r=0.29, p=0.0468
K
Axl mRNA (normalized)
r=0.51, p=0.000213
L
Igf1 mRNA (normalized)
r=0.23, p=0.115
M
Itgax mRNA (normalized)
r=0.46, p=0.000917
N
Cd68 mRNA (normalized)
r=0.43, p=0.0024
E3/3
E3/4
E2/3
E2/4
O
Aβ load (normalized)
APOE mRNA (normalized)
r=0.42, p=0.00296
P
Lyz2 mRNA (normalized)
r=0.66, p=3.53e-07
Q
Tyrobp mRNA (normalized)
r=0.74, p=2.05e-09
R
Ctsd mRNA (normalized)
r=0.67, p=2.07e-07
S
C1qa mRNA (normalized)
r=0.64, p=9.68e-07
T
Trem2 mRNA (normalized)
r=0.63, p=1.62e-06
U
Gpnmb mRNA (normalized)
r=0.59, p=1.16e-05
V
Cst7 mRNA (normalized)
r=0.66, p=3.54e-07
W
Clec7a mRNA (normalized)
r=0.64, p=8.1e-07
X
Cd9 mRNA (normalized)
r=0.57, p=2.61e-05
Y
Axl mRNA (normalized)
r=0.53, p=0.000122
Z
Igf1 mRNA (normalized)
r=0.47, p=0.000681
AA
Itgax mRNA (normalized)
r=0.66, p=2.62e-07
BB
Cd68 mRNA (normalized)
r=0.71, p=2.11e-08
E3/3
E3/4
E2/3
E2/4

Supplementary Fig. 5: Associations of brain *APOE* and disease-associated microglial gene expression with Aβ load.

(A) Brain *APOE* mRNA levels derived from the normalized *NanoString nCounter* dataset and expressed relative to *APOE3/3* controls. Data are presented as mean ± SEM. Female and male mice are indicated by solid and open circles, respectively. n = 12 mice per group. Statistical significance was determined using a linear model with sex included as a covariate, followed by Bonferroni correction for multiple comparisons (significance threshold, $p < 0.0167$). *$p < 0.0167$; n.s., not significant.

(B-N) Scatterplots showing the associations between brain *APOE* mRNA levels and the mRNA levels of disease-associated microglial (DAM) genes.

(O-BB) Scatterplots showing the associations of brain Aβ load with *APOE* mRNA levels (O) and DAM gene mRNA levels (P-BB). Gene-expression values and Aβ load were normalized to the cohort mean. Each dot represents one mouse and is colored according to *APOE* genotype. Black lines indicate linear regression fits. Pearson correlation coefficients (r) and corresponding two-sided p values are shown in each panel.

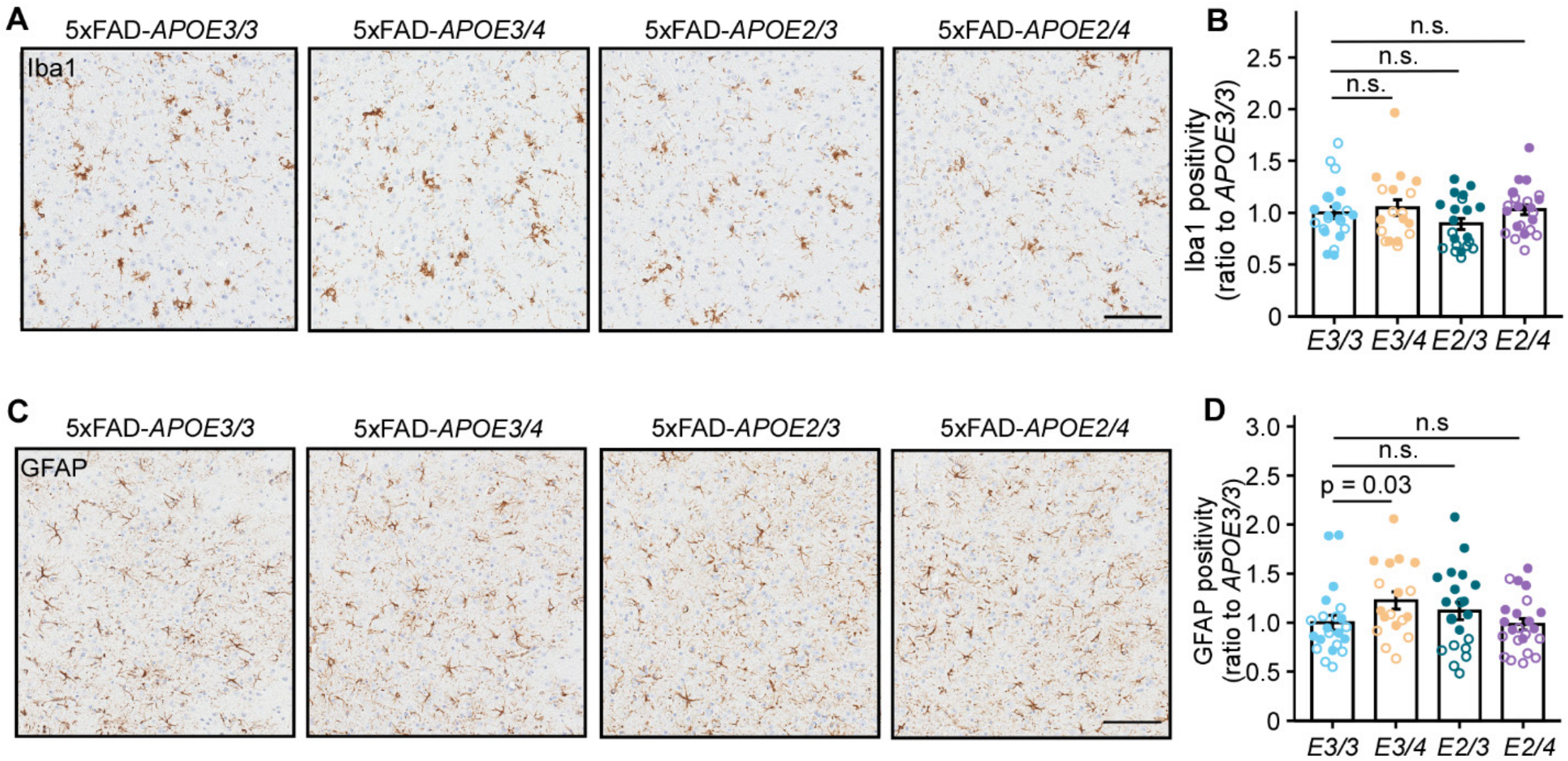

Supplementary Fig. 6: Microglia and astrocyte densities in the brain of 5xFAD-heterozygous *APOE* mice.

(A) Representative Iba1 immunostaining in the cortex of 10-month-old 5xFAD-*APOE3/3*, *APOE3/4*, *APOE2/3*, and *APOE2/4* mice. Scale bars, 100 μm.

(B) Quantification of Iba1-positive area in the cortex normalized to the *APOE3/3* group.

(C) Representative GFAP immunostaining in the cortex. Scale bars, 100 μm.

(D) Quantification of GFAP-positive area in the cortex normalized to the *APOE3/3* group.

Data are presented as mean ± SEM. The data from female and male mice are labeled as solid and open circles, respectively. n = 18-23 mice/group. Statistical significance was determined by linear model analysis with sex included as a covariate, followed by Bonferroni multiple-comparison adjustment (significance threshold $p < 0.0167$). *$p < 0.0167$; n.s., not significant.

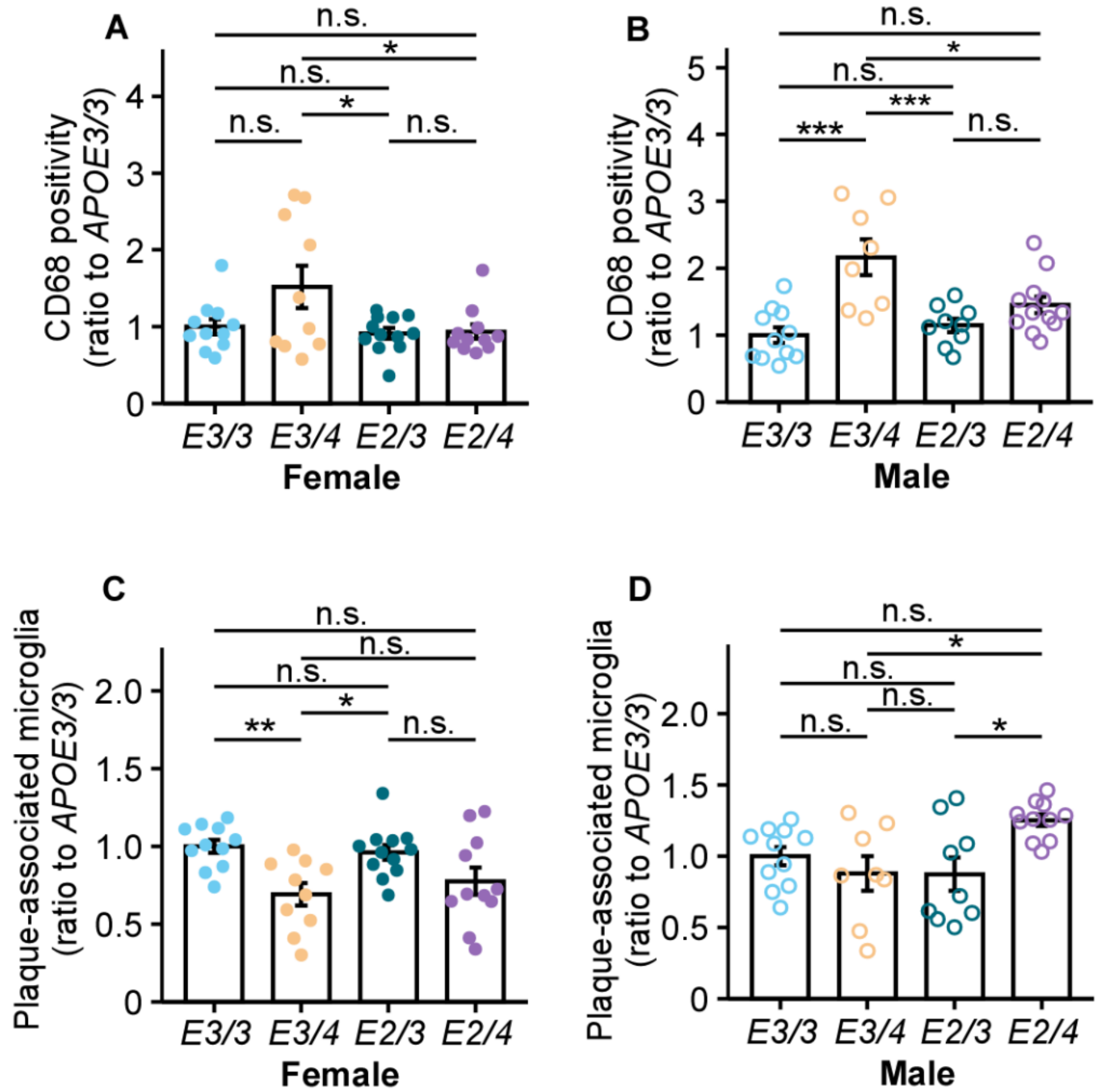

Supplementary Fig. 7: Sex-specific analysis of CD68 and plaque-associated microglia in 5xFAD-heterozygous *APOE* mice.

(A and B) Quantification of CD68 positivity in female and male mice.

(C and D) Quantification of plaque-associated microglia in female and male mice.

Data are presented as mean ± SEM. Statistical significance was determined by one-way ANOVA, followed by Tukey's multiple-comparison test. *$p < 0.05$; **$p < 0.01$; ***$p < 0.001$; n.s., not significant.

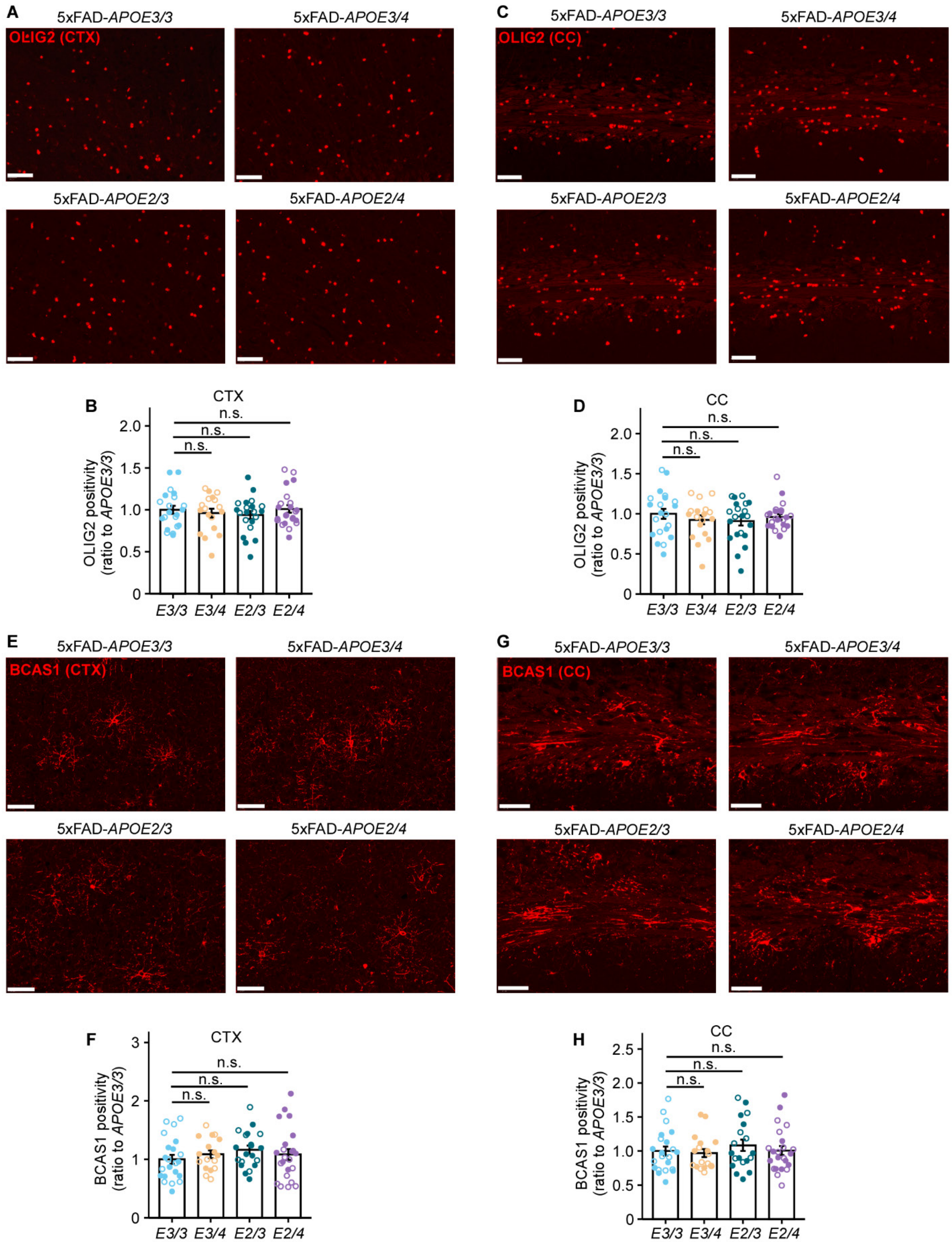

A
5xFAD-APOE3/3
5xFAD-APOE3/4
OLIG2 (CTX)
5xFAD-APOE2/3
5xFAD-APOE2/4
C
5xFAD-APOE3/3
5xFAD-APOE3/4
OLIG2 (CC)
5xFAD-APOE2/3
5xFAD-APOE2/4
B
CTX
n.s.
n.s.
n.s.
OLIG2 positivity (ratio to APOE3/3)
2.0
1.5
1.0
0.5
0
E3/3
E3/4
E2/3
E2/4
D
CC
n.s.
n.s.
n.s.
OLIG2 positivity (ratio to APOE3/3)
2.0
1.5
1.0
0.5
0
E3/3
E3/4
E2/3
E2/4
E
5xFAD-APOE3/3
5xFAD-APOE3/4
BCAS1 (CTX)
5xFAD-APOE2/3
5xFAD-APOE2/4
G
5xFAD-APOE3/3
5xFAD-APOE3/4
BCAS1 (CC)
5xFAD-APOE2/3
5xFAD-APOE2/4
F
CTX
n.s.
n.s.
n.s.
BCAS1 positivity (ratio to APOE3/3)
3
2
1
0
E3/3
E3/4
E2/3
E2/4
H
CC
n.s.
n.s.
n.s.
BCAS1 positivity (ratio to APOE3/3)
2.5
2.0
1.5
1.0
0.5
0
E3/3
E3/4
E2/3
E2/4

Supplementary Fig. 8: OLIG2 and BCAS1 densities in the brain of 5xFAD-heterozygous *APOE* mice.

(A and B) Representative OLIG2 staining in the cortex (CTX) (A) and quantification (B) normalized to *APOE3/3* controls. Scale bars, 50 μm.

(C and D) Representative OLIG2 staining in the corpus callosum (CC) (C) and quantification (D) normalized to *APOE3/3* controls. Scale bars, 50 μm.

(E and F) Representative BCAS1 staining in the CTX region (E) and quantification (F) normalized to *APOE3/3* controls. Scale bars, 50 μm.

(G and H) Representative BCAS1 staining in the CC region (G) and quantification (H) normalized to *APOE3/3* controls. Scale bars, 50 μm.

Data are presented as mean ± SEM. The data from female and male mice are labeled as solid and open circles, respectively. n = 18-23 mice/group. Statistical significance was determined by linear model analysis with sex included as a covariate, followed by Bonferroni multiple-comparison adjustment (significance threshold $p < 0.0167$). *$p < 0.0167$; n.s., not significant.

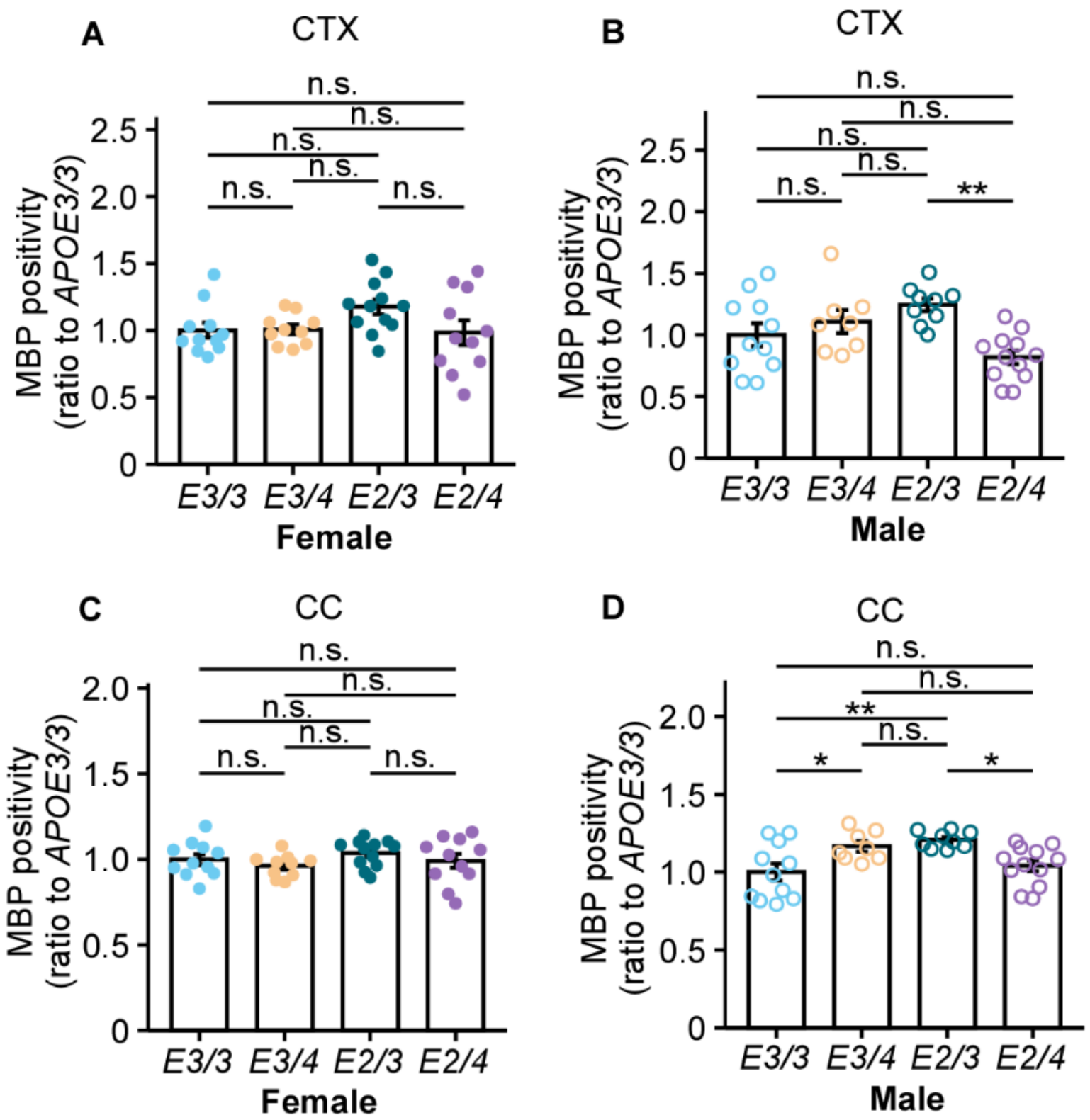

Supplementary Fig. 9: Sex-specific analysis of MBP immunoreactivity in 5xFAD-heterozygous *APOE* mice.

(A and B) Quantification of MBP positivity in the cortex (CTX) of female and male mice, respectively.

(C and D) Quantification of MBP positivity in the corpus callosum (CC) of female and male mice, respectively.

Data were normalized to *APOE3/3* controls and are presented as mean ± SEM. Statistical significance was determined by one-way ANOVA followed by Tukey's multiple-comparison test. $*p < 0.05$; $**p < 0.01$; n.s., not significant.

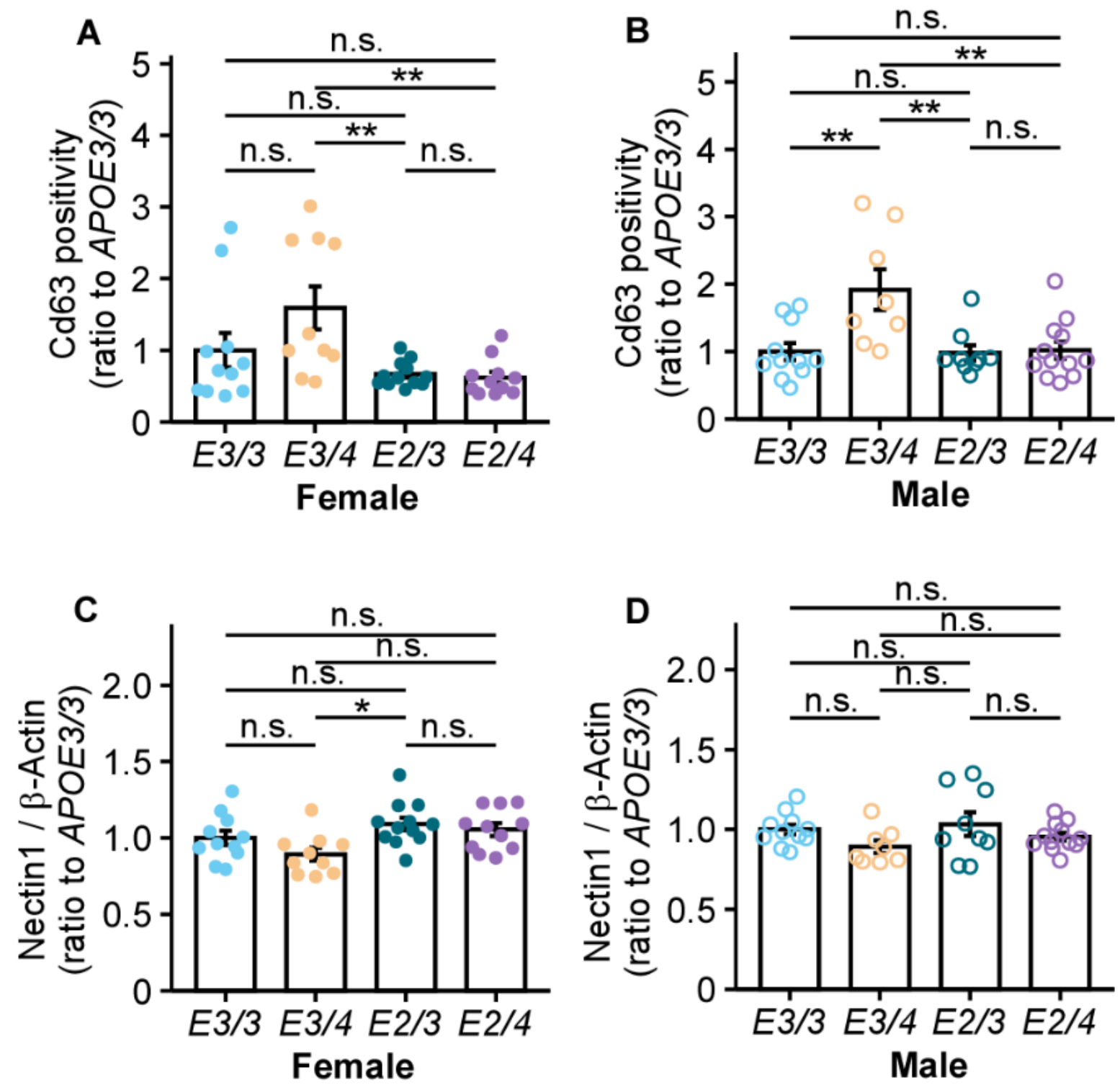

Supplementary Fig. 10: Sex-specific analysis of Cd63 immunoreactivity and Nectin1 protein level in the brain.

(A and B) Quantification of Cd63 positivity assessed by immunostaining in female and male mice, respectively.

(C and D) Quantification of Nectin-1 protein levels assessed by Western blot in female and male mice, respectively.

Data were normalized to *APOE3/3* controls and are presented as mean ± SEM. Statistical significance was determined by one-way ANOVA followed by Tukey's multiple-comparison test. $^*p < 0.05$; $^{**}p < 0.01$; n.s., not significant.

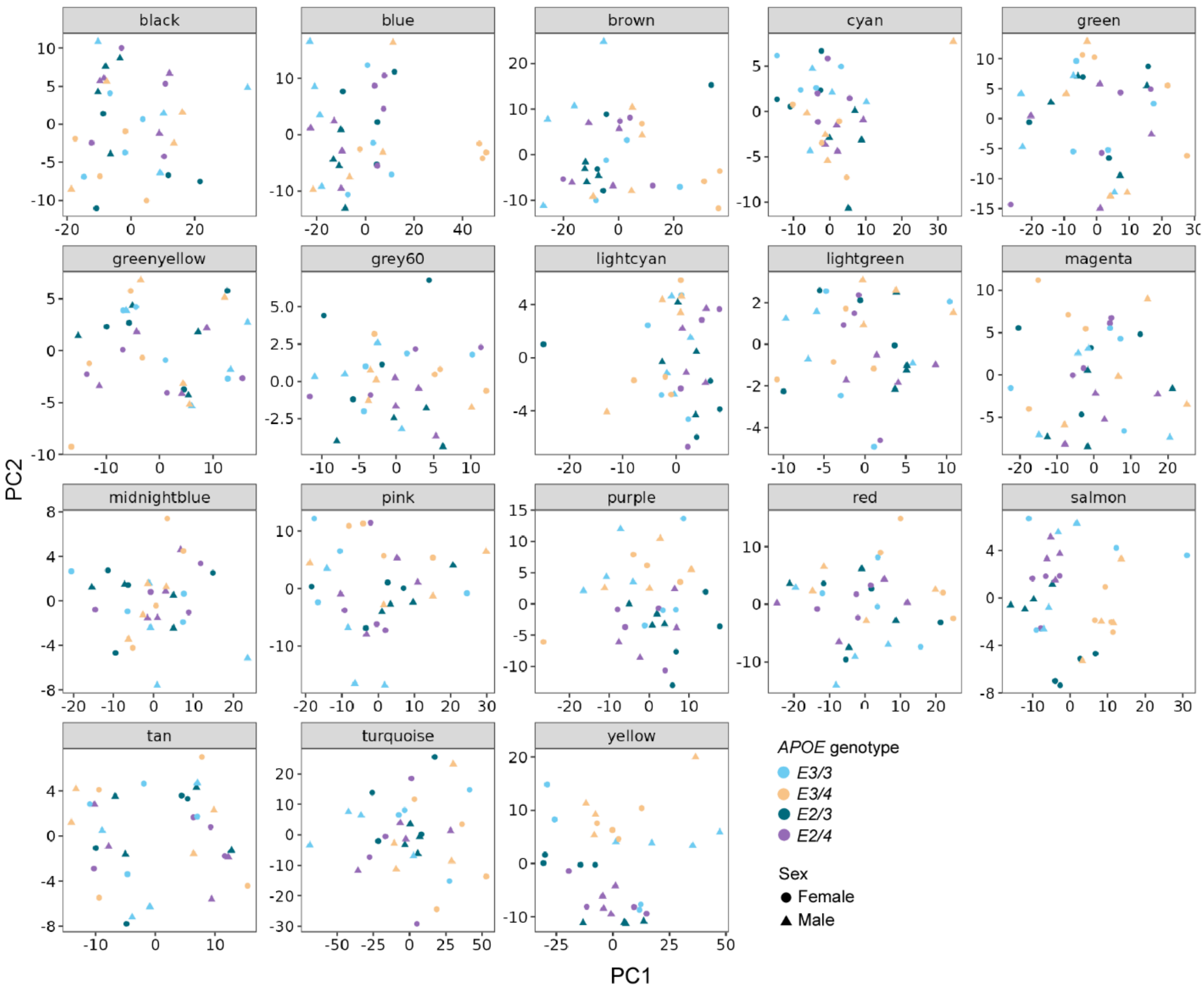

Supplementary Fig. 11: WGCNA module-specific principal component analysis of proteomic profiles in 5xFAD-heterozygous *APOE* mice.

Principal component analysis (PCA) was performed separately using the proteins assigned to each WGCNA module. Each point represents one mouse. Colors indicate *APOE* genotype, and shapes indicate sex, with circles representing female mice and triangles representing male mice. The first two principal components (PC1 and PC2) are shown for each module.

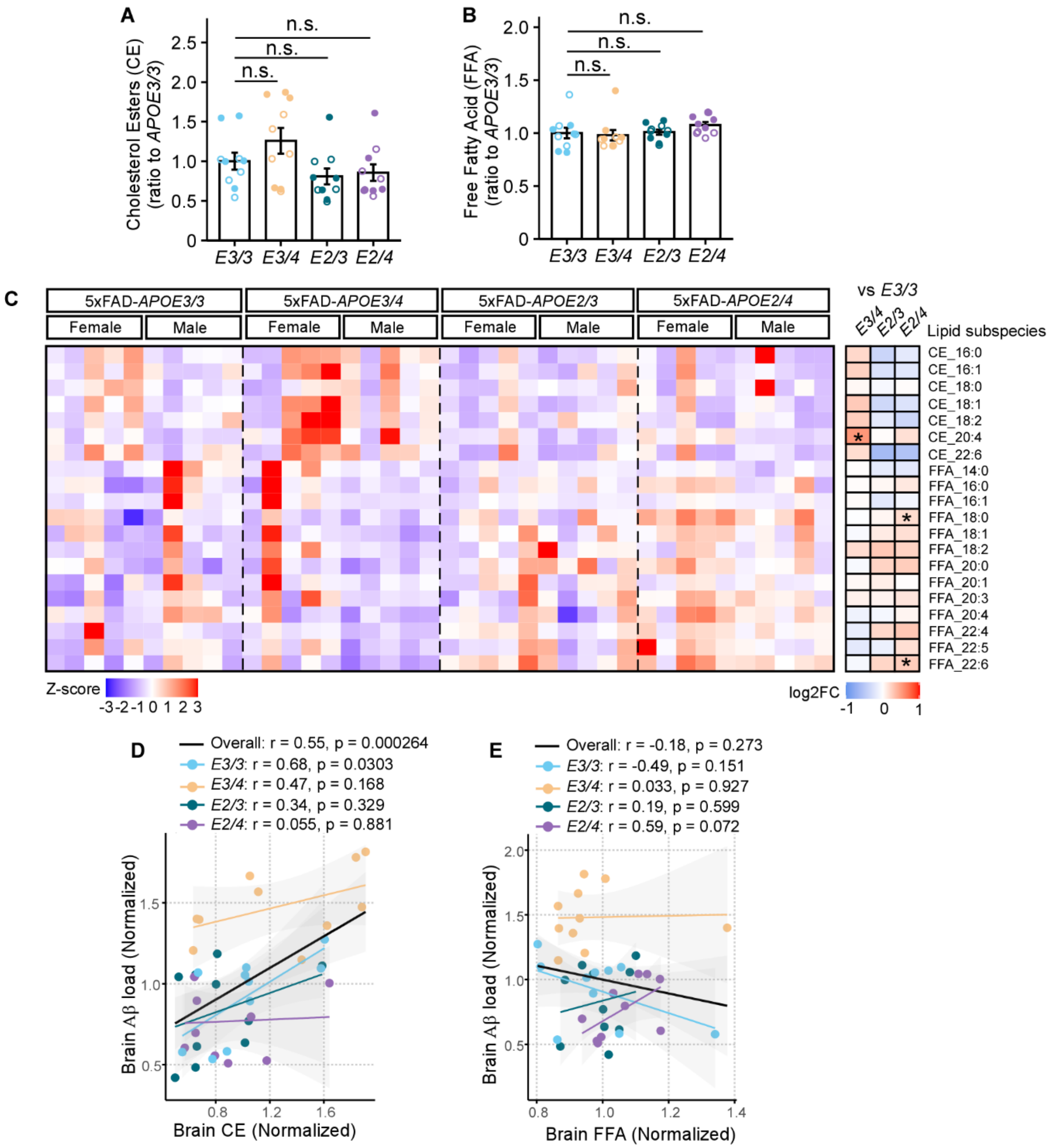

Supplementary Fig. 12: Brain CE and FFA levels and their association with Aβ burden.

(A and B) Quantification of brain total CE (A) and FFA (B) levels measured by targeted lipidomics. Data are presented as mean ± SEM. Data points from female and male mice are indicated by solid and open circles, respectively. n = 10 mice/group. Statistical significance was determined by

linear model analysis with sex included as a covariate, followed by Bonferroni multiple-comparison adjustment (significance threshold $p < 0.0167$). n.s., not significant.

(C) Heatmap showing normalized Z-scores of brain CE and FFA subspecies across genotypes. right: $\log 2FC$ of the DE lipids relative to *APOE3/3*. Asterisks indicate significant changes ($FDR < 0.05$), whereas blank cells indicate nonsignificant differences. n = 10 mice/group. Sex was adjusted in the analysis.

(D and E) Scatterplots showing the associations between brain Aβ load quantified by immunostaining and brain total CE (D) or total FFA (E) levels measured by targeted lipidomics. Total CE levels were positively associated with brain Aβ load, whereas total FFA levels showed no significant association. Pearson's correlation coefficient (r) and the corresponding p value are shown. Both variables were normalized to the cohort mean. Each dot represents one mouse.

Uncropped scans of Western blots

Supplementary Fig.1B

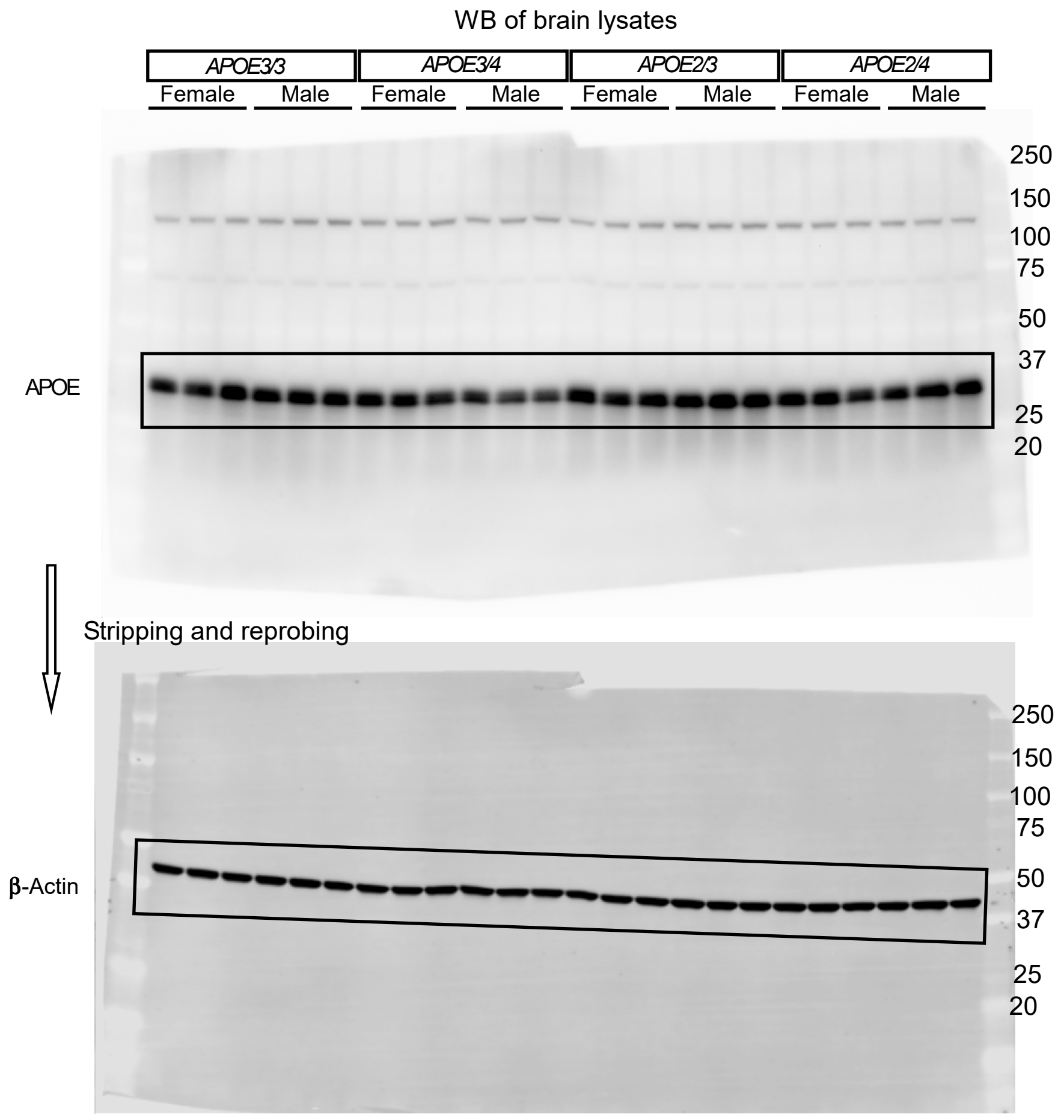

Supplementary Fig.1D

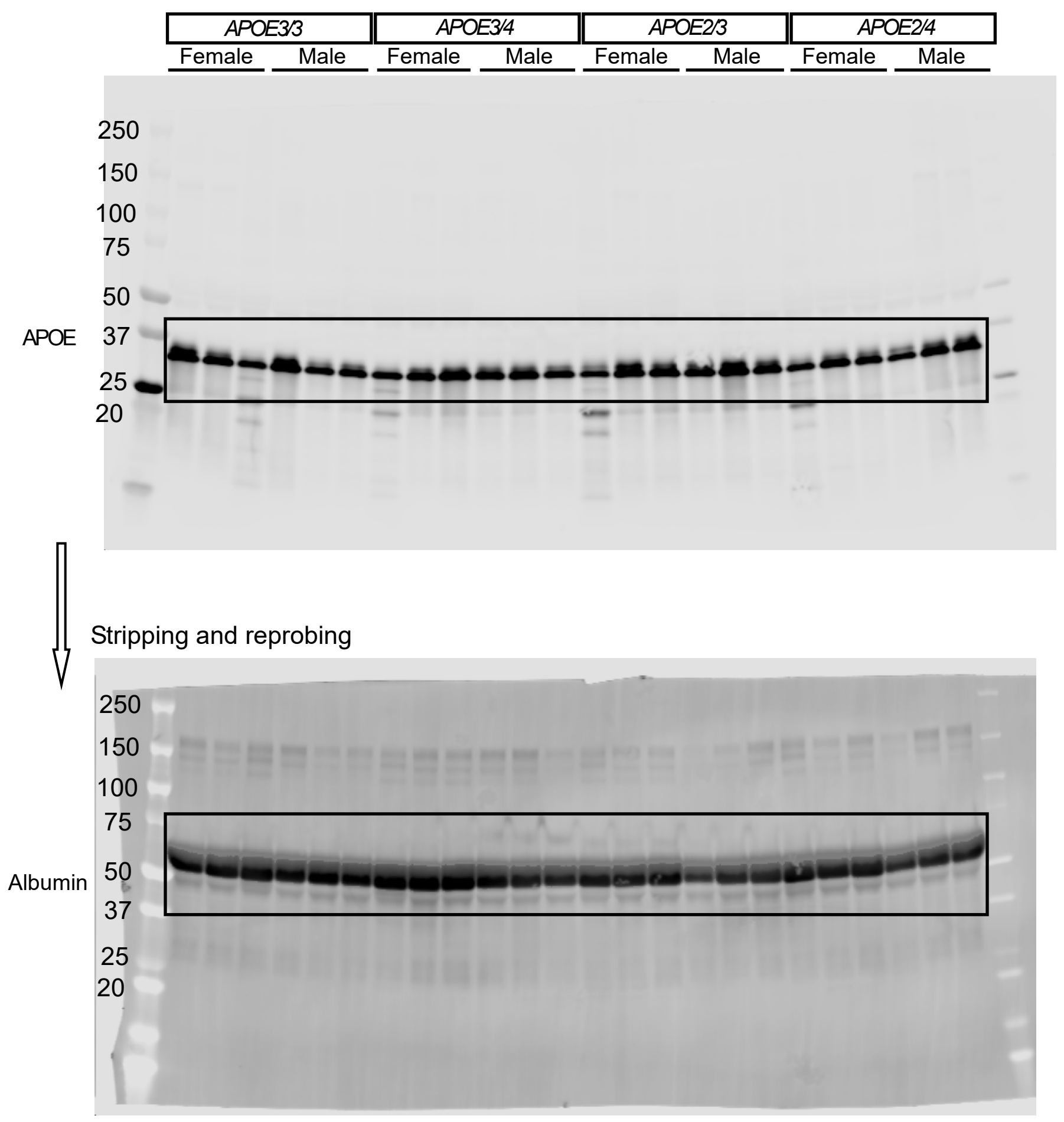

Fig. 4I

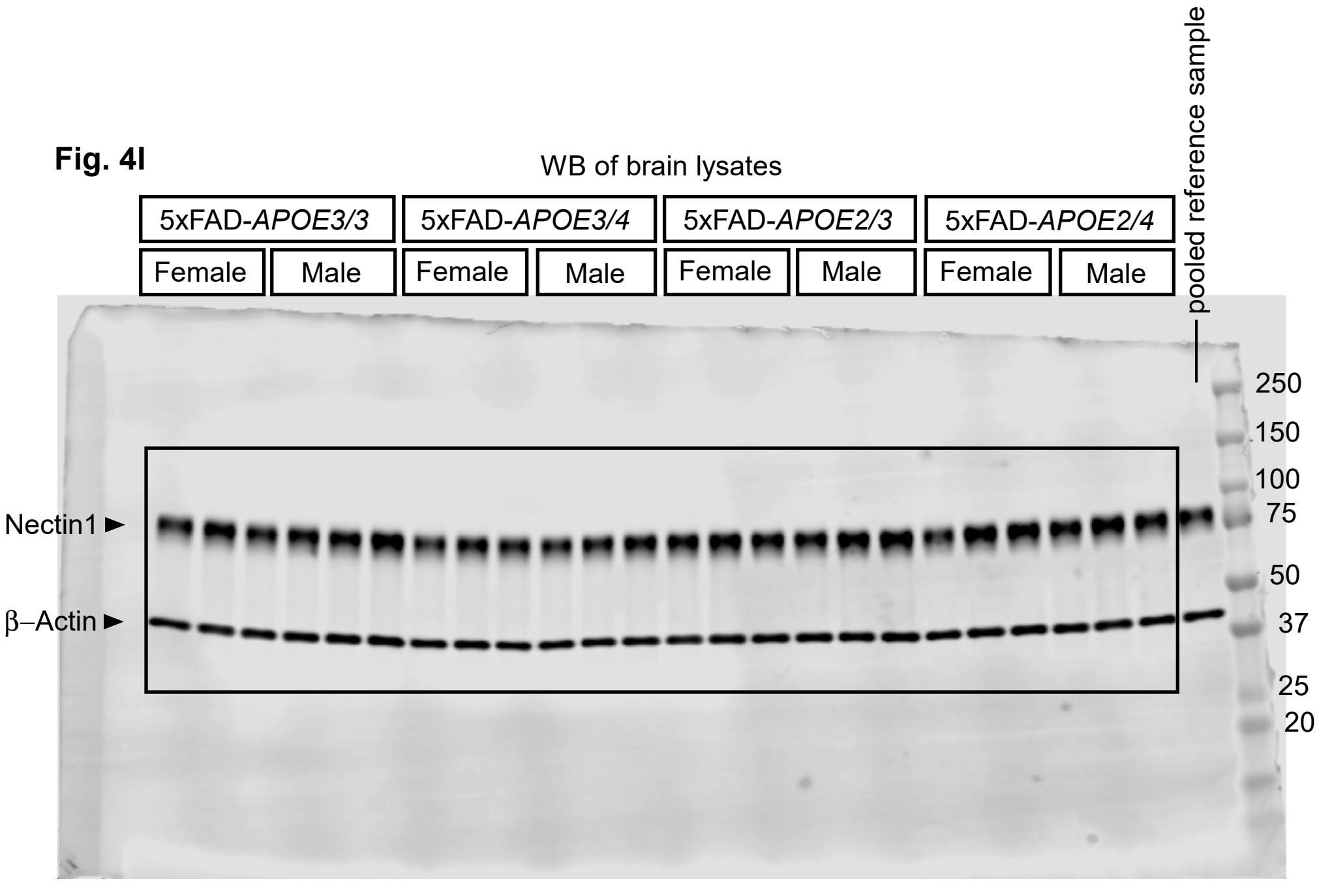

Supplement: Supplementary file 1 [file 41467_2026_78407_MOESM1_ESM.pdf]
