# Supplementary material for: APOE2 attenuates APOE4-associated amyloid pathology and related toxicity in a mouse model of Alzheimer’s disease
Source: Nat Commun. 2026 Oct 8;17:11438. doi: 10.1038/s41467-026-78407-x (PMC13737102; doi:10.1038/s41467-026-78407-x)
Supplement: Supplementary file 2 [file 41467_2026_78407_MOESM7_ESM.pdf]

nature portfolio

Corresponding author(s): Na Zhao

Last updated by author(s): Sep 17, 2026

Reporting Summary

Nature Portfolio wishes to improve the reproducibility of the work that we publish. This form provides structure for consistency and transparency in reporting. For further information on Nature Portfolio policies, see our Editorial Policies and the Editorial Policy Checklist.

Statistics

For all statistical analyses, confirm that the following items are present in the figure legend, table legend, main text, or Methods section.

n/a	Confirmed	
☐	☒	The exact sample size (n) for each experimental group/condition, given as a discrete number and unit of measurement
☐	☒	A statement on whether measurements were taken from distinct samples or whether the same sample was measured repeatedly
☐	☒	The statistical test(s) used AND whether they are one- or two-sided<br>*Only common tests should be described solely by name; describe more complex techniques in the Methods section.*
☐	☒	A description of all covariates tested
☐	☒	A description of any assumptions or corrections, such as tests of normality and adjustment for multiple comparisons
☐	☒	A full description of the statistical parameters including central tendency (e.g. means) or other basic estimates (e.g. regression coefficient) AND variation (e.g. standard deviation) or associated estimates of uncertainty (e.g. confidence intervals)
☐	☒	For null hypothesis testing, the test statistic (e.g. F, t, r) with confidence intervals, effect sizes, degrees of freedom and P value noted<br>*Give P values as exact values whenever suitable.*
☒	☐	For Bayesian analysis, information on the choice of priors and Markov chain Monte Carlo settings
☒	☐	For hierarchical and complex designs, identification of the appropriate level for tests and full reporting of outcomes
☐	☒	Estimates of effect sizes (e.g. Cohen's d, Pearson's r), indicating how they were calculated

Our web collection on statistics for biologists contains articles on many of the points above.

Software and code

Policy information about availability of computer code

Data collection	Keyence Fluorescence Microscope BZ-X810, Zeiss LSM-800 Meta confocal microscope, QuantStudio™ 7 Flex Real-Time PCR System, Leica Aperio AT2 slide scanner, BioRad ChemiDoc™ MP Imaging System, LICOR ODYSSEY CLx, Akoya PhenoImager HT 2.0, BioTek Synergy H1 Microplate Reader.
Data analysis	Aperio ImageScope (v12.4), ImageJ (v1.54d), QuPath (v0.6.0), R software (v4.3.0), nSolver Analysis Software (v4.0), QIAGEN Ingenuity Pathway Analysis (IPA), GraphPad Prism (v10.0.0), JUMP software (v1.13.4). The codes used for the data analysis have been deposited to Zenodo (https://zenodo.org/records/22101784). Detailed analysis was described in the method sessions.

For manuscripts utilizing custom algorithms or software that are central to the research but not yet described in published literature, software must be made available to editors and reviewers. We strongly encourage code deposition in a community repository (e.g. GitHub). See the Nature Portfolio guidelines for submitting code & software for further information.

Data

Policy information about availability of data

All manuscripts must include a data availability statement. This statement should provide the following information, where applicable:

- Accession codes, unique identifiers, or web links for publicly available datasets
- A description of any restrictions on data availability
- For clinical datasets or third party data, please ensure that the statement adheres to our policy

The mass spectrometry proteomics data generated in this study have been deposited to the ProteomeXchange Consortium via the PRIDE partner repository with

the dataset identifier PXD070672 (https://proteomecentral.proteomexchange.org/ui?pxid=PXD070672). All data supporting the findings of this study are available within the paper and its Supplementary Information/Data files. The codes used for the data analysis are openly available on Zenodo (https://zenodo.org/records/22101784).

Research involving human participants, their data, or biological material

Policy information about studies with human participants or human data. See also policy information about sex, gender (identity/presentation), and sexual orientation and race, ethnicity and racism.

Reporting on sex and gender	Not applicable; this study did not involve human participants, human data, or human biological materials.
Reporting on race, ethnicity, or other socially relevant groupings	Not applicable; this study did not involve human participants, human data, or human biological materials.
Population characteristics	Not applicable; this study did not involve human participants, human data, or human biological materials.
Recruitment	Not applicable; this study did not involve human participants, human data, or human biological materials.
Ethics oversight	Not applicable; this study did not involve human participants, human data, or human biological materials.

Note that full information on the approval of the study protocol must also be provided in the manuscript.

Field-specific reporting

Please select the one below that is the best fit for your research. If you are not sure, read the appropriate sections before making your selection.

[x] Life sciences [ ] Behavioural & social sciences [ ] Ecological, evolutionary & environmental sciences

For a reference copy of the document with all sections, see nature.com/documents/nr-reporting-summary-flat.pdf

Life sciences study design

All studies must disclose on these points even when the disclosure is negative.

Sample size	No statistical power analysis was used to determine sample sizes. Sample sizes was determined based on experience of previous studies and prior literature using similar experimental paradigms.
Data exclusions	For proteomic data analysis, samples Q4292 and Q4604 were excluded from downstream analyses because they were identified as outliers based on principal component analysis (PCA).
Replication	Independent animals were used as biological replicates in all experiments. Samples from each animal were measured in technical duplicates for ELISAs and biochemical analyses. Due to the long duration required to generate and age genetically defined mouse cohorts, independent replication using a separate cohort was not feasible within the scope of this study.
Randomization	Mice were randomly allocated for different experiments based on their genotypes. For other biochemical and immunohistochemical analysis, the sample order were randomized to avoid potential effects from order of samples with certain genotype.
Blinding	The investigators were blinded to the genotype during the experiments.

Reporting for specific materials, systems and methods

We require information from authors about some types of materials, experimental systems and methods used in many studies. Here, indicate whether each material, system or method listed is relevant to your study. If you are not sure if a list item applies to your research, read the appropriate section before selecting a response.

Materials & experimental systems

n/a	Involved in the study	
[ ]	[x]	Antibodies
[x]	[ ]	Eukaryotic cell lines
[x]	[ ]	Palaeontology and archaeology
[ ]	[x]	Animals and other organisms
[x]	[ ]	Clinical data
[x]	[ ]	Dual use research of concern
[x]	[ ]	Plants

Methods

n/a	Involved in the study	
[x]	[ ]	ChIP-seq
[x]	[ ]	Flow cytometry
[x]	[ ]	MRI-based neuroimaging

Antibodies

Antibodies used	Mouse monoclonal APP-NT, Millipore Sigma, Cat# MAB348; RRID:AB_94882<br>Rabbit polyclonal ASPA, Millipore Sigma, Cat# ABN1698; RRID: AB_2827931.<br>Mouse monoclonal MBP, R&D, Cat# MAB42282; RRID: AB_3658268.<br>Rabbit polyclonal IBA1, Wako, Cat# 019-19741, RRID: AB_839504.<br>Rabbit polyclonal GFAP, BioGenex, Cat# PU020-UP.<br>Goat polyclonal APOE, Meridian Life Science Cat# K74180B, RRID:AB_150544.<br>Mouse monoclonal β-actin, Sigma-Aldrich Cat# A2228, RRID:AB_476697.<br>Rabbit polyclonal Albumin, Cell Signaling Technology Cat# 4929, RRID:AB_2225785.<br>Rabbit polyclonal CD63, Proteintech, Cat# 32604-1-AP, RRID:AB_3742673.<br>Rabbit monoclonal Nectin1, Abcam, Cat# ab323704.<br>Mouse monoclonal ZO-1, Invitrogen, Cat# 33-9100, RRID:AB_2533147.<br>Goat polyclonal Albumin, Bethyl Laboratories, Cat# A90-134, RRID:AB_67120.<br>Goat polyclonal CD13, R and D Systems, Cat# AF2335, RRID:AB_2227288.<br>Rabbit polyclonal Glut1, Abcam, Cat# ab15309, RRID:AB_301844.<br>Rabbit monoclonal Olig2, Abcam, Cat# ab109186, RRID:AB_10861310.<br>Rabbit polyclonal BCAS1, Synaptic Systems, Cat# 445 003, RRID:AB_2864793.<br>Pan-Abeta (Abeta 33.1.1; In-house)
Validation	APP-NT: https://www.sigmaaldrich.com/US/en/product/mm/mab348. PMID: 31759806. This antibody was quality tested using brain paraffin sections.<br>ASPA: https://www.sigmaaldrich.com/US/en/product/mm/abn1698. PMID: 32661025. This antibody was quality tested using brain paraffin sections.<br>MBP: https://www.rndsystems.com/products/human-mouse-rat-mbp-antibody-932908_mab42282#product-datasheets. PMID: 34433069. This antibody was quality tested using the brain paraffin sections.<br>IBA1: https://www.fujifilmcdi.com/anti-iba1-polyclonal-antibody-019-19741. PMID: 30348994. This antibody was validated in previous studies and quality-tested with brain paraffin sections.<br>GFAP: https://biogenex.com/wp-content/uploads/2019/11/932-020P-EN.pdf. PMID: 30348994. This antibody was validated in previous studies and quality-tested with brain paraffin sections.<br>APOE: https://www.meridianbioscience.com/lifescience-product/k74180b. PMID: 30348994. This antibody was validated in previous studies and quality-tested with Western blot analysis.<br>β-actin: https://www.sigmaaldrich.com/US/en/product/sigma/a2228. PMID: 30348994. This antibody was validated in previous studies and quality-tested with Western blot analysis.<br>Albumin: https://www.cellsignal.com/products/primary-antibodies/albumin-antibody/4929. PMID:38086389. This antibody was validated in previous studies and quality-tested with Western blot analysis.<br>Cd63: https://www.ptglab.com/products/CD63-Antibody-32604-1-AP.htm<br>Nectin1: https://www.abcam.com/en-us/products/primary-antibodies/pvrl1-nectin1-antibody-epr29038-520-ab323704. This antibody was quality-tested with Western blot analysis.<br>ZO-1: https://www.thermofisher.com/antibody/product/ZO-1-Antibody-clone-ZO1-1A12-Monoclonal/33-9100. PMID: 35915180. This antibody was validated in previous studies and quality-tested with mouse brain sections.<br>Albumin: https://www.thermofisher.com/antibody/product/Mouse-Albumin-Antibody-Polyclonal/A90-134A. PMID: 35915180. This antibody was validated in previous studies and quality-tested with mouse brain sections.<br>CD13: https://www.rndsystems.com/products/mouse-aminopeptidase-n-cd13-antibody_af2335. PMID:38897208. This antibody was validated in previous studies and quality-tested with mouse brain sections.<br>Glut1: https://www.abcam.com/en-us/products/primary-antibodies/glucose-transporter-glut1-antibody-ab15309. PMID: 35915180. This antibody was validated in previous studies and quality-tested with mouse brain sections.<br>Olig2: https://www.abcam.com/en-us/products/primary-antibodies/olig2-antibody-epr2673-oligodendrocyte-marker-ab109186. PMID:39885546, PMID:40780195, PMID:40645178. This antibody was validated in previous studies and quality-tested with mouse brain sections.<br>BCAS1: https://sysy.com/product/445003. PMID:41483808. This antibody was validated in previous studies and quality-tested with mouse brain sections.<br>Pan-Abeta: PMID: 35915180. This antibody was validated in previous studies and quality-tested with mouse brain sections.

Animals and other research organisms

Policy information about studies involving animals; ARRIVE guidelines recommended for reporting animal research, and Sex and Gender in Research

Laboratory animals	5xFAD amyloid model mice were from The Jackson Laboratory (stock # 34848). The homozygous APOE knock-in mice (APOE2/2, APOE3/3, APOE4/4) were obtained from Dr. David M. Holtzman's lab. Heterozygous APOE mice (APOE2/3, APOE3/4, and APOE2/4) and APOE3/3 control mice were generated through a stepwise cross of homozygous human APOE knock-in mice.
Wild animals	No wild animals were used.
Reporting on sex	We used both male and female mice in all the assessments. Sex was considered as a covariate in the study.
Field-collected samples	No field-collected samples were used.

Ethics oversight	All animal procedures were approved by the Mayo Clinic Institutional Animal Care and Use Committee (IACUC) under protocol number A00003820-18-R24, and performed in accordance with the National Institutes of Health Guidelines for the Care and Use of Laboratory Animals.

Note that full information on the approval of the study protocol must also be provided in the manuscript.

Plants

Seed stocks	Not applicable.
Novel plant genotypes	Not applicable.
Authentication	Not applicable.
