# Supplementary material for: APOE2 attenuates APOE4-associated amyloid pathology and related toxicity in a mouse model of Alzheimer’s disease
Source: Nat Commun. 2026 Oct 8;17:11438. doi: 10.1038/s41467-026-78407-x (PMC13737102; doi:10.1038/s41467-026-78407-x)
Supplement: Supplementary file 3 [file 41467_2026_78407_MOESM8_ESM.pdf]

nature portfolio

Peer Review File

APOE2 attenuates APOE4-associated amyloid pathology and related toxicity in a mouse model of Alzheimer's disease

Corresponding Author: Dr Na Zhao

This file contains all reviewer reports in order by version, followed by all author rebuttals in order by version.

Version 0:

Reviewer comments:

Reviewer #1

(Remarks to the Author)
This is a well written and timely report describing the opposing effects ApoE2 and ApoE4 on plaque deposition in an animal model of Alzheimer's disease. However, the finding is not considered novel and the research results are incremental from what is already known about ApoE variants in human AD risk, clinical manifestation and biomarker associations. Further, the lack of accounting for the previously documented effects of sex on the ApoE phenotype prevents full support for this manuscript. Major revisions are required especially regarding stratification of all analyses by sex. Major differences have been described for ApoE mediated phenotypes for female and male mice. All analyses including module analyses should be stratified by sex. This report may be better suited for another journal based on lack of novelty and the standard approaches.

Major concerns:

Figure 2E the microglia associated with plaques doesn't look different between male mice (open circles). Since ApoE phenotype has been shown to be strongly dependent on sex, this analysis should be stratified by sex. It is not clear if Apo3/4 differs between 2/3 and 2/4 which should also be considered. ANOVA should be used to compare all groups stratified by sex instead of linear modeling.

Fig 3C and 3E. Sex looks to be a driver in these analyses as well. Data should be stratified by sex and reported.

Figure 4. A PCA or UMAP plot should be shown for all modules.

Figure 5K. Each genotype have should have its own linear fit so genotypes can be compared. A single linear fit across multiple genotypes is difficult to interpret.

Reviewer #2

(Remarks to the Author)
The authors present a careful characterization of APOE heterozygosity in the 5xFAD mouse model, examining amyloid-β pathology, neuritic dystrophy, glial gene expression, brain proteomics, and peripheral lipidomics. The study is well powered, includes both sexes, and the data are clearly presented.
However, the main findings are largely descriptive and, in several cases, replicate what has already been shown in homozygous APOE models. The potentially interesting idea that APOE4 may mitigate some protective effects of APOE2 (or vice versa) is not experimentally pursued. Multiple observations could form the basis of clear, testable hypotheses, but the manuscript does not follow through on these opportunities.

1. There is an apparent paradox in the plaque characterization. APOE2 plaques are described as denser and of higher intensity. Does higher intensity staining indicate a more pathological or a more protective state? Prior work (PMID: 24948358) would suggest that more fibrillar/compact plaques are associated with greater pathology. Later, the authors discuss the fibrillar nature of Aβ. Are "dense" plaques and "fibrillar" plaques equivalent in this manuscript, or are these distinct measures (e.g., Fig. 1D–F vs. 1G–H)? This needs clarification.

2. The data on APOE levels are interesting but internally inconsistent. Supplemental Fig. S1B/C suggests lower APOE4 protein stability, yet Fig. 4C shows higher APOE levels in APOE3/4 mice. The authors should clarify whether these reflect different measurements (e.g., stability vs. steady-state abundance, different tissues, or different assays) and reconcile the interpretation.

3. The sex differences are striking. In nearly every assay that includes sex as a variable, APOE4-associated effects appear more severe in females than males, including in microglial gene expression. This is a consistent and potentially important observation. The authors should comment directly on this pattern and, at minimum, propose biological explanations or hypotheses that could be tested.

4. There is substantial variability in the gene expression data in Fig. 2. In Fig. 2A, some female E3/E4 mice show very strong expression signatures. Can these high-expressing individuals be linked to plaque burden, neuritic pathology, or APOE expression levels? Without such analysis, the data remain descriptive.

5. The manuscript repeatedly uses active language such as “E2 counteracts” or “E2 neutralizes.” This implies a specific gain-of-function mechanism that is not demonstrated here. Especially in light of recent epidemiological data suggesting that APOE3/APOE3 may itself represent a risk genotype (PMID: 41522467), the authors should avoid assigning mechanism to observational findings.

6. The proteomics dataset is extensive but does not substantially extend beyond what is already shown in the pathology and gene expression data. As presented, it reinforces existing conclusions rather than generating new insight. The most novel aspect appears to be the suggestion that the APOE2 genotype alters translational pathways, but this point is underdeveloped and reads as secondary rather than central.

7. The plasma lipidomics section is difficult to contextualize and does not clearly connect to the brain phenotypes. Although genotype-associated differences are reported, they are not deeply analyzed or mechanistically linked to the central findings. In its current form, this section adds limited insight.

Minor points:
1. Some prior studies have suggested parent-of-origin effects on APOE expression. Given the variability observed within sex and genotype, was allelic inheritance tracked? Could this account for some of the heterogeneity?
2. The use of linear mixed models is appropriate. However, the consistent use of SEM rather than SD may obscure biological variability. The authors should justify this choice.
3. Statements suggesting that peripheral measurements reflect “what the brain does to the periphery” are not supported by the data, particularly given that APOE is expressed in both compartments. These interpretations should be tempered.
4. Red/green color schemes should be avoided to improve accessibility for readers with color vision deficiencies.

Overall, the dataset is strong and clearly presented, but the manuscript would benefit from deeper mechanistic analysis, clearer reconciliation of apparent inconsistencies, and more restrained interpretation of descriptive findings. Finally to progress beyond descriptive, the manuscript would benefit from testing some of the intriguing hypotheses that arise from their findings.

Reviewer #3

(Remarks to the Author)
In the manuscript “APOE2 counteracts amyloid pathology and related toxicity in the presence of APOE4,” Chen et al, the authors investigate how the protective APOE2 allele modulates APOE4-associated amyloid pathology and related neurobiological changes in the heterozygous APOE2/4 state. Using littermate-controlled 5xFAD mice expressing human APOE2/3, APOE3/4, APOE2/4, and APOE3/3 genotypes, the authors aimed to determine whether APOE2 can offset the detrimental effects of APOE4 on amyloid deposition, microglial responses, brain proteomic profiles, myelination-related phenotypes, and plasma lipidomic signatures. The authors report that APOE3/4 mice exhibited increased amyloid burden, maladaptive microglial activation, and reduced plaque-associated microglial engagement, whereas these APOE4-associated effects were attenuated in APOE2/4 mice. They further show that APOE2/4 brains display proteomic profiles more similar to APOE3/3 and APOE2/3 mice, while plasma lipidomics revealed overlapping alterations between APOE3/4 and APOE2/4 animals. Overall, the study addresses an important and understudied question regarding APOE allele interactions in Alzheimer’s disease and provides insights into the biological impact of the APOE2/4 genotype. However, the current data do not justify the broader claims regarding mechanistic rescue, vascular relevance, or therapeutic implications. In addition, there are major concerns regarding the strength of the data, the analytical framework, and the interpretation of the results, as outlined below.
1. In human studies, APOE2/3 and APOE2/4 have both been linked to increased CAA burden, and a major unresolved question in the field is why APOE2 careers may promote vascular amyloid deposition. While the present study did not detect significant differences in CAA positivity or Aβ40 levels in APOE2 carriers. This question and discrepancy are neither examined in depth nor mechanistically addressed in the current manuscript. Moreover, the vascular analysis relies primarily on the quantification of leptomeningeal CAA only, which is insufficient to support broader claims regarding vascular protection or cerebrovascular benefit.

The authors should address additional disease-relevant endpoints central to the stroke/CAA field, including BBB disruption and leakage, vascular smooth muscle cell injury, pericyte and endothelial alterations, microhemorrhage, cerebrovascular reactivity, perivascular macrophage and tight junction integrity markers.
2. The manuscript makes a claim regarding enhanced myelination, but the evidence supporting this conclusion appears limited. At present, the argument seems to rely on MBP-related readouts. Additional oligodendrocyte markers, such as CC1, Olig2, PLP1, or MAG, also structural assessments of myelin integrity, would be necessary to substantiate this claim.
3. With regard to the proteomic data, the authors should validate key proteins from the major modules emphasized in the manuscript. Validation by western blotting and/or immunofluorescence is necessary to support the biological interpretation of these findings.
4. Without behavioral testing and cerebral blood flow assessment, it remains difficult to determine the functional relevance of the observed pathological changes; these analyses are therefore essential to support the study's conclusions.
The lipidomics section is largely exploratory, and its biological and translational claims are overstated.
1. The plasma lipidomics data overextend correlation into mechanism. At present, the study does not distinguish whether the observed plasma lipid changes are brain-derived, liver-derived, fasting-related, or secondary to lipoprotein remodeling. Therefore, the current claims regarding "systemic lipid metabolism" and a direct "brain Aβ link" are overstated and would require additional data, including brain/liver lipidomics and lipoprotein fractionation.
2. Because FFA, acyl-carnitines, and TAGs are highly sensitive to nutritional state, the current design cannot distinguish genotype effects from differential fasting responses. The authors should validate the key findings under non-fasted and/or shorter fasting conditions and explicitly test genotype-by-fasting interactions.
3. It is unclear why the lipidomics WGCNA analyses appear to use $n = 8$ mice per group, whereas the differential lipid analyses use $n = 10$ per group. Unlike the proteomics section, the lipidomics methods do not describe sample exclusion, outlier removal, or QC-based filtering at the sample level. This discrepancy should be clarified.
4. Encoding APOE genotypes as 0/1/2/3 is not an appropriate analytical strategy for these heterozygous states. This approach imposes an unjustified ordinal structure and risks introducing an artificial linear trend into the module-trait analysis. In particular, APOE2/4 should not be treated as a simple intermediate state. Genotype should instead be modeled as a categorical factor, with module eigengenes analyzed by linear regression or ANOVA including sex as a covariate.
5. The characterization of FFAs as a "robust, genotype-independent" peripheral correlate of brain Aβ accumulation is overstated and premature. The current data are limited to a cross-sectional association within a single fasting cohort and lack both independent replication and longitudinal validation. In particular, validation in an independent cohort would be necessary.
6. The mechanistic interpretation of reduced CE species is insufficiently analyzed. Whole-plasma CE measurements alone cannot distinguish altered esterification, lipoprotein remodeling, cholesterol trafficking, or hepatic lipid handling. A validation approach would be to test in APOE genotype-defined astrocytes whether the CE reduction reflects impaired esterification, altered ApoE-dependent lipid export, or changes in CE distribution.
7. The distinction between "FA" and "FFA" is insufficiently defined in the main text. The authors should provide explicit operational definitions.

If the altered omics profiles are being used to support a mechanistic claim, substantial additional functional validation would be required, such as cell culture experiments, plasma transfer studies, or knockout-based approaches targeting key molecules.

Minor
1. The authors repeated emphasis on "nominal trends" or "nominally significant" observations in the text. For example, effects with p-values such as 0.02 or 0.018 are described in a way that supports a strong narrative around soluble Aβ or neuritic toxicity, despite not meeting the authors' own corrected significance threshold. This gives the impression that marginal results are being interpreted more aggressively than the statistical framework would justify.

Version 1:

Reviewer comments:

Reviewer #1

(Remarks to the Author)
The reviews have been sufficiently addressed regarding sex specific analyses and added as Supplementary figures. Further Figure 4 and 5 have been amended.

Reviewer #2

(Remarks to the Author)
The authors have made a strong effort to address my concerns through textual revisions, and the clarifications are helpful, particularly in the pathology sections. However, they do not provide additional experimental evidence to support many of their claims. The proteomics and lipidomics sections also still feel somewhat like add-ons and are not fully integrated into the manuscript. The connection to the earlier sections and to the broader pathobiology remains unclear.

Overall, I think the work is important in showing the effects of heterozygous genotypes. At the same time, the manuscript currently reads more as a collection of observations about the model than as a unified mechanistic study. As a result, the conceptual advance beyond prior studies of homozygous models, increased APOE2 expression, or APOE isoform-switching approaches is not yet fully clear (PMID: 41219507; PMID: 38504517; PMID: 24259049). The authors may wish to more explicitly define what is uniquely learned from the heterozygous model and how these findings extend the existing literature.

Minor point:

Please review the lipid-species nomenclature and confirm the analytical confidence for the reported TAG species, including TAG C58:1, C59:8, and C60:1. TAG 59:8 would require incorporation of at least one odd-chain fatty acid, which is relatively uncommon in humans, while TAG 60:1 would likely contain multiple long- or very-long-chain fatty acids and may therefore be present at low abundance. Please confirm that these species were detected above the limits of detection and quantification.

Reviewer #3

(Remarks to the Author)
The authors added analyses of CD13, GLUT1, ZO-1, and albumin, which improve the vascular characterization of the cohort. However, none showed significant APOE genotype-dependent differences. These negative findings should not be interpreted as evidence of preserved vascular integrity, blood–brain barrier function, or cerebrovascular protection. The text should state only that no detectable differences were observed in the selected markers. More importantly, the discrepancy between the human APOE2–CAA literature and the present mouse findings is not adequately discussed. Human studies have associated APOE2-containing genotypes with increased CAA burden and CAA-related vascular injury, whereas leptomeningeal CAA in APOE2/3 and APOE2/4 mice was not significantly different from that in APOE3/3 mice. The current Discussion interprets comparable CAA levels in APOE2/4 and APOE3/3 mice as evidence that APOE2 attenuates APOE4-associated pathology. This interpretation may apply to selected parenchymal amyloid outcomes, but it is too broad when extended to CAA and cerebrovascular pathology. The data support only the narrower conclusion that the increase in leptomeningeal CAA observed in APOE3/4 mice was not detected in APOE2/4 mice under the conditions examined. The Discussion should directly address the human–mouse discrepancy, including species differences, limitations of the 5xFAD model, the single age examined, the focus on leptomeningeal CAA, and the distinction between total CAA burden and vessel-wall injury. Conclusions regarding parenchymal Aβ pathology should also be clearly separated from those regarding CAA and cerebrovascular protection.

Open Access This Peer Review File is licensed under a Creative Commons Attribution 4.0 International License, which permits use, sharing, adaptation, distribution and reproduction in any medium or format, as long as you give appropriate credit to the original author(s) and the source, provide a link to the Creative Commons license, and indicate if changes were made.
In cases where reviewers are anonymous, credit should be given to 'Anonymous Referee' and the source.
The images or other third party material in this Peer Review File are included in the article's Creative Commons license, unless indicated otherwise in a credit line to the material. If material is not included in the article's Creative Commons

license and your intended use is not permitted by statutory regulation or exceeds the permitted use, you will need to obtain permission directly from the copyright holder.
To view a copy of this license, visit https://creativecommons.org/licenses/by/4.0/

RESPONSE TO REVIEWERS' COMMENTS

Reviewer #1 (Remarks to the Author):

This is a well written and timely report describing the opposing effects ApoE2 and ApoE4 on plaque deposition in an animal model of Alzheimer's disease. However, the finding is not considered novel and the research results are incremental from what is already known about ApoE variants in human AD risk, clinical manifestation and biomarker associations. Further, the lack of accounting for the previously documented effects of sex on the ApoE phenotype prevents full support for this manuscript. Major revisions are required especially regarding stratification of all analyses by sex. Major differences have been described for ApoE mediated phenotypes for female and male mice. All analyses including module analyses should be stratified by sex. This report may be better suited for another journal based on lack of novelty and the standard approaches.

--- We thank the reviewer for the thoughtful evaluation of our study and for recognizing its timeliness and overall quality. We agree that the opposing effects of *APOE2* and *APOE4* on AD risk are well established. However, the question we set out to address is somewhat different. Rather than asking what *APOE2* or *APOE4* do individually, we wanted to understand what happens when protective and risk alleles coexist in the same individual, particularly in the *APOE2/4* genotype.

This is a surprisingly large gap in the field. Although *APOE2/3*, *APOE2/4*, and *APOE3/4* are common human genotypes, most mechanistic studies have relied on homozygous *APOE2/2*, *APOE3/3*, and *APOE4/4* mice. Those models have been extremely informative for defining isoform-specific effects, but they do not address how *APOE* alleles interact with one another. As a result, it has remained unclear why *APOE2/4* individuals have substantially lower AD risk than *APOE3/4* carriers despite carrying one *APOE4* allele.

What we found is that *APOE2/4* mice resemble *APOE3/3* mice much more closely than *APOE3/4* mice across multiple independent readouts, including amyloid burden, microglial responses, synaptic pathology, transcriptomic signatures, and proteomic profiles. At the same time, we identified biological processes, such as myelin-related phenotypes and plasma lipid profiles, where *APOE4* appears to partially attenuate *APOE2*-associated effects. We believe these findings provide insight into *APOE* allele interactions that cannot be inferred from studies of homozygous *APOE* genotypes alone.

We also appreciate the reviewer's emphasis on sex as an important biological variable. In the original manuscript, sex was included as a covariate in all primary analyses. In response to this concern, we have now performed additional sex-stratified analyses for the major pathological and biochemical endpoints and included these results in the revised supplementary figures (**new Supplementary Figs. 4A-P, 7A-D, 9A-D, and 10A-D**). Overall, the major genotype-dependent patterns were observed in both females and males. For example, *APOE3/4* mice exhibited greater amyloid burden than the other genotype groups in both sexes, while *APOE2/3* and *APOE2/4* mice remained more similar to *APOE3/3* mice (**Supplementary Fig. 4A, B**), suggesting that the primary conclusion is not driven by a single sex. Although several comparisons, such as the X-34 measurements, no longer reached statistical significance after sex stratification (**Supplementary Fig. 4E, F**), the direction and magnitude of the genotype effects were generally similar in females and males. This loss of significance likely reflects the reduced sample size within each sex. We therefore included the sex-stratified analyses in the Supplementary Information for transparency, allowing readers to directly assess the robustness of the major findings across sexes, while retaining the original sex-adjusted analyses and corresponding conclusions in the main figures.

For the transcriptomic, proteomic, and module-based analyses, we considered whether full sex stratification would be appropriate. However, the study was not designed or powered to comprehensively evaluate genotype-by-sex interactions across these high-dimensional datasets. Dividing the cohorts into genotype-by-sex subgroups would substantially reduce statistical power and increase the risk of false-negative findings. For this reason, we retained sex as a covariate in the primary omics analyses, which we believe remains the most statistically appropriate approach. The Methods section has been revised to describe both the sex-adjusted primary analyses and the additional sex-specific analyses performed for selected endpoints showing significant genotype effects (**Page 35**, **line 833-841**).

Detailed responses and new results are provided below in response to the reviewer's specific comments.

Major concerns:

1. Figure 2E the microglia associated with plaques doesn't look different between male mice (open circles). Since ApoE phenotype has been shown to be strongly dependent on sex, this analysis should be stratified by sex. It is not clear if Apo3/4 differs between 2/3 and 2/4 which should also be considered. ANOVA should be used to compare all groups stratified by sex instead of linear modeling.

--- We thank the reviewer for this helpful suggestion. We agree that sex is an important biological variable in APOE-associated phenotypes. As requested, we reanalyzed the plaque-associated microglia data separately in females and males using one-way ANOVA followed by Tukey's multiple-comparison test across all *APOE* genotype groups. These sex-stratified analyses have been added as **Supplementary Fig. 7C, D.**

Overall, the sex-stratified analyses showed patterns broadly consistent with the combined analysis. In females, *APOE3/4* mice exhibited reduced plaque-associated microglial responses compared with *APOE3/3* and *APOE2/3* mice, consistent with the overall findings. In males, the overall trend was less pronounced and several comparisons no longer reached statistical significance. Given the reduced sample size after stratification, these subgroup analyses have limited statistical power and should be interpreted cautiously. Therefore, we have included the sex-stratified analyses for transparency while retaining the sex-adjusted combined analysis as the primary analysis (**Page 10, line 236-241**).

2. Fig 3C and 3E. Sex looks to be a driver in these analyses as well. Data should be stratified by sex and reported.

--- We thank the reviewer for this suggestion. We performed sex-stratified analyses of MBP immunoreactivity in both the cortex and corpus callosum, and these results are now included in **Supplementary Fig. 9A-D**.

Overall, the genotype-dependent patterns were generally consistent with those observed in the combined analysis. While several genotype comparisons no longer reached statistical significance after stratification, the reduced sample size within each sex substantially limited statistical power. We therefore provide these analyses for transparency but interpret them cautiously and retain the sex-adjusted combined analysis as the primary analysis (**Page 11, line 256-260**).

3. Figure 4. A PCA or UMAP plot should be shown for all modules.

--- We thank the reviewer for this helpful suggestion. We have now added PCA of the eigengene values for all proteomic WGCNA modules (**new Supplementary Fig. 11**). Overall, samples from different *APOE* genotypes and sexes showed substantial overlap across most modules. A modest trend toward separation of *APOE3/4* female samples was observed in the blue and brown modules, although no distinct clustering was evident. These analyses provide an overview of sample relationships across all co-expression modules and complement the statistical analyses of module eigengenes presented in the main text (**Page 14, line 330-335**).

4. Figure 5K. Each genotype should have its own linear fit so genotypes can be compared. A single linear fit across multiple genotypes is difficult to interpret.

--- We thank the reviewer for this helpful suggestion. We have revised this figure (now new **Figure 6K**) to include genotype-specific linear regression lines in addition to the overall linear fit across all samples. This presentation allows visualization of both the overall association between plasma free fatty acid (FFA) levels and brain Aβ burden and the relationships within each *APOE* genotype group. The revised figure also includes genotype-specific Pearson correlation coefficients and corresponding P values. A significant positive correlation was observed in *APOE2/3* mice, whereas the correlations within the other genotype groups did not reach statistical significance, likely reflecting the reduced sample size after stratification by genotype. The figure legend has been updated accordingly.

Reviewer #2 (Remarks to the Author):

The authors present a careful characterization of APOE heterozygosity in the 5xFAD mouse model, examining amyloid-β pathology, neuritic dystrophy, glial gene expression, brain proteomics, and peripheral lipidomics. The study is well powered, includes both sexes, and the data are clearly presented.

However, the main findings are largely descriptive and, in several cases, replicate what has already been shown in homozygous APOE models. The potentially interesting idea that APOE4 may mitigate some protective effects

of APOE2 (or vice versa) is not experimentally pursued. Multiple observations could form the basis of clear, testable hypotheses, but the manuscript does not follow through on these opportunities.

--- We thank the reviewer for the positive assessment of the study design, statistical power, inclusion of both sexes, and data presentation.

We agree that some of the individual observations reported here are consistent with findings from previous studies of homozygous *APOE* models. However, our goal was not to revisit the well-established effects of *APOE2* and *APOE4* in isolation, but rather to address a question that has remained largely unexplored: how *APOE* alleles interact when present together in heterozygous genotypes.

We also agree that several findings from this study raise interesting mechanistic questions. For example, the attenuation of *APOE2*-associated myelination signatures in *APOE2/4* mice and the distinct lipidomic profiles observed across genotypes suggest biological processes that warrant further investigation. We view these as important future directions arising from this work.

At the same time, we believe that establishing the in vivo consequences of *APOE* heterozygosity is an important step in its own right. Although *APOE2/3*, *APOE2/4*, and *APOE3/4* genotypes are far more common in humans than *APOE2/2* or *APOE4/4*, most mechanistic studies have relied on homozygous models. As a result, it has remained unclear whether protective and risk alleles act independently, additively, or dominantly when present in the same individual. Our study was designed to address this gap by systematically comparing heterozygous *APOE* genotypes in a littermate-controlled setting.

To better reflect this point, we have revised the Discussion to more clearly distinguish the established biology of APOE isoforms from the novel insights provided by examining allele interactions in heterozygous animals. We have also expanded the Discussion to highlight specific mechanistic hypotheses generated by our findings and potential directions for future studies.

1. There is an apparent paradox in the plaque characterization. APOE2 plaques are described as denser and of higher intensity. Does higher intensity staining indicate a more pathological or a more protective state? Prior work (PMID: 24948358) would suggest that more fibrillar/compact plaques are associated with greater pathology. Later, the authors discuss the fibrillar nature of Aβ. Are "dense" plaques and "fibrillar" plaques equivalent in this manuscript, or are these distinct measures (e.g., Fig. 1D–F vs. 1G–H)? This needs clarification.

--- We thank the reviewer for raising this important point. We apologize that our description of plaque morphology was not sufficiently clear.

In this study, "dense" plaques and "fibrillar" plaques refer to related but distinct measurements. The analyses shown in Figures 1D-F are based on Aβ immunostaining and assess plaque morphology. Specifically, plaques were classified as diffuse, intermediate, or compact according to their staining intensity and appearance. Thus, the increased staining intensity observed in *APOE2*-containing genotypes reflects a higher relative proportion of compact plaques among Aβ-positive plaques rather than an increase in overall plaque burden.

In contrast, Figures 1G-H quantify X-34 staining, which labels β-sheet-rich fibrillar Aβ and provides a measure of the overall fibrillar amyloid burden within the tissue. Using this approach, we found that fibrillar Aβ burden remained highest in *APOE3/4* mice. Therefore, the plaque morphology analysis in Figures 1D-F reflects the relative distribution of plaque types, whereas the X-34 analysis in Figures 1G-H measures the absolute amount of fibrillar Aβ deposition.

We agree that increased plaque intensity or fibrillar content should not be interpreted as inherently protective. Our intention was not to suggest that compact plaques represent a less pathological state. Rather, our findings indicate that *APOE* genotype influences both the amount of deposited Aβ and the structural characteristics of the resulting plaques. Importantly, despite a greater proportion of compact plaques, *APOE2*-containing genotypes exhibited substantially lower overall amyloid burden than *APOE3/4* mice.

To address this concern, we have revised the Results (**Page 5, line 122-128**) more clearly distinguish plaque morphology from overall fibrillar amyloid burden and to avoid implying that increased plaque density or fibrillarity is inherently protective. We now discuss (**Page 20, line 485-496**) these findings more cautiously in the context of prior studies, including the work cited by the reviewer, as well as studies suggesting that diffuse plaques may be associated with greater neuritic dystrophy and toxicity than more compact plaque forms (PMID: 27196974).

We emphasize that the relationship between plaque morphology and pathogenicity remains complex and that our data primarily demonstrate that *APOE* genotype influences both the quantity and structural characteristics of amyloid deposition.

2. The data on APOE levels are interesting but internally inconsistent. Supplemental Fig. S1B/C suggests lower APOE4 protein stability, yet Fig. 4C shows higher APOE levels in APOE3/4 mice. The authors should clarify whether these reflect different measurements (e.g., stability vs. steady-state abundance, different tissues, or different assays) and reconcile the interpretation.

--- We thank the reviewer for raising this important point. We agree that the apparent discrepancy between Fig. S1B/C and Fig. 4C requires clarification.

Fig. S1B/C examines APOE protein abundance in non-5xFAD *APOE* knock-in mice under baseline physiological conditions and shows reduced cortical APOE protein levels in *APOE3/4* mice compared with *APOE3/3* controls. This finding is consistent with previous studies demonstrating lower stability and reduced steady-state levels of the APOE4 isoform.

In contrast, Fig. 4C measures APOE protein abundance in cortical tissue from 5xFAD mice in the presence of amyloid pathology using mass spectrometry. In this disease context, *APOE3/4* mice exhibit substantially greater amyloid deposition, gliosis, and microglial activation, all of which are known to induce *APOE* expression. Consistent with this interpretation, we examined *APOE* transcript levels in the 5xFAD cohort (**new Supplementary Fig. 5A**) and found significantly elevated *APOE* mRNA levels in *APOE3/4* mice relative to the other genotypes.

We therefore interpret these findings as reflecting two distinct biological phenomena: under baseline conditions, APOE4 is associated with lower protein abundance, likely due in part to reduced protein stability, whereas in the setting of amyloid pathology, disease-associated upregulation of APOE expression can overcome this intrinsic deficit and result in higher steady-state APOE levels. We have added the *APOE* mRNA data and clarified this interpretation in the revised Results section (**Page 8, line 194-200 and Page 12, line 283-286**).

3. The sex differences are striking. In nearly every assay that includes sex as a variable, APOE4-associated effects appear more severe in females than males, including in microglial gene expression. This is a consistent and potentially important observation. The authors should comment directly on this pattern and, at minimum, propose biological explanations or hypotheses that could be tested.

--- We thank the reviewer for highlighting this important point. We agree that sex differences are evident across several assays in our cohort. In general, females exhibited more severe amyloid-related phenotypes than males across *APOE* genotypes, with the difference being most apparent in *APOE3/4* mice, which also showed the greatest overall amyloid burden. Similar sex-dependent effects were observed in microglial gene expression and other pathology-associated measures.

This pattern is consistent with the well-established female-biased amyloid pathology reported in the 5xFAD model (PMID: 34654824, PMID: 41408378). As noted by the reviewer, these findings may have important biological implications. One contributing factor may be the transgenic design of the 5xFAD model, as estrogen-responsive elements within the Thy1 promoter have been reported to enhance *APP* transgene expression in females (PMID: 41408378), thereby accelerating amyloid accumulation and its downstream consequences.

However, it is also possible that *APOE*-dependent mechanisms contribute to these sex differences. Previous studies have suggested that *APOE4*-associated risk is greater in females than males and that sex hormones, microglial responses, lipid metabolism, and neuroinflammatory pathways may interact with *APOE* genotype to influence disease progression. Our findings are consistent with this possibility, particularly given the more pronounced *APOE3/4*-associated pathology and microglial alterations observed in females.

While the current study was not designed to mechanistically dissect genotype-by-sex interactions, these findings raise several testable hypotheses. For example, female-specific hormonal or immune signaling pathways may amplify *APOE4*-driven microglial responses and amyloid pathology, or *APOE4*-dependent transcriptional responses in microglia may differ between females and males. We have expanded the Discussion (**Page 23, line 540-549**) to highlight these possibilities and to emphasize the need for future studies specifically designed and powered to investigate the interaction between sex and *APOE* genotype.

4. There is substantial variability in the gene expression data in Fig. 2. In Fig. 2A, some female E3/E4 mice show very strong expression signatures. Can these high-expressing individuals be linked to plaque burden, neuritic pathology, or APOE expression levels? Without such analysis, the data remain descriptive.

--- We thank the reviewer for this insightful suggestion. To address this point, we performed correlation analyses to determine whether animals with stronger DAM-associated gene expression signatures also exhibited greater amyloid pathology and altered *APOE* expression levels.

As suggested by the reviewer, we found that higher DAM gene expression was positively associated with amyloid burden and *APOE* expression. These findings indicate that the animals with the strongest DAM signatures are also those with the most pronounced pathological changes, supporting the interpretation that the observed variability in microglial gene expression reflects differences in disease severity rather than unrelated biological variation.

Because *APOE* is highly expressed by activated glial cells in the amyloid-bearing brain, the positive association between *APOE* and DAM-associated genes is consistent with a coordinated glial response to amyloid pathology. While these analyses do not establish causality, they provide additional evidence linking the transcriptional changes observed in Figure 2A to pathological features of disease progression.

These correlation analyses have been added as a **new Supplementary Fig. 5B-5BB** and are described in the revised Results section (**Page 8, line 194-200**).

5. The manuscript repeatedly uses active language such as “E2 counteracts” or “E2 neutralizes.” This implies a specific gain-of-function mechanism that is not demonstrated here. Especially in light of recent epidemiological data suggesting that APOE3/APOE3 may itself represent a risk genotype (PMID: 41522467), the authors should avoid assigning mechanism to observational findings.

--- We thank the reviewer for this helpful comment. We agree that terms such as “counteracts,” “neutralizes,” and similar active language can imply a specific mechanism that is not directly demonstrated by the current study.

In response, we carefully reviewed the manuscript and revised the language throughout the Abstract, Results, Discussion, title, and figure legends to more clearly reflect the observational nature of the findings. Rather than implying a defined mechanism, we now describe the data in terms of genotype-associated differences. For example, statements such as “APOE2 counteracts APOE4-driven pathology” have been replaced with more descriptive language indicating that the presence of APOE2 was associated with reduced APOE4-linked amyloid pathology and related molecular changes in this model.

To further address this concern, we have revised the title from “*APOE2 counteracts amyloid pathology and related toxicity in the presence of APOE4*” to “***APOE2 attenuates APOE4-associated amyloid pathology and related toxicity***.” We believe the revised title more accurately reflects the findings of the study without implying a specific mechanistic interaction. We also added a statement to the Discussion noting that the study cannot distinguish whether the reduced pathology in *APOE2/4* mice reflects protective functions conferred by APOE2, reduced APOE4-associated activity, or both. We further outline future studies using APOE-null alleles and cell type-specific APOE manipulation to resolve these possibilities (**Page 23, lines 536–540**).

We also appreciate the reviewer’s point regarding recent epidemiological evidence suggesting that *APOE3/3* may not necessarily represent a neutral reference state in all contexts. Accordingly, we revised the manuscript throughout to clarify that our conclusions are based on comparisons among *APOE* genotypes within the 5xFAD model and are interpreted relative to *APOE3/3* littermate controls, rather than treating *APOE3/3* as a presumed neutral baseline.

Overall, we have made a concerted effort throughout the revised manuscript to distinguish association from mechanism and to avoid attributing specific biological actions to *APOE2* that were not directly tested in the current study.

6. The proteomics dataset is extensive but does not substantially extend beyond what is already shown in the pathology and gene expression data. As presented, it reinforces existing conclusions rather than generating new insight. The most novel aspect appears to be the suggestion that the APOE2 genotype alters translational pathways, but this point is underdeveloped and reads as secondary rather than central.

--- We thank the reviewer for this thoughtful comment. We agree that many of the proteomic changes are consistent with the pathological and transcriptomic findings and therefore reinforce, rather than fundamentally redefine, the major conclusions of the study. We view this concordance across multiple independent data modalities as a strength of the work, as it demonstrates that the genotype-dependent effects observed at the pathological level are also reflected at the molecular level.

We agree that the enrichment of translational and ribosomal pathways in *APOE2/3* mice is one of the more interesting observations arising from the proteomic analysis. We considered whether these findings should be emphasized more strongly; however, because the effect sizes were modest and the observations were derived from bulk cortical proteomics, we felt that elevating this result to a central mechanistic conclusion would overstate what can be supported by the current data. We therefore chose to present these findings as hypothesis-generating observations.

To better reflect the reviewer's point, we have expanded the Discussion (**Page 21, line 497-505**) to more fully address the potential significance of these pathways while also acknowledging the limitations of the current dataset. We now discuss the possibility that *APOE2*-associated differences in translational regulation may contribute to protective phenotypes and highlight several testable hypotheses arising from this observation. At the same time, we note that future studies, particularly those incorporating cell type-specific and single-cell approaches, will be needed to determine the cellular origin and functional significance of these changes.

7. The plasma lipidomics section is difficult to contextualize and does not clearly connect to the brain phenotypes. Although genotype-associated differences are reported, they are not deeply analyzed or mechanistically linked to the central findings. In its current form, this section adds limited insight.

--- We thank the reviewer for this thoughtful comment. We agree that the plasma lipidomics analyses do not establish a direct mechanistic link between peripheral lipid metabolism and the brain phenotypes observed in this study. We have therefore revised both the Results and Discussion to avoid overinterpreting these findings and to more clearly distinguish association from mechanism.

To further address this concern, and in response to Reviewer 3's request for additional analyses linking peripheral and central lipid alterations, we performed targeted analyses of cholesterol esters (CEs) and free fatty acids (FFAs) in brain tissue. These lipid classes were selected because they represented the two major lipid categories highlighted by the plasma lipidomic analyses. Interestingly, the brain lipid profiles did not mirror the plasma findings (**new Supplementary Fig. 12A-E**). Brain FFA levels were not significantly associated with Aβ load, whereas brain CE levels were positively associated with Aβ burden. These findings suggest that the plasma lipidomic changes are not simply a reflection of lipid alterations within the brain and support a more nuanced interpretation of the relationship between peripheral and central lipid metabolism (**Page 17, line 391-403**).

At the same time, we believe the lipidomic data provide a useful complementary perspective on *APOE* allele interactions. Whereas *APOE2/4* mice closely resembled *APOE3/3* mice across multiple brain pathology, transcriptomic, and proteomic measures, their plasma lipidomic profiles showed partial convergence with *APOE3/4* mice. We found this divergence between central and peripheral readouts to be interesting because it suggests that *APOE* genotype may influence molecular phenotypes differently across biological compartments. While the biological basis of this observation remains unclear, it highlights the complexity of *APOE* allele interactions beyond the brain.

We agree that the functional significance of these lipidomic differences remains unresolved. Accordingly, we now present these findings as peripheral metabolic signatures associated with *APOE* genotype rather than as direct mechanistic drivers of amyloid pathology. We have also expanded the Discussion (**Page 22, line 519-529**) to highlight testable hypotheses arising from these observations and to emphasize the need for future studies investigating how peripheral and central *APOE*-dependent pathways are related and whether specific lipid species contribute to disease risk or resilience.

Minor points:

1. Some prior studies have suggested parent-of-origin effects on APOE expression. Given the variability observed within sex and genotype, was allelic inheritance tracked? Could this account for some of the heterogeneity?

--- We thank the reviewer for this thoughtful question. In the current study, parent-of-origin inheritance of *APOE* alleles was not tracked, and animals were generated through random breeding. Therefore, we cannot determine whether maternal versus paternal inheritance contributed to the variability observed within sex and genotype groups. However, because breeding was performed randomly across cohorts, any potential parent-of-origin effects would be expected to be distributed across animals within each genotype. We have revised the Discussion (**Page 23, line 545-549**) to acknowledge this as a limitation of the current study and an interesting area for future investigation.

2. The use of linear mixed models is appropriate. However, the consistent use of SEM rather than SD may obscure biological variability. The authors should justify this choice.

--- We thank the reviewer for this helpful comment. We agree that biological variability is important to display, particularly given the inter-individual variation observed in several assays. We used SEM to represent the precision of the estimated group mean. However, all individual animal-level data points are shown in the figures, allowing readers to directly assess the underlying biological variability and distribution of the data within each group.

3. Statements suggesting that peripheral measurements reflect “what the brain does to the periphery” are not supported by the data, particularly given that APOE is expressed in both compartments. These interpretations should be tempered.

--- We thank the reviewer for this important point and agree that our original wording was overly speculative. The current data do not allow us to infer a directional relationship between brain pathology and peripheral lipid alterations, particularly given that APOE is expressed in both central and peripheral compartments. In the revised manuscript, we have tempered the language throughout the Results and Discussion. We now describe these findings as associations among *APOE* genotype, plasma lipidomic signatures, and brain Aβ burden, rather than evidence that brain pathology drives peripheral lipid alterations.

4. Red/green color schemes should be avoided to improve accessibility for readers with color vision deficiencies.

---We thank the reviewer for this helpful suggestion. In the revised manuscript, we have updated the color schemes used in the relevant figures to avoid red/green combinations.

Overall, the dataset is strong and clearly presented, but the manuscript would benefit from deeper mechanistic analysis, clearer reconciliation of apparent inconsistencies, and more restrained interpretation of descriptive findings. Finally to progress beyond descriptive, the manuscript would benefit from testing some of the intriguing hypotheses that arise from their findings.

--- We thank the reviewer for the thoughtful evaluation of our work and for the many constructive suggestions. We agree that several aspects of the manuscript benefited from additional clarification and more cautious interpretation. In response to the reviewer's comments, we have added new correlation analyses, performed additional sex-stratified analyses, clarified apparent inconsistencies in the APOE and plaque morphology data, revised several sections of the Discussion to better distinguish observation from mechanism, and expanded the discussion of hypotheses generated by our findings. While the primary goal of this study was to define the in vivo consequences of *APOE* allele interactions across heterozygous genotypes, we agree that the observations reported here raise important mechanistic questions that warrant future investigation. We appreciate the reviewer's suggestions, which have helped strengthen both the presentation and interpretation of the work.

Reviewer #3 (Remarks to the Author):

In the manuscript “APOE2 counteracts amyloid pathology and related toxicity in the presence of APOE4,” Chen et al, the authors investigate how the protective APOE2 allele modulates APOE4-associated amyloid pathology and related neurobiological changes in the heterozygous APOE2/4 state. Using littermate-controlled 5xFAD mice expressing human APOE2/3, APOE3/4, APOE2/4, and APOE3/3 genotypes, the authors aimed to determine whether APOE2 can offset the detrimental effects of APOE4 on amyloid deposition, microglial responses, brain proteomic profiles, myelination-related phenotypes, and plasma lipidomic signatures. The authors report that APOE3/4 mice exhibited increased amyloid burden, maladaptive microglial activation, and reduced plaque-associated microglial engagement, whereas these APOE4-associated effects were attenuated in APOE2/4 mice. They further show that APOE2/4 brains display proteomic profiles more similar to APOE3/3 and APOE2/3 mice,

while plasma lipidomics revealed overlapping alterations between APOE3/4 and APOE2/4 animals. Overall, the study addresses an important and understudied question regarding APOE allele interactions in Alzheimer's disease and provides insights into the biological impact of the APOE2/4 genotype. However, the current data do not justify the broader claims regarding mechanistic rescue, vascular relevance, or therapeutic implications. In addition, there are major concerns regarding the strength of the data, the analytical framework, and the interpretation of the results, as outlined below.

--- We thank the reviewer for the thoughtful evaluation of our work and for recognizing the importance of understanding *APOE* allele interactions in the *APOE2/4* genotype. We appreciate the reviewer's positive assessment of the study design and the biological insights provided by the littermate-controlled heterozygous *APOE* models.

We agree that several aspects of the original manuscript could have been interpreted as implying stronger mechanistic or translational conclusions than are directly supported by the data. In response to the reviewer's comments, we have carefully revised the manuscript to more clearly distinguish observation from mechanism and association from causality. Specifically, we have tempered language related to mechanistic rescue, vascular implications, and therapeutic relevance, and we now more explicitly acknowledge the limitations of the current study.

At the same time, we believe the data provide important insight into how *APOE* alleles interact *in vivo*, particularly in the clinically relevant *APOE2/4* genotype. Our goal was to establish the biological consequences of *APOE* heterozygosity across multiple pathological and molecular domains rather than to define the precise mechanisms underlying these effects. We have revised the Discussion throughout to better reflect this scope and to highlight the mechanistic hypotheses generated by our findings.

We address each of the reviewer's specific concerns in detail below.

1. In human studies, APOE2/3 and APOE2/4 have both been linked to increased CAA burden, and a major unresolved question in the field is why APOE2 careers may promote vascular amyloid deposition. While the present study did not detect significant differences in CAA positivity or Aβ40 levels in APOE2 carriers. This question and discrepancy are neither examined in depth nor mechanistically addressed in the current manuscript. Moreover, the vascular analysis relies primarily on the quantification of leptomeningeal CAA only, which is insufficient to support broader claims regarding vascular protection or cerebrovascular benefit.

The authors should address additional disease-relevant endpoints central to the stroke/CAA field, including BBB disruption and leakage, vascular smooth muscle cell injury, pericyte and endothelial alterations, microhemorrhage, cerebrovascular reactivity, perivascular macrophage and tight junction integrity markers.

--- We thank the reviewer for raising this important point. We agree that *APOE2*-associated cerebrovascular pathology, particularly in the context of CAA, remains an important unresolved question in the field. We also agree that our original analysis, which focused primarily on leptomeningeal CAA and Aβ40 levels, was insufficient to support broader conclusions regarding vascular protection or cerebrovascular benefit.

In response to the reviewer's comment, we performed additional analyses of vascular-associated markers in the current cohort. Specifically, we assessed CD13, GLUT1, and ZO1 immunoreactivity to evaluate pericyte-associated coverage, vascular/endothelial structure, and tight junction integrity, respectively. We also performed albumin staining to assess potential blood-brain barrier leakage. These analyses did not reveal significant *APOE* genotype-dependent differences, and we did not observe obvious extravascular albumin deposition in the brain parenchyma, suggesting an absence of overt BBB disruption under the conditions examined (**new Supplementary Fig 3A-E**).

We agree that additional endpoints, including vascular smooth muscle cell injury, microhemorrhage, cerebrovascular reactivity, perivascular macrophage responses, and more comprehensive assessments of blood-brain barrier function, would provide a more complete characterization of *APOE*-dependent cerebrovascular phenotypes. These are important questions, particularly in the context of CAA and vascular contributions to neurodegeneration. However, the current study was designed to investigate how *APOE* alleles interact to influence amyloid pathology and related molecular phenotypes in heterozygous *APOE* mice rather than to comprehensively model CAA-associated vascular dysfunction. Many of the endpoints suggested by the reviewer would require dedicated experimental paradigms, including *in vivo* physiological measurements, longitudinal vascular assessments, or specialized analyses that were not incorporated into the original study

design. For this reason, we focused on adding several complementary vascular measures that could be evaluated in the existing cohort, including assessments of pericyte-associated coverage, endothelial structure, tight junction integrity, and blood-brain barrier leakage.

Accordingly, we have revised the manuscript to avoid drawing broad conclusions regarding vascular protection or cerebrovascular benefit. We now present the vascular findings more cautiously and emphasize that the relationship between *APOE* genotype, CAA, and cerebrovascular dysfunction remains an important area for future investigation (**Page 23, line 550-554**).

2. The manuscript makes a claim regarding enhanced myelination, but the evidence supporting this conclusion appears limited. At present, the argument seems to rely on MBP-related readouts. Additional oligodendrocyte markers, such as CC1, Olig2, PLP1, or MAG, also structural assessments of myelin integrity, would be necessary to substantiate this claim.

--- We thank the reviewer for this important comment and agree that MBP immunoreactivity alone is insufficient to establish enhanced myelination.

In response, we examined additional oligodendrocyte-lineage markers, including ASPA, OLIG2, and BCAS1 (**Fig.3 A-E and new Supplementary Fig. 8A-H**), to assess mature oligodendrocytes and oligodendrocyte differentiation states. We did not observe significant *APOE* genotype-dependent differences in these markers. These findings suggest that the increased MBP immunoreactivity observed in *APOE2/3* mice is not accompanied by detectable changes in oligodendrocyte abundance or differentiation. These additional data have been added to the revised Results accordingly (**Page 10, line 245-255**).

We agree that additional structural and functional assessments of myelin integrity would provide a more definitive evaluation of whether *APOE* genotype influences myelination. However, such analyses would require dedicated tissue preparation and collection procedures that were not incorporated into the original study design. For example, ultrastructural assessment of myelin architecture by electron microscopy requires tissue that is specifically processed and preserved for EM analysis, which is not available in the current cohort. As a result, these experiments cannot be rigorously performed retrospectively using the existing samples.

Accordingly, the current dataset does not allow us to determine whether the observed differences in MBP immunoreactivity reflect changes in myelin structure, myelin maintenance, or other aspects of myelin biology. We have therefore tempered our interpretation throughout the manuscript and no longer refer to these findings as evidence of enhanced myelination.

3. With regard to the proteomic data, the authors should validate key proteins from the major modules emphasized in the manuscript. Validation by western blotting and/or immunofluorescence is necessary to support the biological interpretation of these findings.

--- We thank the reviewer for this important suggestion. To provide independent support for the biological interpretation of the proteomic analyses, we performed additional validation of representative proteins from the major modules highlighted in the manuscript.

For the immune/lysosomal module, we examined CD63 by immunofluorescence staining. Consistent with the proteomic findings, CD63 immunoreactivity was enriched in microglia and was increased in *APOE3/4* mice relative to *APOE3/3* controls. For the synaptic/neuronal module, we assessed Nectin1 protein levels by Western blotting. Consistent with the proteomic data, Nectin1 levels were reduced in *APOE3/4* mice compared with *APOE3/3* mice.

These validation experiments have been added to the revised manuscript (**Fig. 4G-J, Page 13, line 311-321**) and provide independent support for the module-level changes identified by proteomics. Together, these findings further support the interpretation that *APOE3/4* mice exhibit enhanced microglial activation and disruption of synaptic-associated pathways relative to the other *APOE* genotypes.

4. Without behavioral testing and cerebral blood flow assessment, it remains difficult to determine the functional relevance of the observed pathological changes; these analyses are therefore essential to support the study's conclusions.

--- We thank the reviewer for this important comment. We agree that behavioral testing and cerebral blood flow measurements would provide valuable functional information and could further extend the findings presented here.

However, these analyses were not part of the original study design and cannot be performed retrospectively in the current cohort. More importantly, the primary objective of this study was to define how *APOE* alleles interact to influence amyloid pathology and associated molecular and cellular phenotypes in heterozygous *APOE* genotypes. Accordingly, the pathological, transcriptomic, proteomic, and lipidomic analyses presented here were intended to establish the biological consequences of *APOE* allele interactions rather than to comprehensively assess functional outcomes.

We agree that future studies incorporating behavioral assessments, cerebrovascular function, and cerebral blood flow measurements will be important for determining how the pathological and molecular differences identified here translate into functional phenotypes. To acknowledge this limitation, we have expanded the Discussion (**Page 23, line 550-554**) and tempered statements regarding functional and therapeutic implications of the current findings.

The lipidomics section is largely exploratory, and its biological and translational claims are overstated.

--- We thank the reviewer for this thoughtful assessment. We agree that several aspects of the original lipidomics section were interpreted too strongly relative to the data presented. Our intention was to determine whether *APOE* genotype and amyloid pathology were associated with distinct peripheral lipidomic signatures, rather than to establish causal mechanisms linking plasma lipid alterations to brain pathology.

In response to the reviewer's concerns, we have substantially revised the lipidomics section and associated Discussion. Specifically, we have tempered claims regarding systemic lipid metabolism, biomarker potential, brain-periphery mechanistic links, and translational relevance. We now present the plasma lipidomic findings as genotype-associated peripheral metabolic signatures observed under the experimental conditions examined, rather than as evidence of causal pathways or validated biomarkers.

In addition, we generated new data to further evaluate the relationship between plasma and brain lipid alterations, including targeted analyses of cholesterol esters (CEs) and free fatty acids (FFAs) in brain tissue. These analyses revealed distinct patterns in brain and plasma lipid compartments, supporting a more cautious interpretation of the plasma findings and arguing against a simple brain-derived explanation for the observed plasma lipidomic changes.

Throughout the revised manuscript, we have emphasized the exploratory and hypothesis-generating nature of the lipidomic analyses and have expanded the Discussion (**Page 22, line 519-529**) to outline the additional studies that will be required to establish the biological origins and functional significance of these lipid alterations.

1. The plasma lipidomics data overextend correlation into mechanism. At present, the study does not distinguish whether the observed plasma lipid changes are brain-derived, liver-derived, fasting-related, or secondary to lipoprotein remodeling. Therefore, the current claims regarding “systemic lipid metabolism” and a direct “brain Aβ link” are overstated and would require additional data, including brain/liver lipidomics and lipoprotein fractionation.

--- We thank the reviewer for this important comment and agree that the original manuscript may have overstated the mechanistic interpretation of the plasma lipidomics findings.

To address this concern, we performed additional targeted lipidomic analyses of cholesterol esters (CEs) and free fatty acids (FFAs) in brain tissue, focusing on the two major lipid classes highlighted by the plasma lipidomic dataset. These new analyses revealed a different pattern from that observed in plasma. Specifically, brain CE levels did not show significant *APOE* genotype-dependent differences, and brain FFA levels did not positively correlate with Aβ burden. In contrast, brain CE levels were positively associated with Aβ burden, suggesting a closer relationship between CE accumulation and local amyloid pathology (**new Supplementary Fig.12A-E**)

These findings indicate that lipid alterations in brain and plasma likely reflect distinct biological compartments and should not be assumed to arise from a common mechanism. Importantly, the current data do not allow us to determine whether the plasma lipid changes originate from the brain, liver, lipoprotein remodeling, or other systemic processes. Accordingly, we have revised the manuscript to avoid implying a direct mechanistic brain-

periphery lipid pathway and now present the plasma lipidomic findings as genotype-associated peripheral metabolic signatures rather than evidence of a causal link between plasma lipid changes and brain amyloid pathology.

We have also expanded the Discussion (**Page 22, line 519-529**) to acknowledge that future studies incorporating brain and liver lipidomics, lipoprotein fractionation, and metabolic flux analyses will be required to define the origins and biological significance of these plasma lipid alterations.

2. Because FFA, acyl-carnitines, and TAGs are highly sensitive to nutritional state, the current design cannot distinguish genotype effects from differential fasting responses. The authors should validate the key findings under non-fasted and/or shorter fasting conditions and explicitly test genotype-by-fasting interactions.

--- We thank the reviewer for this important point. We agree that circulating FFAs, acyl-carnitines, and TAGs can be influenced by nutritional state and that fasting may affect the abundance of these lipid species.

In the current study, all animals were subjected to the same 14-hour fasting protocol prior to sample collection. Therefore, the genotype comparisons reported here were performed under identical fasting conditions, minimizing variation arising from differences in feeding status within the cohort. However, we agree that the present design does not allow us to determine whether *APOE* genotype influences fasting responses themselves or whether the observed lipidomic differences would be maintained under non-fasted conditions.

Because non-fasted or shorter-fasted plasma samples were not collected as part of the original study design, these analyses cannot be performed retrospectively in the current cohort. We have therefore revised the manuscript to temper the interpretation of fasting-sensitive lipid species and now explicitly acknowledge that future studies incorporating multiple nutritional states will be required to assess genotype-by-fasting interactions and determine the extent to which these lipidomic signatures are influenced by feeding status.

Accordingly, we now present these findings as *APOE* genotype-associated lipidomic profiles observed under standardized fasting conditions rather than as fasting-independent alterations in systemic lipid metabolism.

3. It is unclear why the lipidomics WGCNA analyses appear to use n = 8 mice per group, whereas the differential lipid analyses use n = 10 per group. Unlike the proteomics section, the lipidomics methods do not describe sample exclusion, outlier removal, or QC-based filtering at the sample level. This discrepancy should be clarified.

--- We thank the reviewer for pointing this out. The reported sample size of n = 8 per group in the lipidomics WGCNA analysis was a typographical error. The correct sample size for both the differential lipid analyses and the WGCNA analyses was n = 10 animals per group.

No samples were excluded from the lipidomics dataset, and no sample-level outlier removal was performed prior to WGCNA. We have corrected the sample size throughout the revised manuscript.

4. Encoding APOE genotypes as 0/1/2/3 is not an appropriate analytical strategy for these heterozygous states. This approach imposes an unjustified ordinal structure and risks introducing an artificial linear trend into the module-trait analysis. In particular, APOE2/4 should not be treated as a simple intermediate state. Genotype should instead be modeled as a categorical factor, with module eigengenes analyzed by linear regression or ANOVA including sex as a covariate.

--- We appreciate the reviewer's comment and agree that treating *APOE* genotypes as a single ordinal 0/1/2/3 variable would impose an inappropriate linear structure and could lead to misleading interpretations, particularly for the *APOE2/4* genotype.

To clarify, this was not the analytical approach used in our module–trait analyses. The analyses shown in Figures 4G and 5C were performed as pairwise genotype comparisons, with sex included as a covariate. In each comparison, genotype was modeled as a two-level categorical variable corresponding to the two groups being tested. Therefore, the analyses did not assume an ordered relationship among *APOE2/3*, *APOE3/3*, *APOE2/4*, and *APOE3/4* genotypes and did not treat *APOE2/4* as an intermediate state.

We apologize that the original description was not sufficiently clear. We have revised the Methods section (**Page 33, line 783-787 and Page 35, line 825-829**) to avoid implying that the four genotypes were analyzed as a single ordered variable.

5. The characterization of FFAs as a “robust, genotype-independent” peripheral correlate of brain Aβ accumulation is overstated and premature. The current data are limited to a cross-sectional association within a single fasting cohort and lack both independent replication and longitudinal validation. In particular, validation in an independent cohort would be necessary.

--- We thank the reviewer for this important comment and agree that our original description of FFAs as a “robust, genotype-independent” peripheral correlate of brain Aβ accumulation was too strong given the scope of the current dataset.

The observed association was identified in a single cross-sectional cohort collected under standardized fasting conditions and has not been independently replicated or evaluated longitudinally. Therefore, the current data do not establish FFAs as a validated biomarker of brain amyloid burden or demonstrate that the relationship is independent of other physiological factors.

In response to the reviewer's concern, we have revised the manuscript to use more cautious language throughout. We now describe plasma FFAs as fasting-state peripheral lipid species that were associated with brain Aβ burden in the current cohort, rather than as a robust or validated biomarker. We have also expanded the Discussion (**Page 23, line 550-554**) to acknowledge that independent cohort validation and longitudinal studies will be required to determine the reproducibility, specificity, and potential biological significance of this association.

6. The mechanistic interpretation of reduced CE species is insufficiently analyzed. Whole-plasma CE measurements alone cannot distinguish altered esterification, lipoprotein remodeling, cholesterol trafficking, or hepatic lipid handling. A validation approach would be to test in APOE genotype-defined astrocytes whether the CE reduction reflects impaired esterification, altered ApoE-dependent lipid export, or changes in CE distribution.

--- We thank the reviewer for this important comment. We agree that the current plasma lipidomic data do not provide sufficient mechanistic insight to determine whether the observed reduction in CE species reflects altered cholesterol esterification, lipoprotein remodeling, cholesterol trafficking, hepatic lipid metabolism, or other processes.

To further assess the relationship between plasma and brain lipid alterations, we performed targeted lipidomic analyses of CE species in brain tissue. In contrast to the plasma findings, brain CE levels did not show significant *APOE* genotype-dependent differences (**new Supplementary Fig. 12A-E**). These results suggest that the *APOE*-associated CE changes observed in plasma are not simply mirrored in the brain and therefore do not immediately support a brain-intrinsic mechanism underlying the plasma CE phenotype.

We agree that experiments using *APOE* genotype-defined astrocytes or other cell-specific systems could provide valuable mechanistic insight into the origins of the plasma CE phenotype. However, because the CE differences were observed primarily in plasma rather than brain tissue, and because the current study was not designed to dissect the cellular mechanisms underlying CE metabolism, we do not believe the existing data support a specific mechanistic conclusion regarding astrocyte-intrinsic cholesterol esterification or *APOE*-dependent lipid export.

Accordingly, we have revised the manuscript to avoid assigning a specific mechanism to the reduced plasma CE species and now present these findings as *APOE* genotype-associated lipidomic alterations whose biological origins remain to be determined.

7. The distinction between “FA” and “FFA” is insufficiently defined in the main text. The authors should provide explicit operational definitions.

--- We thank the reviewer for pointing this out. We agree that the distinction between FA and FFA was not sufficiently defined in the original manuscript. In the original text, “FA” referred specifically to fatty acyl chains incorporated within triacylglycerol (TAG), rather than to unesterified free fatty acids (FFA). To avoid confusion, we have revised the terminology throughout the manuscript to “Fatty acid (FA) in TAG chain.”

In the lipidomics analysis, ‘Fatty acid (FA) in TAG chain’ were distinguished from FFAs based on their detection as fragments derived from intact TAG parent ions. TAG molecular species were analyzed in positive-ion ESI-MS/MS, and fatty acyl chains within TAG were identified by neutral-loss scanning of specific fatty acids from the corresponding TAG parent ion (PMID: 11476549). Therefore, the FA signal assigned to TAG reflects fatty acyl chains esterified to the glycerol backbone of TAG, rather than FFAs. FFAs are measured as a separate lipid

class/feature and are not assigned to TAG unless they are detected through the TAG parent ion and neutral-loss relationship. We have added these operational definitions and the relevant reference to the Methods section (**Page 32, line 752-757**).

If the altered omics profiles are being used to support a mechanistic claim, substantial additional functional validation would be required, such as cell culture experiments, plasma transfer studies, or knockout-based approaches targeting key molecules.

--- We thank the reviewer for this important comment. We agree that establishing a direct mechanistic role for the altered transcriptomic, proteomic, or lipidomic signatures would require additional experimental approaches, such as targeted perturbation studies, cell culture systems, plasma transfer experiments, or genetic manipulation of candidate pathways.

Our intention was not to claim that the omics datasets establish causality. Rather, we used these datasets to identify molecular signatures associated with *APOE* genotype and to determine whether the pathological differences observed among genotypes were reflected at the transcriptomic, proteomic, and lipidomic levels.

In response to the reviewer's concern, we have added several new validation experiments, including validation of representative proteomic findings (CD63 and Nectin1), correlation analyses linking DAM-associated gene expression with amyloid burden and *APOE* expression, additional vascular assessments, and further characterization of oligodendrocyte-lineage markers. We have also revised the manuscript throughout to more clearly distinguish association from mechanism and to present the omics findings as hypothesis-generating rather than causal.

We agree that the pathways identified in these analyses provide a framework for future mechanistic studies and have expanded the Discussion (**Page 20, line 485-505, Page 22, line 519-529 and line 536-554**) to highlight specific experimentally testable hypotheses arising from the current findings.

Minor

1. The authors repeated emphasis on “nominal trends” or “nominally significant” observations in the text. For example, effects with p-values such as 0.02 or 0.018 are described in a way that supports a strong narrative around soluble Aβ or neuritic toxicity, despite not meeting the authors’ own corrected significance threshold. This gives the impression that marginal results are being interpreted more aggressively than the statistical framework would justify.

--- We thank the reviewer for this important comment and agree that our original wording may have placed undue emphasis on findings that did not meet the predefined multiple-comparison corrected significance threshold.

As noted by the reviewer, several observations reached nominal significance (uncorrected $p < 0.05$) but did not remain significant after Bonferroni correction. While we considered these findings potentially informative, we agree that they should not be interpreted with the same level of confidence as results that met the corrected significance threshold.

In response, we have revised the manuscript throughout to clearly distinguish statistically significant findings from non-significant observations. Results that did not survive multiple-comparison correction are now described as not significant, and we have removed language that could be interpreted as implying biological conclusions based primarily on nominal significance. We have also carefully reviewed the Results and Discussion to ensure that the major conclusions are supported by findings that meet the predefined statistical criteria.

Altogether, we believe that the revised manuscript now more clearly defines the scope of the study and its principal contribution: a comprehensive analysis of *APOE* allele interactions in heterozygous *APOE* genotypes, particularly the clinically relevant *APOE2/4* genotype. We hope that the revisions have adequately addressed the concerns raised and have strengthened the manuscript for consideration in *Nature Communications*.

We thank you again for your time and consideration.

Sincerely,

Na Zhao

Na Zhao, M.D., Ph.D.,
Associate Professor of Neuroscience
Mayo Clinic Jacksonville

RESPONSE TO REVIEWERS' COMMENTS

Reviewer #1 (Remarks to the Author):

The reviews have been sufficiently addressed regarding sex specific analyses and added as Supplementary figures. Further Figure 4 and 5 have been amended.

--- We thank the reviewer for their careful evaluation of the revised manuscript and appreciate their recognition that the concerns regarding sex-specific analyses and the revisions to Figures 4 and 5 have been adequately addressed.

Reviewer #2 (Remarks to the Author):

The authors have made a strong effort to address my concerns through textual revisions, and the clarifications are helpful, particularly in the pathology sections. However, they do not provide additional experimental evidence to support many of their claims. The proteomics and lipidomics sections also still feel somewhat like add-ons and are not fully integrated into the manuscript. The connection to the earlier sections and to the broader pathobiology remains unclear.

Overall, I think the work is important in showing the effects of heterozygous genotypes. At the same time, the manuscript currently reads more as a collection of observations about the model than as a unified mechanistic study. As a result, the conceptual advance beyond prior studies of homozygous models, increased APOE2 expression, or APOE isoform-switching approaches is not yet fully clear (PMID: 41219507; PMID: 38504517; PMID: 24259049). The authors may wish to more explicitly define what is uniquely learned from the heterozygous model and how these findings extend the existing literature.

--- We thank the reviewer for this thoughtful assessment. We agree that the significance of the heterozygous *APOE* model should be articulated more clearly. We have therefore revised the manuscript to distinguish the biological question addressed by our study from those addressed by previous homozygous, *APOE2* overexpression, and *APOE* isoform-switching studies.

An important distinction of the present study is the simultaneous endogenous expression of two different human *APOE* alleles throughout life. Studies using homozygous *APOE* knock-in models have been essential for defining isoform-specific effects, whereas viral *APOE2* delivery, *APOE2* gene therapy, and inducible *APOE4*-to-*APOE2* switching have demonstrated that increasing *APOE2* expression or replacing *APOE4* with *APOE2* can ameliorate AD-related phenotypes. However, these approaches do not directly establish how two different endogenous *APOE* alleles interact when co-expressed in the naturally heterozygous state.

Importantly, our findings primarily show that *APOE2* substantially attenuates several *APOE4*-associated brain phenotypes in the heterozygous *APOE2/4* state, including parenchymal Aβ accumulation, maladaptive microglial responses, and immune/synaptic proteomic alterations. These findings extend previous studies showing beneficial effects of increasing *APOE2* expression or converting *APOE4* to *APOE2* by demonstrating that endogenous *APOE2* can also attenuate major *APOE4*-associated brain phenotypes when the two alleles are naturally co-expressed. This further supports the therapeutic rationale for strategies aimed at enhancing APOE2-related functions in the context of APOE4.

At the same time, this attenuation is not uniform across all phenotypes. *APOE2/4* mice did not exhibit the increased MBP abundance observed in *APOE2/3* mice and retained several peripheral lipid alterations shared with *APOE3/4* mice. Thus, while *APOE2* exerts a substantial attenuating influence on several *APOE4*-associated amyloid phenotypes, it does not uniformly override *APOE4* across all biological processes. These findings suggest that the relative influence of the two alleles is phenotype- and compartment-specific, a feature that cannot be readily inferred from homozygous or single-isoform manipulation models. We have revised the Discussion accordingly (**Page 18, line 414-428**).

Minor point:

Please review the lipid-species nomenclature and confirm the analytical confidence for the reported TAG species, including TAG C58:1, C59:8, and C60:1. TAG 59:8 would require incorporation of at least one odd-chain fatty acid, which is relatively uncommon in humans, while TAG 60:1 would likely contain multiple long- or very-long-chain fatty acids and may therefore be present at low abundance. Please confirm that these species were detected above the limits of detection and quantification.

--- We thank the reviewer for drawing our attention to this issue. We have carefully re-examined the lipid-species annotations and underlying analytical data. We confirmed that these species were correctly annotated and reliably detected in our dataset.

Reviewer #3 (Remarks to the Author):

The authors added analyses of CD13, GLUT1, ZO-1, and albumin, which improve the vascular characterization of the cohort. However, none showed significant APOE genotype-dependent differences. These negative findings should not be interpreted as evidence of preserved vascular integrity, blood–brain barrier function, or cerebrovascular protection. The text should state only that no detectable differences were observed in the selected markers. More importantly, the discrepancy between the human APOE2–CAA literature and the present mouse findings is not adequately discussed. Human studies have associated APOE2-containing genotypes with increased CAA burden and CAA-related vascular injury, whereas leptomeningeal CAA in *APOE2/3* and *APOE2/4* mice was not significantly different from that in *APOE3/3* mice. The current Discussion interprets comparable CAA levels in *APOE2/4* and *APOE3/3* mice as evidence that APOE2 attenuates APOE4-associated pathology. This interpretation may apply to selected parenchymal amyloid outcomes, but it is too broad when extended to CAA and cerebrovascular pathology. The data support only the narrower conclusion that the increase in leptomeningeal CAA observed in *APOE3/4* mice was not detected in *APOE2/4* mice under the conditions examined. The Discussion should directly address the human–mouse discrepancy, including species differences, limitations of the 5xFAD model, the single age examined, the focus on leptomeningeal CAA, and the distinction between total CAA burden and vessel-wall injury. Conclusions regarding parenchymal Aβ pathology should also be clearly separated from those regarding CAA and cerebrovascular protection.

--- We thank the reviewer for this important clarification. We agree that the absence of significant genotype-dependent differences in CD13, GLUT1, ZO-1, or albumin staining should not be interpreted as evidence of preserved vascular integrity, blood–brain barrier function, or cerebrovascular protection. These measurements assess selected structural and leakage-associated markers and do not provide a comprehensive functional assessment of the cerebrovasculature or BBB.

We have therefore revised the Results to state only that no detectable *APOE* genotype-dependent differences were observed in the vascular markers examined and that no obvious extravascular albumin immunoreactivity was detected under the conditions analyzed. We have removed language implying preserved BBB integrity or cerebrovascular protection.

We also agree that the previous Discussion did not sufficiently address the discrepancy between our mouse findings and the human *APOE2*-CAA literature. Human studies indicate that the relationship between *APOE2* and CAA is distinct from its generally protective association with parenchymal AD pathology. *APOE2* has been associated with greater CAA severity and with CAA-related vasculopathic changes and hemorrhagic complications.

We have revised the manuscript accordingly. Specifically, we now state more narrowly that the increase in leptomeningeal CAA detected in *APOE3/4* mice relative to *APOE3/3* mice was not detected in *APOE2/4* mice at the age examined and under the conditions of this study. We no longer interpret this observation as evidence that *APOE2* broadly protects against CAA or cerebrovascular pathology. We also explicitly discuss several limitations (**Page 22, line 507-516**) relevant to comparison with human CAA, including species-specific differences in cerebrovascular biology, limitations of the rapidly amyloid-depositing 5xFAD model, assessment at a single age, our focus on leptomeningeal CAA rather than a comprehensive analysis of vascular amyloid across vessel types and brain regions, and the distinction between vascular amyloid burden and vessel-wall injury.

Altogether, we believe that these revisions have addressed the remaining concerns raised by the reviewers and have further strengthened the framework, integration, and interpretation of the study. We sincerely thank you and the reviewers for the thoughtful and constructive comments throughout the review process, which have substantially improved the manuscript.

We appreciate your consideration of our revised manuscript and look forward to its publication in *Nature Communications.*

Sincerely,

Na Zhao

Na Zhao, M.D., Ph.D.
Consultant & Associate Professor of Neuroscience
Mayo Clinic Jacksonville
