# Supplementary material for: APOE2 attenuates APOE4-associated amyloid pathology and related toxicity in a mouse model of Alzheimer’s disease
Source: Nat Commun. 2026 Oct 8;17:11438. doi: 10.1038/s41467-026-78407-x (PMC13737102; doi:10.1038/s41467-026-78407-x)
Supplement: Supplementary file 4 [file 41467_2026_78407_MOESM2_ESM.docx]

**Description of Additional Supplementary Files**

File Name: Supplementary Data 1

Description: NanoString glial gene-expression data and differential expression analyses.

File Name: Supplementary Data 2

Description: Brain proteomic data, differential protein analyses, pathway enrichment analyses, and Weighted Gene Co-expression Network Analysis (WGCNA).

File Name: Supplementary Data 3

Description: Plasma lipidomic data, differential lipid analyses, and WGCNA.

File Name: Supplementary Data 4

Description: Targeted brain cholesterol ester (CE) and free fatty acid (FFA) lipidomic data and differential lipid analyses.
